# Synthesis and characteristics of (Hydrogenated) ferulic acid derivatives as potential antiviral agents with insecticidal activity

**DOI:** 10.1186/1752-153X-7-33

**Published:** 2013-02-14

**Authors:** Guang-Ying Huang, Can Cui, Zhi-Peng Wang, Yong-Qiang Li, Li-Xia Xiong, Li-Zhong Wang, Shu-Jing Yu, Zheng-Ming Li, Wei-Guang Zhao

**Affiliations:** 1 State Key Laboratory of Elemento-Organic Chemistry, National Pesticide Engineering Research Center (Tianjin), Nankai University, Tianjin 300071, China

**Keywords:** Synthesis, Ferulic acid amide, Antiviral activity, Insecticidal activity, Tobacco mosaic virus

## Abstract

**Background:**

Plant viruses cause many serious plant diseases and are currently suppressed with the simultaneous use of virucides and insecticides. The use of such materials, however, increases the amounts of pollutants in the environment. To reduce environmental contaminants, virucides with insecticidal activity is an attractive option.

**Results:**

A series of substituted ferulic acid amide derivatives 7 and the corresponding hydrogenated ferulic acid amide derivatives 13 were synthesized and evaluated for their antiviral and insecticidal activities. The majority of the synthesized compounds exhibited good levels of antiviral activity against the tobacco mosaic virus (TMW), with compounds 7a, 7b and 7d in particular providing higher levels of protective and curative activities against TMV at 500 μg/mL than the control compound ribavirin. Furthermore, these compounds displayed good insecticidal activities against insects with piercing-sucking mouthparts, which can spread plant viruses between and within crops.

**Conclusions:**

Two series of ferulic acid derivatives have been synthesized efficiently. The bioassay showed title compounds not only inhibit the plant viral infection, but also prevented the spread of plant virus by insect vectors. These findings therefore demonstrate that the ferulic acid amides represent a new template for future antiviral studies.

## Background

To date, more than 1000 plant viruses, which are grouped into 73 genera and 49 families, have been reported in the literature. Many of these viruses cause a significant number of serious plant diseases, resulting in an estimated $60 billion (USD) loss in crop yields worldwide each year [1]. Although plant viruses are relatively simple at the genetic level, it remains difficult to prevent or control their spread, and plant viruses can therefore have a devastating impact on crop growth. Consequently, plant viruses themselves have often been referred to as “plant cancer”.

Several different chemotherapeutic agents, including synthetic nucleosides such as ribavirin [2], tiazofurin [3], selenazofurin, and benzamide riboside; non-nucleoside-type compounds such as dufulin [4], and mycophenolic acid; and natural products such as ningnanmycin [5], have been reported to effectively inhibit virus replication and suppress the virus symptoms [6,7]. These compounds, however, do not prevent plants from becoming infected. Many viruses are spread by insect vectors between and within crops. Strategies which suppress insect populations below a threshold level could potentially reduce the damage caused by transmitted plant viruses [8]. Aphids, mites and nematodes are the largest and most significant insect vectors. Insecticides, acaricides and nematicides have all been used successfully to prevent or at least reduce the spread of viruses [9,10]. Consequently, crop producers must combine several different tactics to achieve effective levels of protection against the transmission and replication of viruses. We envisage that a combination of virucides and insecticides will become an attractive choice for the treatment of plant viruses. The simultaneous use of virucides and insecticides, however, leads to more polluting contaminants being released into the environment. Therefore to minimize environmental pollution, the use of virucides with insecticidal activity would undoubtedly represents a promising way to treat of plant viruses.

**Figure 1:**
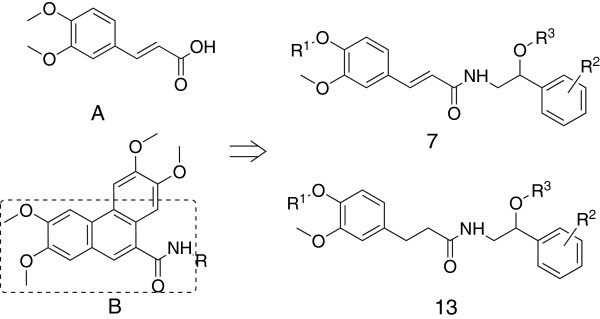
Chemical structures of Ferulic acid A, tylophorine derivatives B and their analogues.

Ferulic acid A (Figure 1) is a natural phenolic compound that can be isolated from many staple foods, including fruits, vegetables, cereals, and coffee. This compound and its derivatives exhibit a wide range of therapeutic effects [11], with applications including anticancer [12,13], anti-diabetic [14], cardio protective [15], neuroprotective [16], and anti-inflammatory activities [17,18]. Phenanthrene-based tylophorine derivatives B [19] (Figure 1) possessing ferulic acid amide substructures have been reported to exhibit excellent levels of antiviral activity. Octopamine is present in high concentrations in various insect tissues. The octopaminergic system in insects performs insecticidal action with minimum non-target effects [20]. Many octopamine derivatives exhibit moderate insecticidal activity [20-24]. With this in mind, we investigate the antiviral activities of feruloyl octopamine derivatives.

We report two series of compounds, including the substituted ferulic acid amide derivatives 7 and the corresponding hydrogenated ferulic acid amide derivatives 13. Both series were evaluated by random screening and possess excellent levels of antiviral activity, together with good levels of insecticidal activity.

## Results and discussion

### Chemistry

**Scheme 1:**
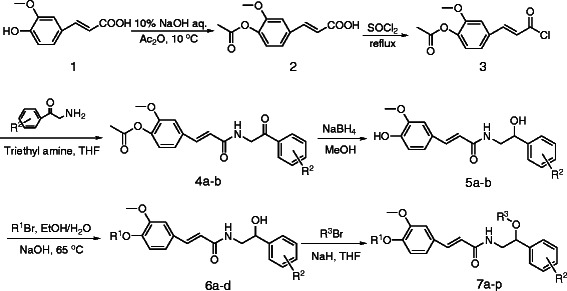
Synthesis of ferulic acid amide derivatives 7.

All of the ferulic acid amide derivatives were prepared from ferulic acid 1 according to the synthetic route in Scheme 1.

Ferulic acid 1 was acetylated with acetic anhydride in aqueous sodium hydroxide solution in the absence of a protecting group on the carboxylic acid. The resulting *O*-acetylferulic acid 2 was then transformed into the corresponding acid chloride 3 by reaction with thionyl chloride. Compound 3 was then readily converted to the ferulic acid amide derivatives 4 via reaction with a series of substituted 2-amino-1-phenylethanones, which were obtained by the reaction of 2-bromo-1-phenylethanone with urotropine. The carbonyl compound 4 was then reduced to the corresponding alcohol with sodium borohydride and the acetyl group subsequently hydrolyzed with NaOH in a one-pot synthesis to give the phenolic compound 5. Alkylation of the phenolic hydroxyl group of compound 5 yielded compound 6, which was subsequently alkylated with a bromoalkane, using NaH to generate the necessary requisite alkoxide, to give the *N*-(2-alkoxy-2-phenylethyl) ferulic acid amide derivatives 7.

**Scheme 2:**
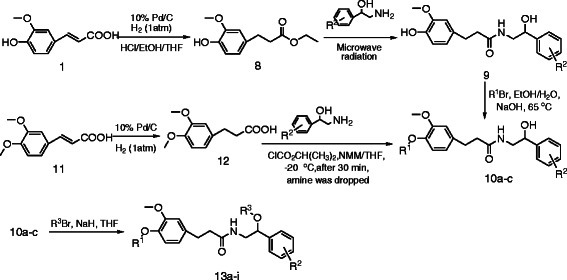
Synthesis of hydrogenated ferulic acid amide derivatives 13.

The corresponding hydrogenated ferulic acid amide derivatives were prepared in an analogous manner via the two key hydrogenated intermediates ethyl ferulate 8 and hydrogenated ferulic acid 11, as shown in Scheme 2.

The hydrogenation reaction of ferulic acid only proceeded in the presence of HCl, which effectively facilitated the concomitant esterification of the carboxylic acid to the corresponding ethyl ester. Thus, the phenolic ester 8 was prepared by catalytic hydrogenation of the ferulic acid 1 using Pd/C and H_2_ (1 atm) in the presence of concentrated HCl. Compound 8 acted as a key intermediate in the synthesis in which R^2^ was always the same. Traditional methods for the synthesis of amides suffer from several disadvantages, including long reaction times and handling issues. The synthesis of amide 9 occurred via a reactive acid chloride intermediate, and this synthesis requires many reaction steps involving hydrolysis, hydroxyl protection, acyl chloride formation, amidation and deprotection reactions. Attempts to reduce the numbers of steps via the direct condensation of the ester 8 were unsuccessful, even at high temperatures (160–180°C) under solvent-free conditions. Microwave irradiation has been used to successfully reduce the chemical reaction time as well as the occurrence of side reactions, whilst increasing yields and improving reproducibility. Through our research, we developed an eco-friendly and high-yielding procedure for the synthesis of ferulic acid amides 9 via the hydrogenation of ethyl ferulate 8 followed by amidation with 2-amino-1-phenylethanol under microwave irradiation at 130°C for 30 min in free-solvent conditions. The alkylation product 10 was obtained by alkylation of the phenolic hydroxyl group of compound 9 with bromoalkane and NaOH.

When R^1^ was a methyl group, the saturated acid 12 was readily prepared by catalytic hydrogenation of ferulic acid 11 using Pd/C and H_2_ (1 atm) in the absence of concentrated HCl. The mixed carbonic anhydride method is a simple and mild amidation method, which has been used extensively for the activation of acyl peptides and subsequent formation of peptide bonds without the occurrence of racemization. Amide 10 (R^1^ = methyl) was prepared according to “the mixed anhydride” method in satisfactory yield. The use of alternative amidation methods, including acid chloride formation and the use of *N,N'*-dicyclohexylcarbodiimide (DCC), provided poor results in comparison.

The reaction of compound 10 with a bromoalkane following the deprotonation of 10 with NaH gave the *N*-(2-alkoxy-2-phenylethyl) hydrogenated ferulic acid amide derivative 13 by alkylation.

The structures of all new compounds were confirmed by their spectra (1H NMR, 13 NMR and HRMS) data [see Additional file 1].

### Biological activity

To evaluate the antiviral activities of the synthesized compounds, the compounds were evaluated relative to the commercially available plant virucide Ribavirin, which was used as a control. The *in vivo* antiviral bioassay against tobacco mosaic virus (TMV) was assayed according the method previously reported in the literature [25], and the antiviral results of all synthesized compounds against TMV are listed in Table 1. The results showed tha the synthesized ferulic acid amide derivatives exhibited varying degrees of activity against TMV. Compounds 7a and 7 m exhibited higher levels of protection activity (40.7 and 34.2%, respectively) than Ribavirin at 500 μg/mL (32.6%). Compound 7b showed the same level of protection activity (32.4%) as Ribavirin at 500 μg/mL (32.6%). Compounds 7a and 7d exhibited higher levels of curative activity (46.4 and 39.5%, respectively) than Ribavirin at 500 μg/mL (38.5%). Compounds 7b and 7 m showed a similar curative activity level (37.2 and 36.9%, respectively) to Ribavirin at 500 μg/mL.

**Table 1:** ***In vivo *** **insecticidal activities and antiviral activities of the investigated compounds**

**Compd.**	**R**^**2**^	**R**^**1**^	**R**^**3**^	**Insecticidal activities**			**Anti-TMV activities**	
				***A. fabae Scopoli***	***T. cinnabarinus***	***C. pipiens pallens***	**Protection effect**	**Curative effect**
				**200 μg/mL**	**200 μg/mL**	**2 μg/mL**	**500 μg/mL**	**500 μg/mL**
**7a**	H	*n*-Pr	propynyl	78	70	30	40.7	46.4
**7b**	*p-*Cl	Me	Me	90	70	70	32.4	37.2
**7c**	*p-*Cl	Me	Et	48	50	60	0	18.6
**7d**	*p-*Cl	Me	propynyl	81	70	10	24.8	39.5
**7e**	*p-*Cl	Me	allyl	70	0	60	0	28.5
**7f**	*p-*Cl	Me	benzyl	92	60	10	0	0
**7g**	*p-*Cl	*n*-Pr	Me	0	0	40	24.3	27.7
**7h**	*p-*Cl	*n*-Pr	Et	70	70	30	5.3	12.6
**7i**	*p-*Cl	*n*-Pr	*n*-Pr	20	20	40	11.3	30.7
**7j**	*p-*Cl	*n*-Pr	propynyl	50	60	100	0	0
**7k**	*p-*Cl	*n*-Pr	allyl	71	75	60	0	5.6
**7l**	*p-*Cl	*n*-Pr	benzyl	0	50	50	13.4	0
**7m**	*p-*Cl	propynyl	Me	20	30	30	34.2	36.9
**7n**	*p-*Cl	propynyl	propynyl	0	0	60	21	22.7
**7o**	*p-*Cl	allyl	allyl	0	0	40	-^a^	-
**7p**	*p-*Cl	benzyl	benzyl	0	0	60	17.5	23.8
**13a**	H	Me	benzyl	0	0	-	7.8	23.5
**13b**	*p-*Cl	Me	propynyl	0	0	-	27.3	30.4
**13c**	*p-*Cl	Me	benzyl	0	0	-	0	7.6
**13d**	*p-*Cl	*n*-Pr	Me	57	50	10	20.6	25.2
**13e**	*p-*Cl	*n*-Pr	Et	18	10	40	17.3	0
**13f**	*p-*Cl	*n*-Pr	propynyl	91	70	30	12.9	10.2
**13g**	*p-*Cl	*n*-Pr	allyl	58	25	40	16.5	12.7
**13h**	*p-*Cl	*n*-Pr	benzyl	92	75	50	27.6	21.6
**13i**	*p-*Cl	propynyl	propynyl	42	30	40	14.5	19.6
chlorfenapyr				100	100	100	-	-
Ribavirin				-	-	-	32.6	38.5

^a^No tested.

When the double bond of the ferulic acid amide was hydrogenated, most of compounds 13 exhibited weaker levels of antiviral activity than the corresponding ferulic acid amides 7. Surprisingly, compound 13b also demonstrated the same excellent levels of protection (27.3%) and curative (30.4%) activity as the ferulic acid amides 7.

To prevent the spread of viruses by piercing-sucking insects between and within tobacco plants, the insecticidal activities of the two series were also determined against *Aphis fabae Scopoli*, *Tetranychus cinnabarinus* and *Culex pipiens pallens* larvae (Table 1). Of the ferulic acid amide derivatives 7a–p, the analogues where R^2^ was a chlorine atom, R^1^ was a methyl group and R^3^ was either a methyl, propynyl or benzyl group (7b, 7d and 7f) demonstrated excellent insecticidal activity against *A. fabae Scopoli* at 200 μg/mL (90, 81, and 92%, respectively), whereas the analogues where R^2^ was a chlorine atom, R^1^ was *n*-propyl, and R^3^ was either a ethyl, propynyl or allyl group (7 h, 7j and 7 k) displayed good insecticidal activity (70, 50, and 71%, respectively). In a separate experiment, all of the active compounds identified above exhibited good insecticidal activity against *T. cinnabarinus*. The insecticidal activities of compounds 7b, 7d, 7f, 7 h, 7j and 7 k against *T. cinnabarinus* at 200 μg/mL were 70, 70, 60, 70, 60 and 75%, respectively. When R^1^ was a hydrogen atom, compound 7a showed good insecticidal activities against the two insects. In contrast, the, compounds bearing an unsaturated substituent on R^1^ (7 m–7p), proved to be virtually inactive against *A. fabae Scopoli* and *T. cinnabarinus*. The most active of the synthesized compounds also showed good larvicidal activities against *C. pipiens pallens* at 2 μg/mL, with compound 7i exhibiting 100% inhibition at 2 μg/mL.

Hydrogenation of the double bond of the ferulic acid amides gave the corresponding saturated analogues 13, which showed excellent insecticidal activities against the two insects when R^2^ was a propyl group. When the propyl group on R^2^ was replaced with methyl, the compounds showed low levels of activity. Compounds 13f and 13 h, where R^3^ was either a propynyl or benzyl group, exhibited higher levels of insecticidal activity than all of the other compounds against *A. fabae Scopoli* (91 and 92%, respectively) and *T. cinnabarinus* (70 and 75%, respectively) at 200 μg/mL.

Compounds 7a, 7b and 7d indicated excellent protection and curative activities against TMV at 500 μg/mL. Furthermore, they exhibited good insecticidal activities against insects with piercing-sucking mouthparts at 200 μg/mL. Although the two series (compounds 7 and 13) had weaker insecticidal activities than that of the commercial pesticide, they effectively controlled replication of the plant virus whilst simultaneously preventing insects with piercing-sucking mouthparts, spreading the plant virus between and within crops, at 500 μg/mL.

## Experimental

### Instruments

The ^1^H and ^13^C NMR spectra were obtained at 400 and 100 MHz, respectively, on a Bruker Avance 400 spectrometer with tetramethylsilane (TMS) as an internal standard. High resolution mass spectrometry (HRMS) data were obtained on a VG ZAB HS instrument. The microwave synthesis was performed in a CEM Discover focused microwave (2450 MHz, 300 W). Melting points were determined on an X-4 binocular microscope melting point apparatus (Beijing Tech Instruments Co., Beijing, China) and were uncorrected. All chemicals and reagents were purchased from standard commercial suppliers. All solvents and liquid reagents were dried according to the standard methods and distilled prior to use.

### Synthesis of compounds

#### Preparation of O-acetylferulic acid 2

Compound 2 was prepared as a white solid according to a literature procedure [26].

#### Preparation of ferulic acid amide derivatives 4

Compound 2 (11.8 g, 50 mmol) was dissolved in SOCl_2_ (23.8 g) and refluxed for 4 h. The resulting solution was distilled *in vacuo* to give compound 3 as a pale yellow solid.

Substituted 2-amino-1-phenylethanone (60 mmol) was suspended in a mixture of THF (400 mL) and Et_3_N (12.1 g, 120 mmol). The mixture was stirred for 20 min at room temperature and then cooled to −10°C. A solution of compound 3 (50 mmol) in THF (100 mL) was then added to the reaction mixture at this temperature. The reaction mixture was warmed to ambient temperature and stirred for 24 h. The resulting mixture was filtrated to remove the white precipitation, and the filtrate collected and distilled *in vacuo* to give the crude product as a solid that was purified by crystallization to provide the compound 4.

4a: R_2_ = Cl, white solid, m.p.: 183–185°C; ^1^H NMR (400 MHz, DMSO) *δ* 2.24 (s, 3H, COCH_3_), 3.80 (s, 3H, OC**H**_3_), 4.72–4.73 (m, 2H, NHC**H**_2_), 6.83 (d, 1H, *J =* 16.0 Hz, COC**H**), 7.10–7.34 (m, 3H, Ar-**H**), 7.42 (d, 1H, *J =* 16.0 Hz, Ar-C**H**), 7.52–7.76 (m, 4H, Ar-**H**), 8.48 (br, 1H, N**H**).

**4b:** R_2_ = H, white solid, m.p.:149–151°C; ^1^H NMR (400 MHz, DMSO) *δ* 2.31 (s, 3H, COC**H**_3_), 3.94 (s, 3H, OC**H**_3_), 4.91–4.92 (m, 2H, NHC**H**_2_), 5.89 (br, 1H, N**H**), 6.43 (d, 1H, *J =* 16.0 Hz, COC**H**), 6.75 (br, 1H, O**H**), 6.91–7.10 (m, 3H, Ar-**H**), 7.50–7.66 (m, 4H, ArC**H**+Ar-**H**), 8.01–8.03 (m, 2H, Ar-**H**).

#### Preparation of compound 5

Sodium borohydride was added to a solution of compound 5 (2.5 mmol) in MeOH (40 mL) in a portion-wise manner and the resulting mixture was stirred at ambient temperature for 4 h. Water (10 mL) was then added and the mixture was heated to 60°C for 1 h. The mixture was then cooled to ambient temperature and the pH adjusted to pH 6–7 with 2 N aqueous HCl to give a white precipitate that was collected by filtration, and purified by recrystallization to provide compound 5.

**5a:** R_2_ = Cl, white solid, m.p.: 186–189°C; ^1^H NMR (400 MHz, DMSO) *δ* 3.14–3.44 (m, 3H, NHC**H**_2_+CHO**H**), 3.77 (s, 3H, OC**H**_3_), 4.63 (dd, 1H, *J =* 4.8 Hz, *J =* 7.6 Hz, OC**H**), 6.02 (br, 1H, N**H**), 6.26 (d, 1H, *J =* 16.0 Hz, COC**H**), 6.84–7.09 (m, 3H, Ar-**H**), 7.26–7.33 (m, 4H, Ar-**H**), 7.60 (d, 1H, *J =* 16.0 Hz, Ar-C**H**), 9.42 (br, 1H, ArO**H**).

**5b:** R_2_ = H, white solid, m.p.: 150–153°C; ^1^H NMR (400 MHz, DMSO) *δ* 3.94 (s, 3H, OC**H**_3_), 4.91–4.92 (m, 3H, NHC**H**_2_+OC**H**), 5.89 (br, 1H, N**H**), 6.43 (d, 1H, *J =* 16.0 Hz, COC**H**), 6.75 (br, 1H, O**H**), 6.91–7.10 (m, 3H, Ar-**H**), 7.50–7.66 (m, 4H, Ar-C**H**+Ar-**H**), 8.01–8.03 (m, 2H, Ar-**H**).

#### Preparation of the compounds 6

Sodium hydroxide (0.90 g, 22 mmol) and compound 5 (20 mmol) were dissolved in a mixture of ethanol (120 mL) and water (40 mL) and the resulting mixture was stirred at ambient temperature for 30 min. Alkyl bromide was then added slowly at ambient temperature. The reaction mixture was then stirred at 60–70°C for 10 h. The mixture was then cooled to ambient temperature and acidified to pH 1 with 2 N aqueous HCl, before being concentrated *in vacuo*, filtered, washed with water, dried, and recrystallized to give the title compound 6.

**6a:** R_2_ = Cl, R_1_ = *n*-Pr, white solid, m.p.: 194–196°C; ^1^H NMR (400 MHz, DMSO) *δ* 0.97 (t, 3H, *J =* 7.6 Hz, CH_2_C**H**_3_), 1.67–1.77 (m, 2H, CH_3_C**H**_2_), 3.23–3.30,3.37–3.43 (m, 2H, NHC**H**_2_), 3.79 (s, 3H, OC**H**_3_), 3.94 (t, 2H, *J =* 6.4 Hz, ArOC**H**_2_), 4.65–4.69 (m, 1H, OC**H**), 5.63–5.64 (m, 1H, O**H**), 6.58 (d, 1H, *J =* 16.0 Hz, COC**H**), 6.96–7.14 (m, 3H, Ar-**H**), 7.31–7.41 (m, 4H, Ar-C**H**+Ar-**H**), 8.04 (t, 1H, *J =* 5.2 Hz, N**H**).

**6b:** R_2_ = Cl, R_1_ = Me, white solid, m.p.: 195–197°C; ^1^H NMR (400 MHz, DMSO) *δ* 2.48 (br, 1H, O**H**), 3.41–3.45 (m, 1H, NHC**H**_2_), 3.80–3.99 (m, 7H, NHC**H**_2_+*m*-ArOC**H**_3_+*p*-ArOC**H**_3_), 4.91–4.92 (m, 1H, OC**H**), 6.02 (br, 1H, N**H**), 6.26 (d, 1H, *J =* 16.0 Hz, COC**H**), 6.84–7.09 (m, 3H, Ar-**H**), 7.26–7.33 (m, 4H, Ar-**H**), 7.60 (d, 1H, *J =* 16.0 Hz, Ar-C**H**).

**6c:** R_2_ = Cl, R_1_ = propynyl, white solid, m.p.: 180–182°C; ^1^H NMR (400 MHz, DMSO) *δ* 3.24–3.44 (m, 3H, NHC**H**_2_+C≡C**H**), 3.59 (br, 1H, O**H**), 3.81 (s, 3H, OC**H**_3_), 4.67 (br, 1H, OC**H**), 4.81–4.82 (m, 2H, OC**H**_2_), 5.64–5.65 (m, 1H, COC**H**) 7.03–7.18 (m, 3H, Ar-**H**), 7.32–7.41 (m, 5H, Ar-**H**+Ar-C**H**), 8.05 (t, 1H, *J* = 5.6 Hz, N**H**)

**6d:** R_2_ = H, R_1_ = *n*-Pr, white solid, m.p.: 140–142°C; ^1^H NMR (400 MHz, DMSO) *δ* 0.94 (t, 3H, *J =* 7.2 Hz, CH_2_C**H**_3_), 1.66–1.74 (m, 2H, CH_3_C**H**_2_), 3.33–3.44 (m, 2H, NHC**H**_2_), 3.77 (s, 3H, OC**H**_3_), 3.90 (t, 2H, *J =* 6.0 Hz, ArOC**H**_2_), 4.62–4.64 (m, 1H, OC**H**), 5.53–5.54 (m, 1H, O**H**), 6.78 (d, 1H, *J =* 16.0 Hz, COC**H**), 6.93–7.11 (m, 3H, Ar-**H**), 7.22–7.24 (m, 1H, Ar-C**H**), 7.29–7.33 (m, 5H, Ar-**H**), 8.03 (br, 1H, N**H**).

**10a:** R_2_ = Cl, R_1_ = *n*-Pr, white solid, m.p.: 100–102°C; ^1^H NMR (400 MHz, CDCl_3_) *δ* 1.02 (t, 3H, *J =* 7.2 Hz, CH_2_C**H**_3_), 1.80–1.88 (m, 2H, CH_3_C**H**_2_), 2.46 (t, 2H, *J =* 7.2 Hz, COC**H**_2_), 2.89 (t, 2H, *J =* 7.2 Hz, Ar-C**H**_2_), 3.58–3.63 (m, 2H, NHC**H**_2_), 3.84 (s, 3H, OC**H**_3_), 3.94 (t, 2H, *J =* 6.8 Hz, ArOC**H**_2_), 4.73–4.74 (m, 1H, OC**H**), 5.75 (br, 1H, N**H**), 6.68–6.80 (m, 3H, Ar-**H**), 7.20–7.29 (m, 4H, Ar-**H**).

**10b:** R_2_ = H, R_1_ = Me, yellow solid, m.p.: 94–95°C; ^1^H NMR (400 MHz, CDCl_3_) *δ* 2.50 (t, 2H, *J* = 7.2 Hz, CH_2_C**H**_2_), 2.94 (t, 2H, *J* = 7.2 Hz, CH_2_C**H**_2_), 3.28–3.35 (m, 1H, CHC**H**_2_), 3.65–3.72 (m, 1H, CHC**H**_2_), 3.88 (s, 3H, *o*-ArOC**H**_3_), 3.89 (s, 3H, *p*-ArOC**H**_3_), 4.80 (dd, 1H, *J* = 2.8 Hz, CH_2_C**H**), 5.85 (br, 1H, CON**H**), 6.74–6.83 (m, 3H, Ar-**H**), 7.31–7.36 (m, 5H, Ar-**H**).

#### Preparation of the compounds 7

Sodium hydride (70 mg, 3.0 mmol) was added to a solution of compound 6 (or 10) (2.5 mmol) in dry THF (70 mL), and the resulting mixture was stirred under N_2_ at ambient temperature for 1 h. The alkyl bromide (3.7 mmol) was then added and the resulting mixture agitated at ambient temperature for 24 h. The reaction mixture was distilled to dryness *in vacuo* to give a residue that was purified by flash chromatography eluting with petroleum ether-EtOAc (3: 2) to give compound 7.

**7a:** R_2_ = H, R_1_ = *n*-Pr, R_3_ = propynyl, white solid, m.p.:91–92°C; ^1^H NMR (400 MHz, CDCl_3_) *δ* 1.03 (t, 3H, *J* = 7.2 Hz, CH_2_C**H**_3_), 1.82–1.91 (m, 2H, CH_3_C**H**_2_), 2.43 (s, 1H, CH_2_C≡C**H**), 3.28–3.34 (m, 1H, NHC**H**_2_), 3.88–3.95 (m, 5H, ArOC**H**_3_+NHC**H**_2_+CH≡CC**H**_2_), 3.99 (t, 2H, *J* = 6.8 Hz, ArOC**H**_2_), 4.16 (d, 1H, *J* = 16.0 Hz, CHOC**H**_2_), 4.67 (dd, 1H, *J* = 2.8 Hz, *J* = 9.2 Hz, OC**H**), 6.15 (br, 1H, N**H**), 6.30 (d, 1H, *J* = 16.0 Hz, COC**H**), 6.84 (d, 1H, *J* = 8.0 Hz, Ar-**H**), 7.03–7.07 (m, 2H, Ar-**H**), 7.32–7.39 (m, 5H, Ar-**H**), 7.57 (d, 1H, *J* = 16.0 Hz, Ar-C**H**); ^13^C NMR (101 MHz, CDCl_3_) δ 166.1, 150.2, 149.4, 141.2, 138.1, 128.8, 128.6, 127.6, 126.9, 122.1, 118.3, 112.4, 110.1, 79.7, 79.6, 74.7, 70.4, 56.0, 56.0, 45.6, 22.4, 10.4; HRMS (ESI) (M+Na)^+^ Calcd for C_24_H_27_NO_4_Na^+^ 416.1832, found 416.1838.

**7b:** R_2_ = *p*-Cl, R_1_ = Me, R_3_ = Me, white solid, m.p.: 92–93°C; ^1^H NMR (400 MHz, CDCl_3_) *δ* 3.21–3.29 (m, 4H, CHOC**H**_3_+NHC**H**_2_), 3.80–3.93 (m, 7H, *m*-ArOC**H**_3_+*p*-ArOC**H**_3_+NHC**H**_2_), 4.30 (dd, 1H, *J* = 5.2 Hz, *J* = 8.8 Hz, OC**H**), 6.02 (br, 1H, N**H**), 6.28 (d, 1H, *J* = 16.0 Hz, COC**H**), 6.86 (d, 1H, *J* = 8.0 Hz, Ar-**H**), 7.03–7.10 (m, 2H, Ar-**H**), 7.26–7.36 (m, 4H, Ar-**H**), 7.58 (d, 1H, *J* = 16.0 Hz, Ar-C**H**); ^13^C NMR (101 MHz, CDCl_3_) δ 166.1, 150.6, 149.1, 141.1, 137.7, 133.9, 128.8, 128.1, 127.7, 122.1, 118.4, 111.0, 109.6, 81.8, 56.9, 55.9, 55.8, 45.8; HRMS (ESI) (M+Na)^+^ Calcd for C_20_H_22_ClNO_4_Na^+^ 398.1130, found 398.1138.

**7c:** R_2_ = *p*-Cl, R_1_ = Me, R_3_ = Et, white solid, m.p.:97–99°C; ^1^H NMR (400 MHz, CDCl_3_) *δ* 1.19 (t, 3H, *J* = 6.8 Hz, CH_2_C**H**_3_), 3.23–3.49 (m, 3H, CH_3_C**H**_2_+NHC**H**_2_), 3.79–3.89 (m, 7H, *m*-ArOC**H**_3_+*p*-ArOC**H**_3_+NHC**H**_2_), 4.41 (dd, 1H, *J* = 2.8 Hz, *J* = 8.0 Hz, OC**H**), 6.09 (br, 1H, N**H**), 6.28 (d, 1H, *J* = 16.0 Hz, COC**H**), 6.84 (d, 1H, *J* = 7.2 Hz, Ar-**H**), 7.02 (s, 1H, Ar-**H**), 7.08 (d, 1H, *J* = 7.2 Hz, Ar-**H**), 7.26–7.32 (m, 4H, Ar-**H**), 7.37 (d, 1H, *J* = 16.0 Hz, Ar-C**H**); ^13^C NMR (101 MHz, CDCl_3_) δ 166.1, 150.6, 149.1, 141.1, 138.5, 133.7, 128.8, 128.0, 127.7, 122.1, 118.4, 111.0, 109.6, 79.8, 64.5, 55.9, 55.9, 45.8, 15.3; HRMS (ESI) (M+Na)^+^ Calcd for C_21_H_24_ClNO_4_Na^+^ 412.1286, found 412.1294.

**7d:** R_2_ = *p*-Cl, R_1_ = Me, R_3_ = propynyl, white solid, m.p.:137–138°C; ^1^H NMR (400 MHz, CDCl_3_) *δ* 2.43 (s, 1H, C≡C**H**), 3.25–3.30 (m, 1H, NHC**H**_2_), 3.87–3.99 (m, 8H, *m*-ArOC**H**_3_ + *p*-ArOC**H**_3_+ NHC**H**_2_), 4.16 (d, 1H, *J* = 16.0 Hz, OC**H**_2_), 4.67 (dd, 1H, *J* = 3.6Hz, *J* = 8.8 Hz, OC**H**), 6.09 (br, 1H, N**H**), 6.30 (d, 1H, *J* = 16.0 Hz, COC**H**), 6.84–6.87 (m, 1H, Ar-**H**), 7.03–7.10 (m, 2H, Ar-**H**), 7.29–7.36 (m, 4H, Ar-**H**), 7.58 (d, 1H, *J* = 16.0 Hz, Ar-C**H**); ^13^C NMR (101 MHz, CDCl_3_) δ 166.1, 150.7, 149.1, 141.3, 136.7, 134.3, 129.0, 128.3, 127.7, 122.1, 118.23, 111.1, 109.6, 79.3, 79.0, 75.0, 56.1, 56.0, 55.9, 45.5; HRMS (ESI) (M+Na)^+^ Calcd for C_22_H_22_ClNO_4_Na^+^ 422.1130, found 422.1133.

**7e:** R_2_ = *p*-Cl, R_1_ = Me, R_3_ = allyl, white solid, m.p.:115–116°C; ^1^H NMR (400 MHz, CDCl_3_) *δ* 3.27–3.33 (m, 1H, NHC**H**_2_), 3.82–3.91 (m, 9H, *m*-ArOC**H**_3_+*p*-ArOC**H**_3_+ NHC**H**_2_+OC**H**_2_), 4.49 (dd, 1H, *J* = 3.6Hz, *J* = 8.0 Hz, OC**H**), 5.17–5.27 (m, 2H, CH=C**H**_2_), 5.86–5.92 (m, 1H, CH_2_=C**H**), 6.03 (br, 1H, N**H**), 6.28 (d, 1H, *J* = 16.0 Hz, COC**H**), 6.86 (d, 1H, *J* = 8.0 Hz, Ar-**H**), 7.03 (s,1H, Ar-**H**), 7.09 (d, 1H, *J* = 8.0 Hz, Ar-**H**), 7.28–7.35 (m, 4H, Ar-**H**), 7.57 (d, 1H, *J* = 16.0 Hz, Ar-C**H**); ^13^C NMR (101 MHz, CDCl_3_) δ 166.1, 150.6, 149.1, 141.2, 137.94, 134.3, 133.9, 128.9, 128.1, 127.7, 122.1, 118.3, 117.4, 111.0, 109.6, 79.3, 69.8, 56.0, 55.9, 45.8; HRMS (ESI) (M+Na)^+^ Calcd for C_22_H_24_ClNO_4_Na^+^ 424.1286, found 424.1290.

**7f:** R_2_ = *p*-Cl, R_1_ = Me, R_3_ = benzyl, white solid, m.p.:104–105°C; ^1^H NMR (400 MHz, CDCl_3_) *δ* 3.29–3.36 (m, 1H, NHC**H**_2_), 3.83–3.91 (m, 7H, *p*-ArOC**H**_3_+*m*-ArOC**H**_3_+NHC**H**_2_), 4.30 (d, 1H, *J* = 12.0 Hz, Ar-C**H**_2_), 4.48–4.55 (m, 2H, OC**H**+Ar-C**H**_2_), 5.99 (br, 1H, N**H**), 6.22 (d, 1H, *J* = 16.0 Hz, COC**H**), 6.86 (d, 1H, *J* = 8.0 Hz, Ar-**H**), 7.02 (s, 1H, Ar-**H**), 7.08 (d, 1H, *J* = 8.8 Hz, Ar-**H**), 7.30–7.38 (m, 9H, Ar-**H**), 7.54 (d, 1H, *J* = 16.0 Hz, Ar-C**H**); ^13^C NMR (101 MHz, CDCl_3_) δ 166.1, 150.6, 149.1, 141.1, 137.9, 137.8, 134.0, 128.9, 128.6, 128.2, 127.9, 127.9, 127.7, 122.1, 118.3, 111.1, 109.6, 79.6, 70.9, 56.0, 55.9, 45.8; HRMS (ESI) (M+H)^+^ Calcd for C_26_H_26_ClNO_4_H^+^ 452.1623, found 452.1622.

**7g:** R_2_ = *p*-Cl, R_1_ = *n*-Pr; R_3_ = Me, white solid, m.p.:128–129°C; ^1^H NMR (400 MHz, CDCl_3_) *δ* 0.98 (t, 3H, *J* = 7.2 Hz, CH_2_C**H**_3_), 1.77–1.86 (m, 2H, CH_3_C**H**_2_), 3.16–3.23 (m, 4H, CHOC**H**_3_+NHC**H**_2_), 3.73–3.80 (m, 1H, NHC**H**_2_), 3.83 (s, 3H, ArOC**H**_3_), 3.94 (t, 2H, *J* = 6.8 Hz, ArOC**H**_2_), 4.25 (dd, 1H, *J* = 3.6 Hz, *J* = 8.8 Hz, Ar-C**H**), 5.92 (br, 1H, N**H**), 6.20 (d, 1H, *J* = 16.0 Hz, COC**H**), 6.79 (d, 1H, *J* = 8.8 Hz, Ar-**H**), 6.97–7.02 (m, 2H, Ar-**H**), 7.20–7.30 (m, 4H, Ar-**H**), 7.50 (d, 1H, *J* = 16.0 Hz, Ar-C**H**; ^13^C NMR (101 MHz, CDCl_3_) δ 166.1, 150.3, 149.4, 141.3, 137.7, 133.9, 128.9, 128.1, 127.5, 122.1, 118.1, 112.4, 110.1, 81.8, 70.4, 57.0, 56.0, 45.7, 22.4, 10.4; HRMS (ESI) (M+Na)^+^ Calcd for C_22_H_26_ClNO_4_Na^+^ 426.1443, found 426.1440.

**7h:** R_2_ = *p*-Cl, R_1_ = *n*-Pr, R_3_ = Et, white solid, m.p.:89–91°C; ^1^H NMR (400 MHz, CDCl_3_) δ 0.98 (t, 3H, *J* = 7.2 Hz, OCH_2_C**H**_3_), 1.13 (t, 3H, *J* = 6.8 Hz, OCH_2_CH_2_C**H**_3_), 1.77–1.87 (m, 2H, OCH_2_C**H**_2_), 3.17–3.23 (m, 1H, NHC**H**_2_), 3.27–3.40 (m, 2H, CHOC**H**_2_), 3.71–3.78 (m, 1H, N**H**), 3.83 (s, 3H, OC**H**_3_), 3.94 (t, 2H, *J* = 6.8 Hz, ArOC**H**_2_), 4.35 (dd, 1H, *J* = 3.6 Hz, *J* = 8.8 Hz, OC**H**), 5.93 (br, 1H, N**H**), 6.20 (d, 1H, *J* = 16.0 Hz, COC**H**), 6.78 (d, 1H, *J* = 8.8 Hz, Ar-**H**), 6.97–7.02 (m, 2H, Ar-**H**), 7.21–7.28 (m, 4H, Ar-**H**), 7.50 (d, 1H, *J* = 16.0 Hz, Ar-C**H**); ^13^C NMR (101 MHz, CDCl_3_) δ 166.1, 150.2, 149.4, 141.2, 138.5, 133.7, 128.8, 128.0, 127.6, 122.1, 118.2, 112.4, 110.1, 79.9, 70.4, 64.6, 56.0, 45.8, 22.4, 15.3, 10.4; HRMS (ESI) (M+Na)^+^ Calcd for C_23_H_28_ClNO_4_Na^+^ 440.1599, found 440.1607.

**7i:** R_2_ = *p*-Cl, R_1_ = *n*-Pr, R_3_ = *n*-Pr, white solid, m.p.:81–83°C; ^1^H NMR (400 MHz, CDCl_3_) *δ* 0.89-0.92 (m, 3H, CHOCH_2_CH_2_C**H**_3_), 1.03 (t, 3H, *J* = 6.4 Hz, ArOCH_2_CH_2_C**H**_3_), 1.56–1.61 (m, 2H, CHOCH_2_C**H**_2_), 1.85–1.90 (m, 2H, ArOCH_2_C**H**_2_), 3.25–3.33 (m, 3H, CHOC**H**_2_+NHC**H**_2_), 3.78–3.89 (m, 4H, OC**H**_3_+NHC**H**_2_), 3.99 (t, 2H, *J* = 6.4 Hz, ArOC**H**_2_), 4.40 (dd, 1H, *J* = 2.8 Hz, *J* = 9.2 Hz, OC**H**), 6.04 (br, 1H, N**H**), 6.26 (d, 1H, *J* = 16.0 Hz, COC**H**), 6.84 (d, 1H, *J* = 8.0 Hz, Ar-**H**), 7.02–7.07 (m, 2H, Ar-**H**), 7.26–7.34 (m, 4H, Ar-**H**), 7.56 (d, 1H, *J* = 16.0 Hz, Ar-C**H**); ^13^C NMR (101 MHz, CDCl_3_) δ 166.1, 150.3, 149.4, 141.2, 138.5, 133.7, 128.8, 128.0, 127.6, 122.1, 118.2, 112.4, 110.2, 80.0, 70.9, 70.4, 56.0, 45.9, 23.0, 22.4, 10.7, 10.4; HRMS (ESI) (M+Na)^+^ Calcd for C_24_H_30_ClNO_4_Na^+^ 454.1392, found 454.1392.

**7j:** R_2_ = *p*-Cl, R_1_ = *n*-Pr, R_3_ = propynyl, white solid, m.p.:71–72°C; ^1^H NMR (400 MHz, CDCl_3_) *δ* 1.03 (t, 3H, *J* = 7.2 Hz, CH_2_C**H**_3_), 1.83–1.92 (m, 2H, CH_3_C**H**_2_), 2.43 (s, 1H, C≡C**H**), 3.24–3.30 (m, 1H, NHC**H**_2_), 3.86–3.92 (m, 5H, ArOC**H**_3_+NHC**H**_2_+CH≡CC**H**_2_), 4.00 (t, 2H, *J* = 6.8 Hz, ArOC**H**_2_), 4.15 (d, 1H, *J* = 16.0 Hz, CHOC**H**_2_), 4.66 (dd, 1H, *J* = 2.8 Hz, *J* = 8.8 Hz, OC**H**), 6.10 (br, 1H, N**H**), 6.29 (d, 1H, *J* = 16.0 Hz, COC**H**), 6.84 (d, 1H, *J* = 8.8 Hz, Ar-**H**), 7.03–7.07 (m, 2H, Ar-**H**), 7.28–7.36 (m, 4H, Ar-**H**), 7.57 (d, 1H, *J* = 16.0 Hz, Ar-C**H**); ^13^C NMR (101 MHz, CDCl_3_) δ 166.2, 150.3, 149.4, 141.3, 136.7, 134.3, 129.0, 128.3, 127.5, 122.1, 118.1, 112.4, 110.1, 79.3, 79.0, 75.0, 70.4, 56.1, 56.0, 45.5, 22.4, 10.4; HRMS (ESI) (M+H)^+^ Calcd for C_24_H_26_ClNO_4_H^+^ 428.1623, found 428.1624.

**7k:** R_2_ = *p*-Cl, R_1_ = *n*-Pr, R_3_ = allyl, white solid, m.p.:86–88°C; ^1^H NMR (400 MHz, CDCl_3_) *δ* 1.03 (t, 3H, *J* = 7.2 Hz, CH_2_C**H**_3_), 1.83–1.92 (m, 2H, CH_3_C**H**_2_), 3.26–3.33 (m, 1H, NHC**H**_2_), 3.80–3.95 (m, 6H, OC**H**_3_ + NHC**H**_2_ + CH_2_=CHC**H**_2_), 4.00 (t, 2H, *J* = 6.8 Hz, ArOC**H**_2_), 4.49 (dd,1H, *J* = 3.2 Hz, *J* = 8.4 Hz, OC**H**), 5.17–5.27 (m, 2H, CH=C**H**_2_), 5.84–5.93 (m, 1H, CH_2_=C**H**), 6.03 (br, 1H, N**H**), 6.27 (d, 1H, *J* = 16.0 Hz, COC**H**), 6.85 (d, 1H, *J* = 8.0 Hz, Ar-**H**), 7.03–7.04 (m, 2H, Ar-**H**), 7.27–7.35 (m, 4H, Ar-**H**), 7.57 (d, 1H, *J* = 16.0 Hz, Ar-C**H**); ^13^C NMR (101 MHz, CDCl_3_) δ 166.2, 150.3, 149.4, 141.3, 138.0, 134.3, 133.9, 128.8, 128.1, 127.5, 122.1, 118.1, 117.4, 112.4, 110.1, 79.3, 70.4, 69.8, 56.0, 45.8, 22.4, 10.4; HRMS (ESI) (M+Na)^+^ Calcd for C_24_H_28_ClNO_4_Na^+^ 452.1599, found 452.1603.

**7l:** R_2_ = *p*-Cl, R_1_ = *n*-Pr, R_3_ = benzyl, white solid, m.p.:69–71°C; ^1^H NMR (400 MHz, CDCl_3_) *δ* 1.04 (t, 3H, *J* = 7.2 Hz, CH_2_C**H**_3_), 1.84–1.92 (m, 2H, CH_3_C**H**_2_), 3.29–3.36 (m, 1H, NHC**H**_2_), 3.83–3.90 (m, 4H, OC**H**_3_ + NHC**H**_2_), 4.00 (t, 2H, *J* = 6.8 Hz, ArOC**H**_2_), 4.30 (d, 1H, *J* = 12.0 Hz, Ar-C**H**_2_), 4.48–4.55 (m, 2H, Ar-C**H**_2_ + OC**H**), 6.85 (d, 1H, *J* = 8.8 Hz, Ar-**H**), 7.02–7.07 (m, 2H, Ar-**H**), 7.30–7.38 (m, 9H, Ar-**H**), 7.54 (d, 1H, *J* = 16.0 Hz, Ar-C**H**); ^13^C NMR (101 MHz, CDCl_3_) δ 166.2, 150.3, 149.4, 141.2, 137.9, 137.9, 134.0, 128.9, 128.6, 128.2, 127.9, 127.9, 127.6, 122.1, 118.2, 112.4, 110.2, 79.6, 70.9, 70.4, 56.0, 45.8, 22.4, 10.5; HRMS (ESI) (M+Na)^+^ Calcd for C_28_H_30_ClNO_4_Na^+^ 502.1756, found 502.1757.

**7m:** R_2_ = *p*-Cl, R_1_ = propynyl, R_3_ = Me, white solid, m.p.:134–135°C; ^1^H NMR (400 MHz, CDCl_3_) *δ* 2.47 (s, 1H, C≡C**H**), 3.16–3.23 (m, 4H, CHOC**H**_3_ + NHC**H**_2_), 3.74–3.82 (m, 1H, NHC**H**_2_), 3.85 (s, 3H, ArOC**H**_3_), 4.25 (dd, 1H, *J* = 3.6 Hz, *J* = 8.4 Hz, OC**H**), 4.73 (s, 2H, ArOC**H**_2_), 5.95 (br, 1H, N**H**), 6.23 (d, 1H, *J* = 16.0 Hz, COC**H**), 6.94–6.99 (m, 2H, Ar-**H**), 7.03 (d, 1H, *J* = 8.0 Hz, Ar-**H**), 7.20–7.30 (m, 4H, Ar-**H**), 7.51 (d, 1H, *J* = 16.0 Hz, Ar-C**H**); ^13^C NMR (101 MHz, CDCl_3_) δ 166.0, 149.9, 149.7, 141.2, 137.6, 134.0, 128.9, 128.1, 127.2, 121.6, 118.8, 113.8, 110.2, 81.8, 78.2, 76.3, 57.0, 56.6, 56.0, 45.7; HRMS (ESI) (M+H)^+^ Calcd for C_22_H_22_ClNO_4_H^+^ 400.1310, found 400.1305.

**7n:** R_2_ = *p*-Cl, R_1_ = propynyl, R_3_ = propynyl, yellow solid, m.p.:101–104°C; ^1^H NMR (400 MHz, CDCl_3_) *δ* 2.46 (t, *J* = 2.4 Hz, 1H, CHOCH_2_C≡C**H**), 2.52 (t, *J* = 2.4 Hz, 1H ArOCH_2_C≡C**H**), 3.28–3.34 (m, 1H, NHC**H**_2_), 3.92–3.97 (m, 5H, OC**H**_3_+NHC**H**_2_+CHOC**H**_2_), 4.17 (dd, 1H, *J* = 15.7, 2.4 Hz, CHOC**H**_2_), 4.70 (dd, 1H, *J* = 9.0, 3.5 Hz, OC**H**), 4.82 (d, *J* = 2.3 Hz, 2H, ArOC**H**_2_), 6.09 (br, 1H, N**H**), 6.30 (d, 1H, *J* = 15.5 Hz, COC**H**), 7.04–7.11 (m, 3H, Ar-**H**), 7.28–7.40 (m, 4H, Ar-**H**), 7.60 (d, 1H, *J* = 15.5 Hz, Ar-C**H**); ^13^C NMR (101 MHz, CDCl_3_) δ 166.0, 149.7, 148.3, 141.1, 136.6, 134.4, 129.0, 128.9, 128.3, 121.6, 118.8, 113.8, 110.2, 79.3, 79.0, 78.1, 77.4, 77.0, 76.7, 76.2, 75.0, 56.6, 56.1, 56.0, 45.5; HRMS (ESI) (M+Na)^+^ Calcd for C_24_H_22_ClNO_4_Na^+^ 446.1130, found 446.1132.

**7o:** R_2_ = *p*-Cl, R_1_ = allyl, R_3_ = allyl, white solid, m.p.: 86–88°C; ^1^H NMR (400 MHz, CDCl_3_) *δ* 3.27–3.32 (m, 1H, NHC**H**_2_), 3.73–4.05 (m, 6H, OC**H**_3_+CHOC**H**_2_+NHC**H**_2_), 4.47–4.63 (m, 3H, OC**H**+ArOC**H**_2_), 5.16–5.42 (m, 4H, CHOCH_2_=CHC**H**_2_+ArOCH_2_=CHC**H**_2_), 5.84–6.09 (m, 3H, CHOCH_2_C**H**+ArOCH_2_C**H** +N**H**), 6.26 (d, 1H, *J* = 16.0 Hz, COC**H**), 6.84 (d, 1H, *J* = 8.0 Hz, Ar-**H**), 7.03–7.05 (m, 2H, Ar-**H**), 7.25–7.34 (m, 4H, Ar-**H**), 7.55 (d, 1H, *J* = 16.0Hz, Ar-C**H**);^13^C NMR (101 MHz, CDCl_3_) δ 166.0, 149.6, 149.5, 141.3, 137.9, 134.3, 134.0, 132.8, 128.9, 128.1, 127.9, 121.9, 118.4, 118.3, 117.5, 112.9, 110.0, 79.4, 69.8, 69.8, 56.0, 45.7; HRMS (ESI) (M+Na)^+^ Calcd for C_24_H_26_ClNO_4_Na^+^ 450.1443, found 450.1449.

**7p:** R_2_ = *p*-Cl, R_1_ = benzyl, R_3_ = benzyl, white solid, m.p.:131–132°C; ^1^H NMR (400 MHz, CDCl_3_) *δ* 3.29–3.35 (m, 1H, NHC**H**_2_), 3.83–3.92 (m, 4H, OC**H**_3_+NHC**H**_2_), 4.30 (d, 1H, *J* = 11.2 Hz, CHOC**H**_2_), 4.49–4.54 (m, 2H, CHOC**H**_2_+OC**H**), 5.19 (s, 2H, ArOC**H**_2_), 5.96 (br, 1H, N**H**), 6.20 (d, 1H, *J* = 16.0 Hz, COC**H**), 6.86 (d, 1H, *J* = 8.8 Hz, Ar-**H**), 7.00–7.04 (m, 2H, Ar-**H**), 7.30–7.44 (m, 14H, Ar-**H**), 7.52 (d, 1H, *J* = 16.0 Hz, Ar-C**H**); ^13^C NMR (101 MHz, CDCl_3_) δ 166.0, 149.8, 149.7, 141.2, 137.8, 137.8, 136.7, 134.0, 129.0, 128.7, 128.6, 128.2, 128.1, 128.0, 128.0, 127.9, 127.2, 121.9, 118.4, 113.5, 110.2, 79.6, 71.0, 70.9, 56.0, 45.7; HRMS (ESI) (M+Na)^+^ Calcd for C_32_H_30_ClNO_4_Na^+^ 550.1756, found 550.1759.

#### General procedure for the preparation of the compound 8

To a 500 mL tube-shaped flask, equipped with a magnetic stirrer, hydrogen balloon and a vacuum outlet was added 10% Pd/C (1 g). The flask was evacuated for 5 min at 0.001 mm Hg and flushed with H_2_. The evacuation cycle was repeated four times before the catalyst was suspended in EtOH (170 mL) and ferulic acid 1 (20 g, 0.1 mol) and concentrated hydrochloric acid (10 mL) were added. The resulting mixture was then stirred under a hydrogen atmosphere at 40°C for 20 h. Upon completion (monitored by TLC by the disappearance of the starting material), the solution was filtered through celite and the filter washed with EtOH (3 × 30 mL). The combined filtrates were then distilled *in vacuo* and the resulting residue dried at 20–23°C for 1 h at 0.01 mm Hg to give compound 8 (21.95 g).

#### Preparation of the compound 9

Substituted 2-amino-1-phenylethanol (3.6 g, 21 mmol) and DMAP (0.244 g, 2 mmol) were added to compound 9 (4.5 g, 20 mmol) in a microwave vial. The vial was then sealed and irradiated in a microwave reactor at 130°C (10 bar) for 30 min. The reaction mixture was then diluted with water and extracted several times with EtOAc. The combined organic phases were then washed with brine, dried over MgSO_4_ and evaporated to dryness to give the crude product as a residue, which was purified by chromatography on silica gel using petroleum ether (60–90%) and ethyl acetate as the eluent to afford a pale purple solid 9, yield: 60%, m.p.: 108–110°C. ^1^H NMR (400 MHz, CDCl_3_) *δ* 2.40 (t, 2H, *J* = 7.2 Hz, COCH_2_), 2.83 (t, 2H, *J* = 6.8 Hz, Ar-C**H**_2_), 3.15–3.21 (m, 1H, NHC**H**_2_),3.54–3.58 (m, 1H, NHC**H**_2_), 3.80 (s, 3H, OC**H**_3_), 4.68–4.69 (m, 1H, OC**H**), 5.65 (br, 1H, N**H**), 6.60–6.79 (m, 3H, Ar-**H**), 7.13–7.24 (m, 4H, Ar-**H**).

#### Preparation of the compound 10a-10b

Compound 10 was prepared according to the same procedure as compound 6.

**10a:** R_2_ = Cl, R_1_ = *n*-Pr, white solid, m.p.: 100–102°C; ^1^H NMR (400 MHz, CDCl_3_) *δ* 1.02 (t, 3H, *J =* 7.2 Hz, CH_2_C**H**_3_), 1.80–1.88 (m, 2H, CH_3_C**H**_2_), 2.46 (t, 2H, *J =* 7.2 Hz, COC**H**_2_), 2.89 (t, 2H, *J =* 7.2 Hz, Ar-C**H**_2_), 3.58–3.63 (m, 2H, NHC**H**_2_), 3.84 (s, 3H, OC**H**_3_), 3.94 (t, 2H, *J =* 6.8 Hz, ArOC**H**_2_), 4.73–4.74 (m, 1H, OC**H**), 5.75 (br, 1H, N**H**), 6.68–6.80 (m, 3H, Ar-**H**), 7.20–7.29 (m, 4H, Ar-**H**).

**10b:** R_2_ = H, R_1_ = Me, yellow solid, m.p.: 94–95°C; ^1^H NMR (400 MHz, CDCl_3_) *δ* 2.50 (t, 2H, *J* = 7.2 Hz, CH_2_C**H**_2_), 2.94 (t, 2H, *J* = 7.2 Hz, CH_2_C**H**_2_), 3.28–3.35 (m, 1H, CHC**H**_2_), 3.65–3.72 (m, 1H, CHC**H**_2_), 3.88 (s, 3H, *o*-ArOC**H**_3_), 3.89 (s, 3H, *p*-ArOC**H**_3_), 4.80 (dd, 1H, *J* = 2.8 Hz, CH_2_C**H**), 5.85 (br, 1H, CON**H**), 6.74–6.83 (m, 3H, Ar-**H**), 7.31–7.36 (m, 5H, Ar-**H**).

#### Preparation of the compound 12

To a 500-mL tube-shaped flask, equipped with a magnetic stirrer, hydrogen balloon and a vacuum outlet was added 10% Pd/C (1.5 g). The flask was evacuated for 5 min at 0.001 mm Hg and flushed with H_2_. This evacuation cycle was repeated four times before the catalyst was suspended in a mixture of EtOH (150 mL), EtOAc (150 mL) and (3,4-dimethoxyphenyl)acrylic acid 1 (15 g, 72 mmol). The resulting mixture was then stirred under a hydrogen atmosphere at 40°C for 72 h. Upon completion of the reaction, the solution was filtered through celite and the celite plug washed with EtOH (3 × 30 mL). The combined filtrates were then distilled *in vacuo* to give the corresponding product 12 as a white solid, m.p.; 92–93°C.

#### Preparation of the compound 10c

Isobutyl chloroformate (6.84 mmol) was added to a solution of 12 (6.84 mmol) and NMM (7.18 mmol) in anhydrous THF (15 mL) at −20°C. The resulting mixture was agitated for 30 min and the amine (1.05 mmol) was then added drop-wise. The mixture was then stirred for 1 h at −10°C followed by 12 h at room temperature. The reaction mixture was distilled *in vacuo* and the resulting solid dissolved in DCM (40 mL), before being washed with brine, dried over MgSO_4_, and evaporated *in vacuo* to give the crude product, which was purified by flash chromatography eluting with petroleum ether-EtOAc (1: 3) to give compound 10c as a white solid; Yield: 73%, m.p.: 102–104°C; ^1^H NMR (400 MHz, CDCl_3_) *δ* 2.47 (t, 2H, *J* = 7.6 Hz, CH_2_CH_2_), 2.90 (t, 2H, *J* = 7.6 Hz, CH_2_C**H**_2_), 3.21–3.28 (m, 1H, CHCH_2_), 3.58–3.64(m, 1H, CHC**H**_2_), 3.85(s, 3H, *p*-ArOC**H**_3_), 3.86 (s, 3H, *o*-ArOC**H**_3_), 4.75(dd, 1H, *J* = 3.5 Hz, CH_2_C**H**), 5.81(br, 1H, CON**H**), 6.71–6.80 (m, 3H, Ar-H), 7.21 (d, 2H, *J* = 8.4 Hz, Ar-**H**), 7.29 (d, 2H, *J* = 8.4 Hz, Ar-**H**).

#### Preparation of the compound 13

Compound 13 was prepared according to the same procedure as compound 7.

**13a:** R_2_ = H, R_1_ = Me, R_3_ = benzyl, white solid, m.p.: 131–132°C; Yield: 70%; ^1^H NMR (400 MHz, CDCl_3_) *δ* 2.44 (t, 2H, *J* = 7.2 Hz, CH_2_C**H**_2_), 2.90 (t, 2H, *J* = 7.2 Hz, CH_2_C**H**_2_), 3.22–3.27 (m, 1H, CH_2_C**H**), 3.73–3.78 (m, 1H, CH_2_C**H**), 3.86 (s, 3H, *o*-ArOC**H**_3_), 3.88 (s, 3H, *p*-ArOC**H**_3_), 4.24 (d, 1H, *J* = 11.2 Hz, PhC**H**_2_), 4.42 (dd, 1H, *J* = 4.0 Hz, CH_2_C**H**), 4.50 (d, 1H, *J* = 11.2 Hz, PhC**H**_2_), 5.78 (br, 1H, CON**H**), 6.75–6.80 (m, 3H, Ar-**H**), 7.31–7.42(m, 10H, Ar-**H**); ^13^C NMR (101 MHz, CDCl_3_) δ 172.0, 148.9, 147.4, 139.2, 138.1, 133.5, 128.7, 128.5, 128.3, 127.9, 126.8, 120.1, 111.7, 111.3, 80.2, 70.7, 55.9, 55.8, 45.6, 38.8, 31.3; HRMS (ESI) (M+Na)^+^ Calcd for C_26_H_29_NO_4_Na^+^ 442.1983, found 442.1989.

**13b:** R_2_ = *p*-Cl, R_1_ = Me, R_3_ = propynyl, white solid, m.p.: 78–80°C; Yield: 71%; ^1^H NMR (400 MHz, CDCl_3_) *δ* 2.44 (s, 1H, C≡C**H**), 2.50 (t, 2H, *J* = 7.6 Hz, CH_2_C**H**_2_),. 2.94 (t, 2H, *J* = 7.6 Hz, CH_2_C**H**_2_), 3.13–3.18 (m, 1H, CH_2_C**H**), 3.71–3.78 (m, 1H, CH_2_C**H**), 3.84 (s, 1H, C≡CHC**H**_2_), 3.88 (s, 3H, *o*-ArOC**H**_3_), 3.89 (s, 3H, *p*-ArOC**H**_3_), 4.12 (dd, 1H, *J* = 15.6 Hz, *J* = 2.0 Hz, C≡CHC**H**_2_), 4.54 (dd, 1H, *J* = 9.2 Hz, *J* = 3.6 Hz, CH_2_C**H**Ar), 5.86 (br, 1H, CON**H**), 6.76–6.83 (m, 3H, Ar-**H**), 7.24 (d, 2H, *J* = 8.0 Hz, Ar-**H**), 7.35 (d, 2H, *J* = 8.0 Hz, Ar-**H**); HRMS (ESI) (M+Na)^+^ Calcd for C_22_H_24_ClNO_4_Na^+^ 424.1292, found 424.1290.

**13c:** R_2_ = *p*-Cl, R_1_ = Me, R_3_ = benzyl, white solid, m.p.: 91°C; Yield: 65%; ^1^H NMR (400 MHz, CDCl_3_) *δ* 2.41 (t, 2H, *J* = 7.6 Hz, CH_2_C**H**_2_), 2.87 (t, 2H, CH_2_**CH**_2_), 3.13–3.21 (m, 1H, C**H**_2_CH), 3.65–3.72 (m, 1H, C**H**_2_CH), 3.84 (s, 3H, *o*-ArOC**H**_3_), 3.86 (s, 3H, *p*-ArOC**H**_3_), 4.21 (d, 1H, *J* = 11.6 Hz, PhC**H**_2_), 4.38 (dd, 1H, *J* = 4.0 Hz, CH_2_C**H**), 4.45 (d, 1H, *J* = 11.6 Hz, PhC**H**_2_), 5.74 (br, 1H, CON**H**), 6.72–6.79 (m, 3H, Ar-**H**), 6.92–6.94 (m, 2H, Ar-**H**), 7.25–7.36 (m, 7H, Ar-**H**); ^13^C NMR (101 MHz, CDCl_3_) δ 172.0, 148.9, 147.5, 137.8, 134.0, 133.4, 128.9, 128.6, 128.1, 128.0, 127.9, 120.1, 111.7, 111.3, 79.5, 70.8, 55.9, 55.8, 45.5, 38.7, 31.3; HRMS (ESI) (M+Na)^+^ Calcd for C_26_H_29_NO_4_Na^+^ 476.1590, found 476.1599.

**13d:** R_2_ = *p*-Cl, R_1_ = *n*-Pr, R_3_ = Me, white solid; m.p.: 92–93°C; Yield: 65%; ^1^H NMR (400 MHz, CDCl_3_) *δ* 1.01 (t, 3H, *J =* 6.8Hz, CH_2_C**H**_3_), 1.80–1.87 (m, 2H, CH_3_C**H**_2_), 2.47 (t, 2H, *J =* 6.8 Hz, COC**H**_2_), 2.90 (t, 2H, *J =* 6.8 Hz, Ar–C**H**_2_), 3.08-3.17 (m, 4H, CHOC**H**_3_+NHC**H**_2_), 3.61–3.66 (m, 1H, NHC**H**_2_), 3.85 (s, 3H, ArOC**H**_3_), 3.94 (t, 2H, *J =* 6.4 Hz, ArOC**H**_2_), 4.13 (dd, 1H, *J =* 5.2 Hz, *J =* 7.6 Hz, OC**H**), 5.81 (br, 1H, CON**H**), 6.70–6.84 (m, 3H, Ar-**H**), 7.17–7.33 (m, 4H, Ar-**H**); ^13^C NMR (101 MHz, CDCl_3_) δ 172.1, 149.3, 147.0, 137.6, 133.9, 133.4, 128.8, 128.0, 120.2, 113.1, 112.2, 81.7, 70.6, 56.8, 56.0, 45.5, 38.7, 31.3, 22.5, 10.5; HRMS (ESI) (M+H)^+^ Calcd for C_22_H_28_ClNO_4_H^+^ 406.1770, found 406.1780.

**13e:** R_2_ = *p*-Cl, R_1_ = *n*-Pr, R_3_ = Et, white solid; m.p.: 69–70°C; Yield: 59%;^1^H NMR (400 MHz, CDCl_3_) *δ* 1.01 (t, 3H, *J =* 5.2Hz, CHOCH_2_C**H**_3_), 1.13 (t, 3H, *J =* 5.2Hz, ArOCH_2_CH_2_C**H**_3_), 1.78–1.92 (m, 2H, ArOCH_2_C**H**_2_), 2.48 (t, 2H, *J =* 6.0 Hz, COC**H**_2_), 2.90 (t, 2H, *J =* 6.0 Hz, Ar-C**H**_2_), 3.08–3.68 (m, 3H, CHOC**H**_2_+NHC**H**_2_), 3.60–3.64 (m, 1H, NHC**H**_2_), 3.84 (s, 3H, ArOC**H**_3_), 3.93 (t, 2H, *J =* 6.4 Hz, ArOC**H**_2_), 4.23 (dd, 1H, *J =* 2.8 Hz, *J =* 4.8 Hz, OC**H**), 5.82 (br, 1H, CON**H**), 6.68–6.84 (m, 3H, Ar-**H**), 7.18–7.31 (m, 4H, Ar-**H**); ^13^C NMR (101 MHz, CDCl_3_) δ 172.1, 149.3, 147.0, 138.3, 133.7, 133.4, 128.7, 127.9, 120.2, 113.0, 112.2, 79.8, 70.6, 64.5, 56.0, 45.5, 38.7, 31.3, 22.5, 15.2, 10.5; HRMS (ESI) (M+H)^+^ Calcd for C_23_H_30_ClNO_4_H^+^ 420.1933, found 420.1936.

**13f:** R_2_ = *p*-Cl, R_1_ = *n*-Pr, R_3_ = propynyl, white solid; m.p.: 61–62°C; Yield: 66%; ^1^H NMR (400 MHz, CDCl_3_) *δ* 1.02 (t, 3H, *J* = 7.2 Hz, CH_2_C**H**_3_), 1.80–1.87 (m, 2H, CH_3_C**H**_2_), 2.41 (s, 1H, C≡C**H**), 2.48 (t, 2H, *J* = 7.2 Hz, COC**H**_2_), 2.90 (t, 2H, *J* = 7.6 Hz, Ar-C**H**_2_), 3.10–3.16 (m, 1H, NHC**H**_2_), 3.69–3.74 (m, 1H, NHC**H**_2_), 3.81–3.85 (m, 4H, OC**H**_3_ + CHOC**H**_2_), 3.94 (t, 2H, *J* = 6.8 Hz, ArOC**H**_2_), 4.10 (d, 1H, *J* = 16.0 Hz, CHOC**H**_2_), 4.51 (dd, 1H, *J* = 3.6 Hz, *J* = 8.4 Hz, OC**H**), 5.87 (br, 1H, N**H**), 6.71–6.84 (m, 3H, Ar-**H**), 7.21–7.34 (m, 4H, Ar-**H**); ^13^C NMR (101 MHz, CDCl_3_) δ 172.1, 149.6, 145.2, 136.6, 135.0, 134.3, 128.9, 128.2, 120.1, 114.6, 112.2, 79.3, 78.9, 78.8, 75.8, 74.9, 56.8, 56.1, 55.9, 45.2, 38.5, 31.3. HRMS (ESI) (M+H)^+^ Calcd for C_24_H_28_ClNO_4_H^+^ 430.1781, found 430.1780.

**13g:** R_2_ = *p*-Cl, R_1_ = *n*-Pr, R_3_ = allyl, white solid; m.p.: 56–58°C; Yield: 72%; ^1^H NMR (400 MHz, CDCl_3_) *δ* 1.02 (t, 3H, *J =* 7.2 Hz, CH_2_C**H**_3_), 1.80–1.87 (m, 2H, CH_3_C**H**_2_), 2.47 (t, 2H, *J =* 6.8 Hz, COC**H**_2_), 2.89 (t, 2H, *J =* 7.2 Hz, Ar-C**H**_2_), 3.13–3.17 (m, 1H, NHC**H**_2_), 3.63–3.73 (m, 2H, NHC**H**_2_+ CHOC**H**_2_), 3.82–3.95 (m, 6H, OC**H**_3_+ArOC**H**_2_+CHOC**H**_2_), 4.32 (dd, 1H, *J =* 2.8 Hz, *J =* 6.0 Hz, OC**H**), 5.15–5.22 (m, 2H, CH=C**H**_2_), 5.81–5.85 (m, 2H, CH_2_=CH+NH), 6.70–6.84 (m, 3H, Ar-**H**), 7.18–7.32 (m, 4H, Ar-**H**); ^13^C NMR (101 MHz, CDCl_3_) δ 172.1, 149.3, 147.0, 137.9, 134.2, 133.9, 133.4, 128.8, 128.0, 120.2, 117.4, 113.1, 112.2, 79.3, 70.6, 69.8, 56.0, 45.5, 38.7, 31.3, 22.5, 10.5; HRMS (ESI) (M+H)^+^ Calcd for C_24_H_30_ClNO_4_H^+^ 432.1931, found 432.1936.

**13h:** R_2_ = *p*-Cl, R_1_ = *n*-Pr, R_3_ = benzyl, white solid; m.p.: 79–81°C; Yield: 70%; ^1^H NMR (400 MHz, CDCl_3_) *δ* 1.02 (t, 3H, *J* = 7.2 Hz, CH_2_C**H**_3_), 1.80–1.87 (m, 2H, CH_3_C**H**_2_), 2.47 (t, 2H, *J* = 6.8 Hz, COC**H**_2_), 2.89 (t, 2H, *J* = 7.2 Hz, Ar-C**H**_2_), 3.13–3.17 (m, 1H, NHC**H**_2_), 3.63–3.73 (m, 2H, NHC**H**_2_+CHOC**H**_2_), 3.82–3.95 (m, 6H, OC**H**_3_+ArOC**H**_2_+CHOC**H**_2_), 4.32 (dd, 1H, *J* = 2.8 Hz, *J* = 6.0 Hz, OC**H**), 5.15–5.22 (m, 2H, CH=C**H**_2_), 5.81–5.85 (m, 2H, CH_2_=C**H**+N**H**), 6.70–6.84 (m, 3H, Ar-**H**), 7.18–7.32 (m, 4H, Ar-**H**); ^13^C NMR (101 MHz, CDCl_3_) δ 172.1, 149.4, 147.0, 137.8, 137.8, 134.0, 133.4, 128.9, 128.6, 128.2, 127.98, 127.9, 120.2, 113.1, 112.2, 79.5, 70.9, 70.6, 56.0, 45.5, 38.7, 31.3, 22.5, 10.5; HRMS (ESI) (M+H)^+^ Calcd. for C_28_H_32_ClNO_4_H^+^ 482.2092, found 482.2093.

**13i:** R_2_ = *p*-Cl, R_1_ = propynyl, R_3_ = propynyl, white solid; m.p.: 89–90°C; Yield: 41%; ^1^H NMR (400 MHz, CDCl_3_) *δ* 2.41–2.48 (m, 4H, COC**H**_2_+ArOCH_2_C≡C**H**+CHOCH_2_C≡C**H**), 2.91 (t, 2H, *J* = 6.8Hz, Ar-C**H**_2_), 3.10–3.16 (m, 1H, NHC**H**_2_), 3.69–3.73 (m, 1H, NHC**H**_2_), 3.81–3.85 (m, 4H, ArOC**H**_3_+CHOC**H**_2_), 4.10 (d, 1H, *J =* 16.0Hz, CHOC**H**_2_), 4.51 (dd, 1H, *J =* 2.8Hz, *J =* 7.6Hz, OC**H**), 4.73 (s, 2H, ArOC**H**_2_), 5.87 (br, 1H, N**H**), 6.73–6.79 (m, 2H, Ar-**H**), 6.95 (d, 1H, *J =* 8.0Hz, Ar-**H**), 7.21–7.33 (m, 4H, Ar-**H**); ^13^C NMR (101 MHz, CDCl_3_) δ 172.2, 149.3, 147.0, 136.7, 134.3, 133.4, 128.9, 128.2, 120.2, 113.1, 112.2, 79.3, 78.9, 74.9, 70.6, 56.0, 56.0, 45.2, 38.7, 31.3, 22.5, 10.5; HRMS (ESI) (M+H)^+^ Calcd for C_24_H_24_ClNO_4_H^+^ 426.1458, found 426.1467.

## Biological assay

### Antiviral biological assay

#### Purification of tobacco mosaic virus

Using Gooding’s method [27], the upper leaves of *Nicotiana tabacum L.* inoculated with TMV were selected and ground in a phosphate buffer before being filtered through a double-layer pledget. The filtrate was centrifuged at 10000 g, treated twice with PEG, and centrifuged again. The entire experiment was conducted at 4°C. The absorbance values were estimated at 260 nm using an ultraviolet spectrophotometer according to the following equation:

$$\mathrm{Virus}\;\mathrm{concentration}\;=\left(A_{260}\times \mathrm{dilution}\;\mathrm{ration}\right)/E_{1\mathrm{cm}}^{0.1\%,260\mathrm{nm}}$$

#### Protective effect of compounds against TMV in vivo

The compound solution was smeared on the left side of growing *N. tabacum L.* leaves of the same ages, whereas the solvent alone was used as a control on the right side. The leaves were then inoculated with the virus after 12 h. A brush was dipped into tobacco mosaic virus of 6 × 10^-3^ mg/mL to inoculate the leaves, which were previously scattered with silicon carbide. The leaves were then washed with water and rubbed softly once or twice along the nervature. The local lesion numbers appearing 3–4 days after inoculation were counted. The experiment was performed in triplicate for each compound.

#### Curative effect of compounds against TMV in vivo

Growing leaves of *N. tabacum L.* of the same ages were selected. The tobacco mosaic virus (concentration of 6 × 10^-3^ mg/mL) was dipped and inoculated onto the whole leaves. The leaves were then washed with water and dried. The compound solution was then smeared onto the left sides of the leaves, whereas the solvent alone was used as a control and smeared on the right side. The local lesion numbers were then counted and recorded 3–4 days after the inoculation [25]. The experiment was performed in triplicate for each compound. The *in vivo* inhibition rate of each compound was then calculated according to the following formula:

$$\begin{array}{l} \mathrm{Inhibition}\;\mathrm{rate}\left(\%\right)\\ =\left[\left(\mathrm{av}\;\mathrm{local}\;\mathrm{lesion}\;\mathrm{no}.\mathrm{of}\;\mathrm{control}-\mathrm{av}\;\mathrm{local}\;\mathrm{lesion}\;\mathrm{no}.\mathrm{of}\;\mathrm{drug-treated}\right)/\mathrm{av}\;\mathrm{local}\;\mathrm{lesion}\;\mathrm{no}.\mathrm{of}\;\mathrm{control}\right]\times 100\% \end{array}$$

Where “av” means average, and the controls were not treated with any test compounds.

### Larvicidal activity against mosquitoes

A stock solution of each compound was prepared at 1000 μg/mL using acetone as a solvent. Each compound solution in acetone was then suspended in distilled water with Tween-80 (0.001%). Distilled water mixed with Tween-80 was used as a control. Batches of 10 fourth-stage larvae of *C. pipiens pallens* were pipetted into beakers (100 mL) containing each of test solution (40 mL). Each test compound was evaluated at a 2 μg/mL level in distilled water. Observations of larval mortality were recorded after 24 h. The larvae were considered dead if appendages did not move when prodded with a needle.

$$\begin{array}{l} \mathrm{Rectified}\;\mathrm{mortality}\left(\%\right)=(A1-A2)/(100-A2)\\ \;\times 100 \end{array}$$

where Al (%) is the mortality in the treatment group, and A2 (%) is the mortality in the control group.

### Insecticidal activity against bean aphids (Aphis fabae Scopoli)

Bean aphids were dipped according to a slightly modified FAO (Food and Agriculture Organization) dip test [28]. The tender shoots of soybean with 40–60 healthy apterous adult aphids were dipped in the diluted compound solutions for 5 s, and the superfluous fluid was removed and placed in a conditioned room. Mortality was calculated 48 h after treatment. Each treatment was performed in triplicate. Chlorfenapyr was used as a standard. The data for the mortality-regression lines of the compounds were subjected to probit analysis by Finney’s method.

### Acaricidal activity against two spot spider mite (T. cinnabarinus boisduval)

Thirty female adult spider mites were fixed dorsally to a strip of double-sided tape attached to the slide using a small brush. The slide was immersed and shaken for 10 s in a diluted solution of the test compound. After the excessive solution was removed, the treated slides with the mites were kept at 25°C (± 2°C) in a Petri dish with moist filter paper. The number of the dead mites was recorded 24 h after treatment. Each treatment was performed in triplicate, with each experiment involving 30 adult mites [29]. Chlorfenapyr was used as the standard. The data for the mortality-regression lines of the compounds were subjected to probit analysis by Finney’s method.

## Conclusion

In conclusion, two series of ferulic acid derivatives have been synthesized efficiently. The bioassay showed title compounds not only inhibit the plant viral infection, but also prevented the spread of plant virus by insect vectors. Compounds 7a, 7b and 7d in particular exhibited good levels of antiviral and insecticidal activity. These findings therefore demonstrate that the ferulic acid amides represent a new template for future antiviral studies.

## Competing interests

The authors declare that they have no competing interests.

## Authors’ contributions

The current study is an outcome of constructive discussion with ZML and WGZ who offered necessary guidance to GYH and CC to carry out their synthesis and characterization experiments. WGZ were also involved in the drafting of the manuscript. YQL, LXX, LZW and SJY performed the biological activity tests; GYH, CC and ZPW carried out the ^1^H NMR and HRMS. All authors read and approved the final manuscript.

## Supplementary Material

Additional file 1 **Copies of **^**1**^**H NMR, **^**13**^**C NMR and HRMS.** Click here for file
