# Supplementary material for: Synthesis and characteristics of (Hydrogenated) ferulic acid derivatives as potential antiviral agents with insecticidal activity
Source: Chem Cent J. 2013 Feb 14;7:33. doi: 10.1186/1752-153X-7-33 (PMC3599306; doi:10.1186/1752-153X-7-33)

# Additional file 1

## Design, Synthesis, Antiviral and Insecticidal Evaluation of (Hydrogenated) Ferulic Acid Derivatives as Potential Antiviral Agents with Insecticidal Activity

Guang-Ying Huang, Can Cui, Zhi-Peng Wang, Yong-Qiang Li, Li-Xia Xiong, Li-Zhong Wang, Shu-Jing Yu, Zheng-Ming Li and Wei-Guang Zhao*

*State Key Laboratory of Elemento-Organic Chemistry, National Pesticide Engineering Research Center (Tianjin), Nankai University, Tianjin 300071, China*

**Author to whom correspondence should be addressed;**

***Corresponding author Wei-Guang Zhao** Fax: 00862223505948; Tel: 00862223498368;

E-mail: zwg@nankai.edu.cn

PDF created with pdfFactory trial version www.pdffactory.com

## Supporting Information

Table contents

Copies of $^{1}$H NMR and HRMS............................................................ S12

PDF created with pdfFactory trial version www.pdffactory.com

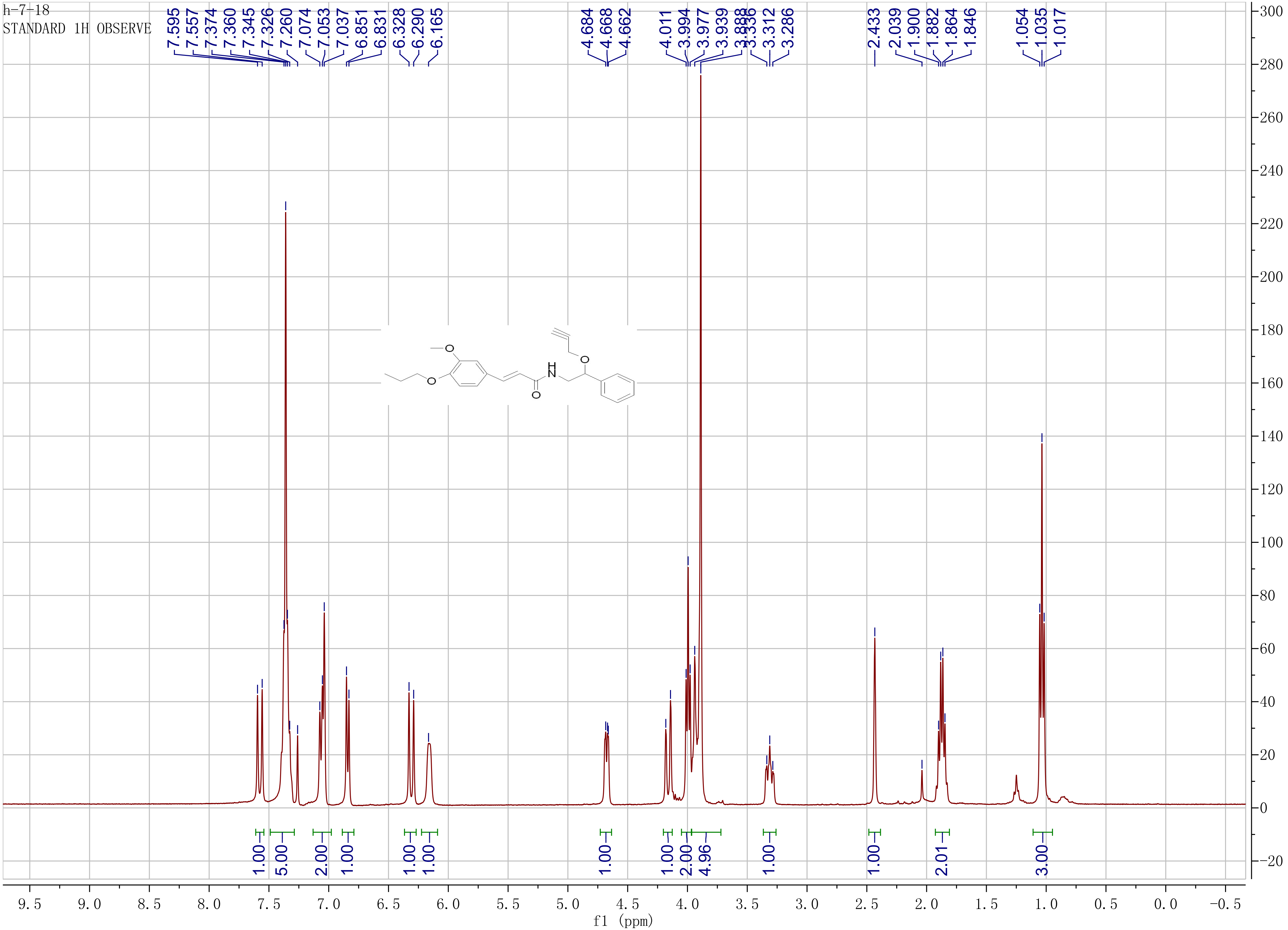
h-7-18
STANDARD 1H OBSERVE
7.595
7.557
7.374
7.360
7.345
7.326
7.260
7.074
7.053
7.037
6.851
6.831
6.328
6.290
6.165
4.684
4.668
4.662
4.011
3.994
3.977
3.939
3.312
3.286
2.433
2.039
1.900
1.882
1.864
1.846
1.054
1.035
1.017
1.00
5.00
2.00
1.00
1.00
1.00
1.00
1.00
2.00
4.96
1.00
1.00
2.01
3.00
f1 (ppm)

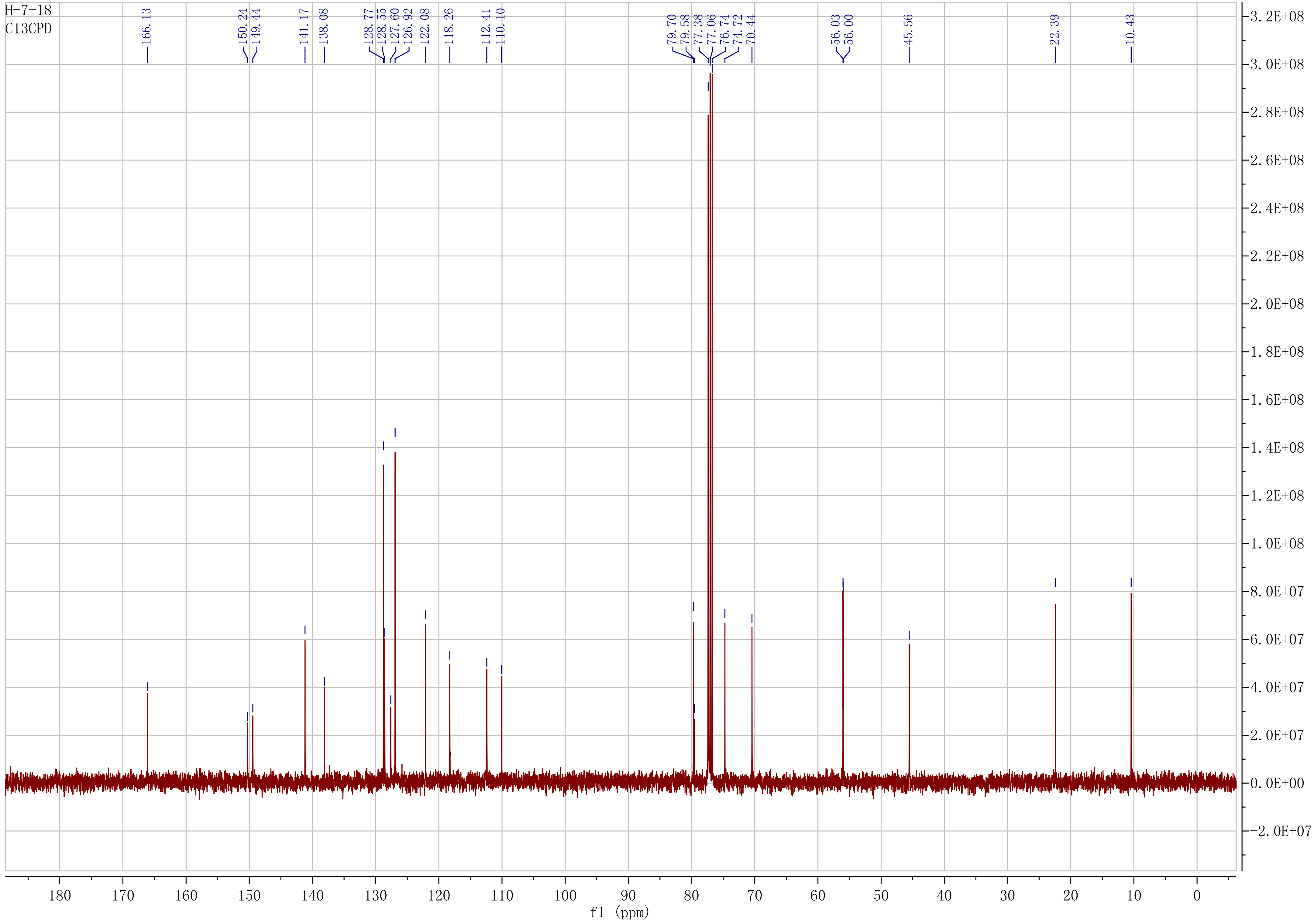

H-7-18
C13CPD
166.13
150.24
149.44
141.17
138.08
128.77
128.55
127.60
126.92
122.08
118.26
112.41
110.10
79.70
79.58
77.38
77.06
76.74
74.72
70.44
56.03
56.00
45.56
22.39
10.43
f1 (ppm)

Varian ProMALDI
File: h-7-18_MALDI.trans

Mode: Positive
Scans: 1

Date: 29-MAR-200
Time: 19:35:55
Scale: 222.5804

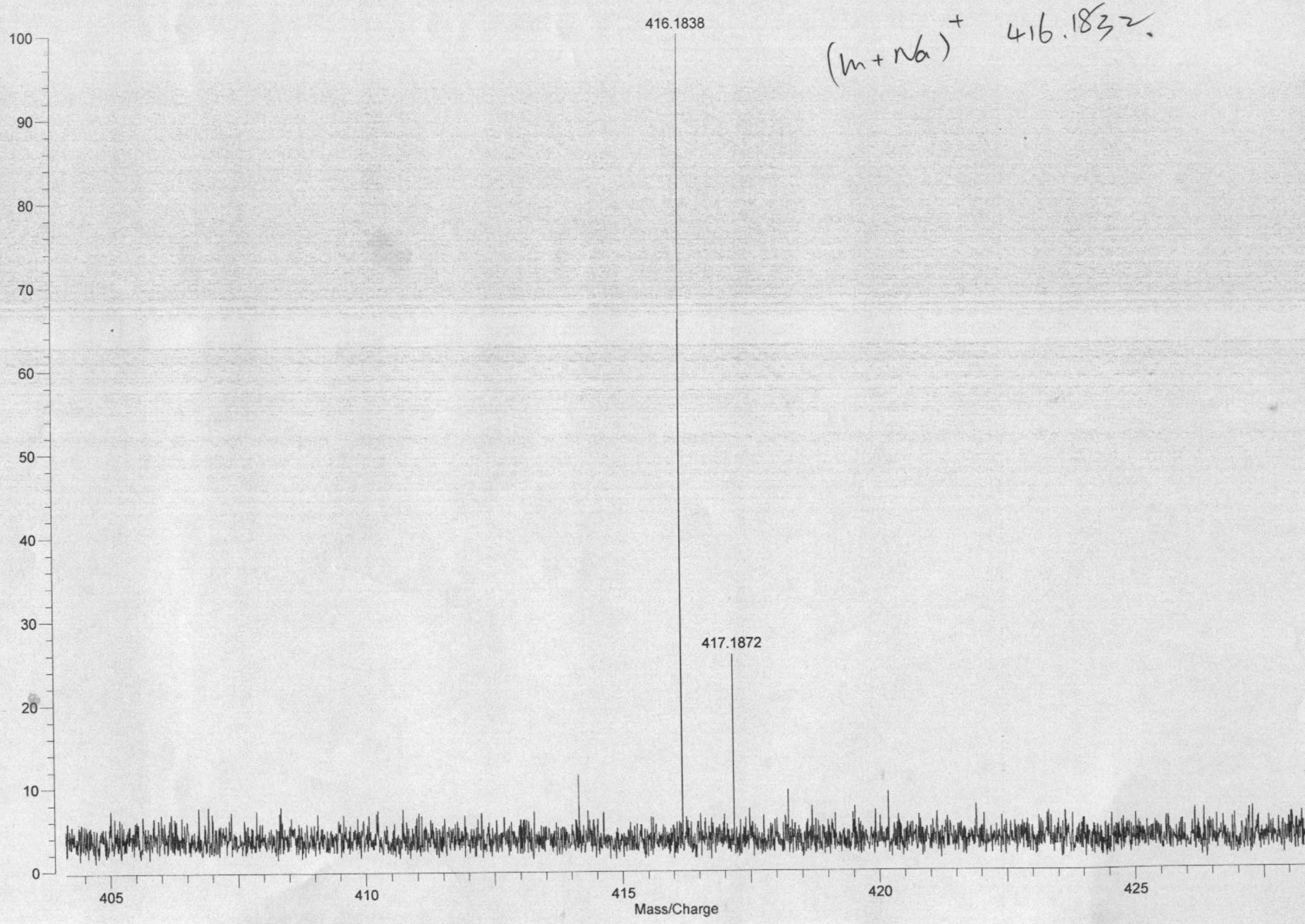

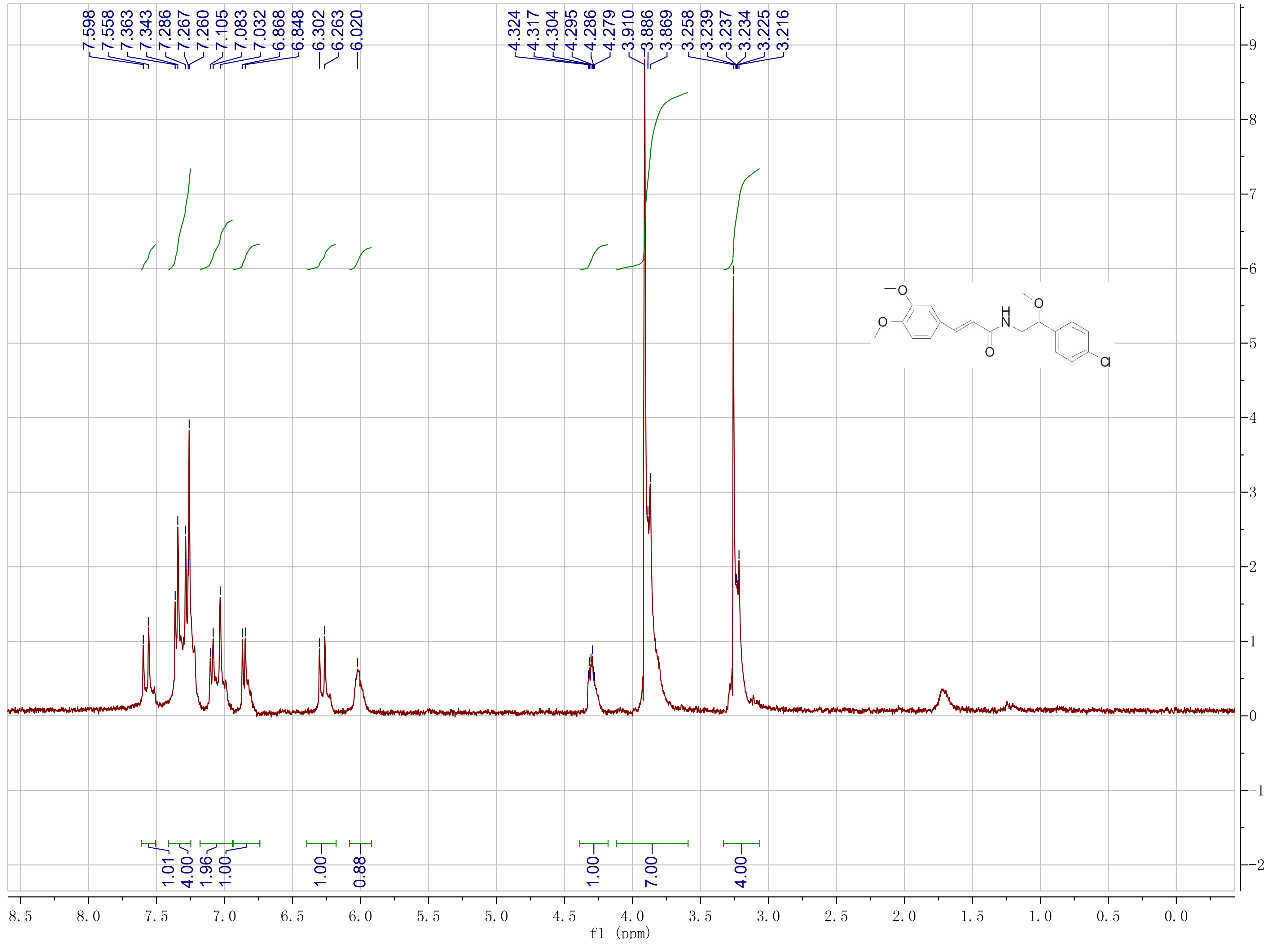
7.598
7.558
7.363
7.343
7.286
7.267
7.260
7.105
7.083
7.032
6.868
6.848
6.302
6.263
6.020
4.324
4.317
4.304
4.295
4.286
4.279
3.910
3.886
3.869
3.258
3.239
3.237
3.234
3.225
3.216
1.01
4.00
1.96
1.00
1.00
0.88
1.00
7.00
4.00
f1 (ppm)
O
O
H
N
O
O
Cl

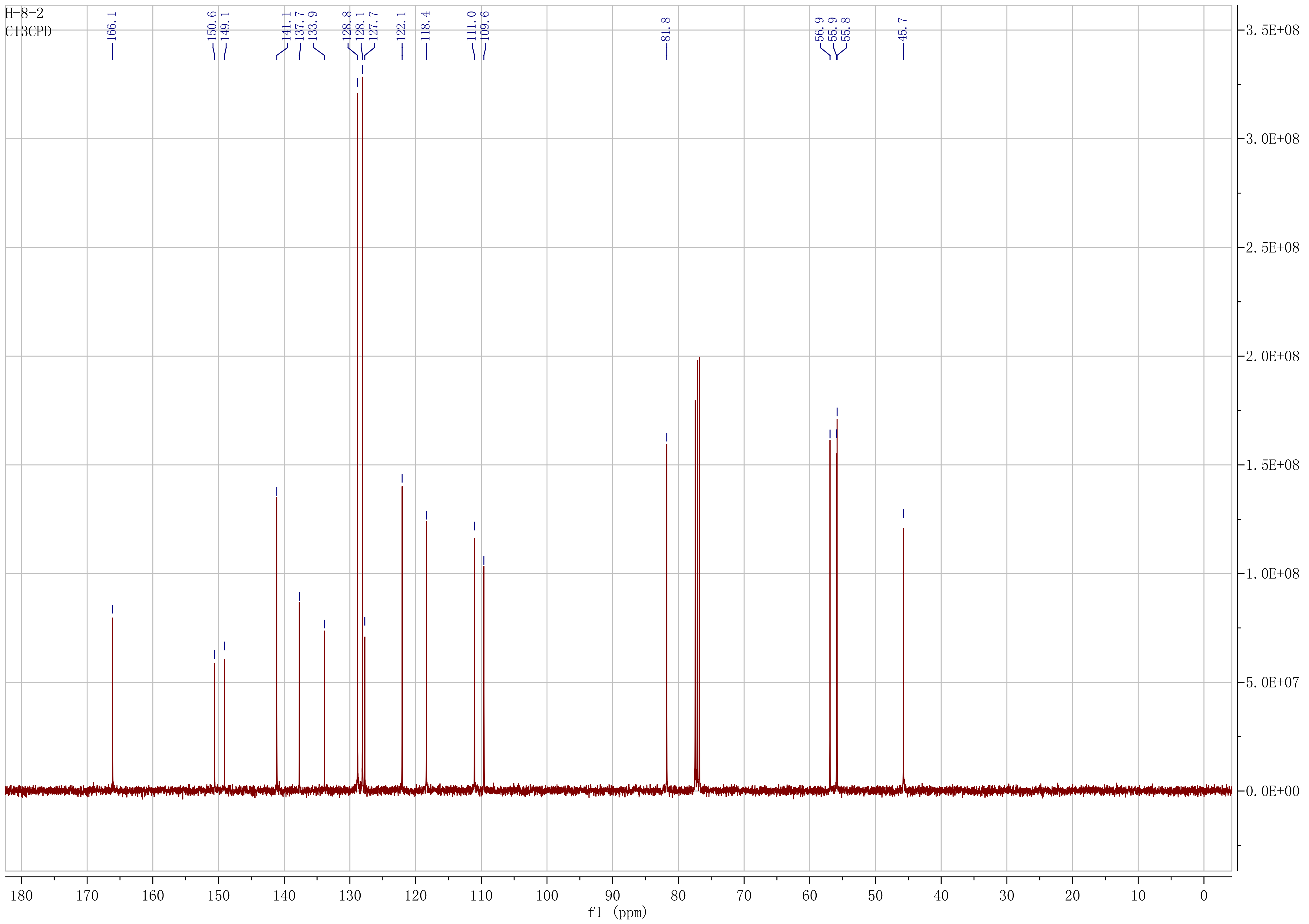
H-8-2
C13CPD
166.1
150.6
149.1
141.1
137.7
133.9
128.8
128.1
127.7
122.1
118.4
111.0
109.6
81.8
56.9
55.9
55.8
45.7
f1 (ppm)

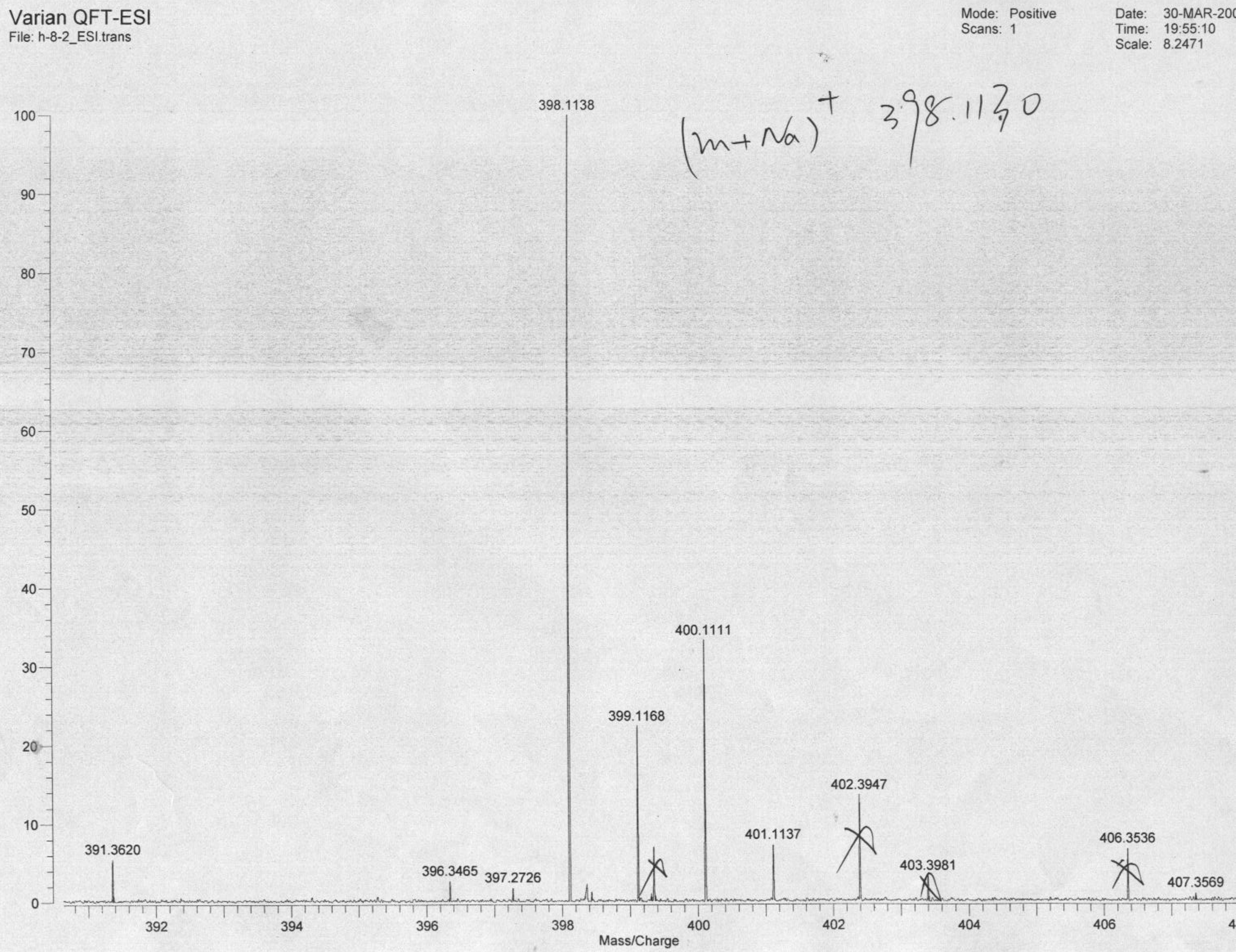

Varian QFT-ESI
File: h-8-2_ESI.trans
Mode: Positive
Scans: 1
Date: 30-MAR-200
Time: 19:55:10
Scale: 8.2471
(m+Na)+ 398.11?0
398.1138
400.1111
399.1168
402.3947
401.1137
406.3536
391.3620
403.3981
396.3465
397.2726
407.3569
Mass/Charge

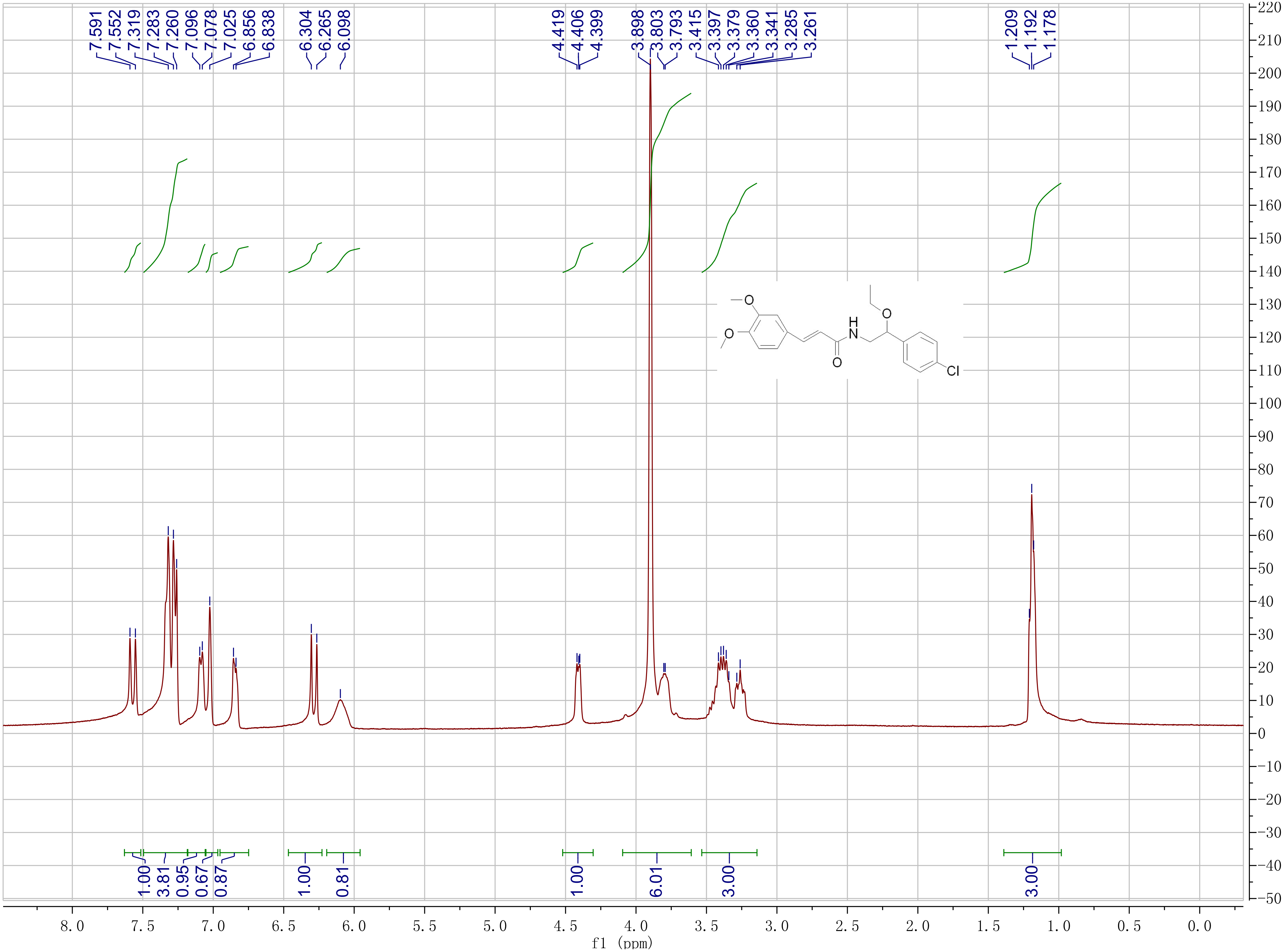
7.591
7.552
7.319
7.283
7.260
7.096
7.078
7.025
6.856
6.838
6.304
6.265
6.098
4.419
4.406
4.399
3.898
3.803
3.793
3.415
3.397
3.379
3.360
3.341
3.285
3.261
1.209
1.192
1.178
1.00
3.81
0.95
0.67
0.87
1.00
0.81
1.00
6.01
3.00
3.00
Cl
f1 (ppm)

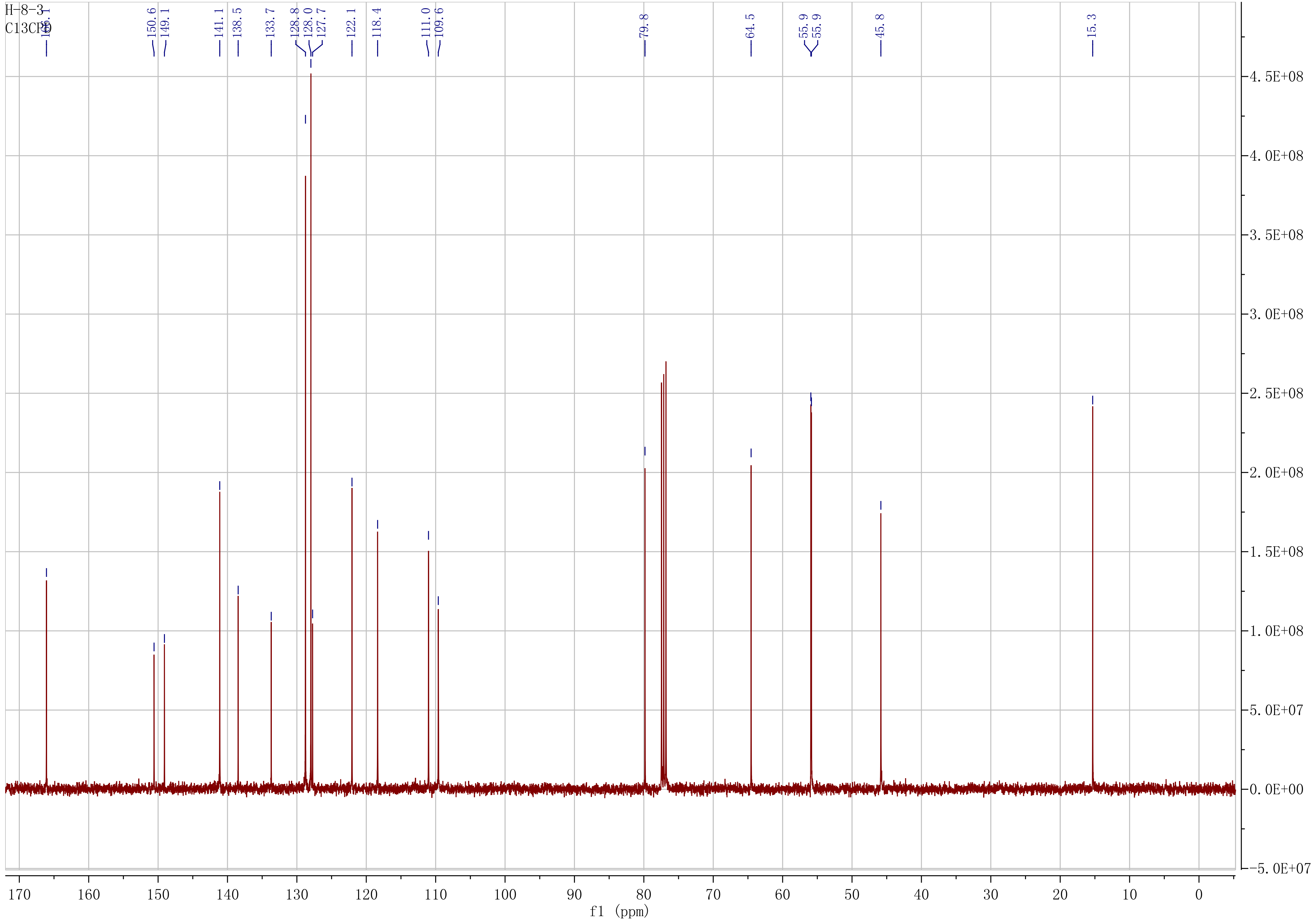
H-8-3
C13CPD
166.1
150.6
149.1
141.1
138.5
133.7
128.8
128.0
127.7
122.1
118.4
111.0
109.6
79.8
64.5
55.9
55.9
45.8
15.3
4.5E+08
4.0E+08
3.5E+08
3.0E+08
2.5E+08
2.0E+08
1.5E+08
1.0E+08
5.0E+07
0.0E+00
−5.0E+07
170
160
150
140
130
120
110
100
90
80
70
60
50
40
30
20
10
0
f1 (ppm)

Varian QFT-ESI
File: h-8-3_ESI.trans

Mode: Positive
Scans: 1

Date: 30-MAR-200
Time: 19:49:27
Scale: 80.7864

(m+Na)+ 412.1286

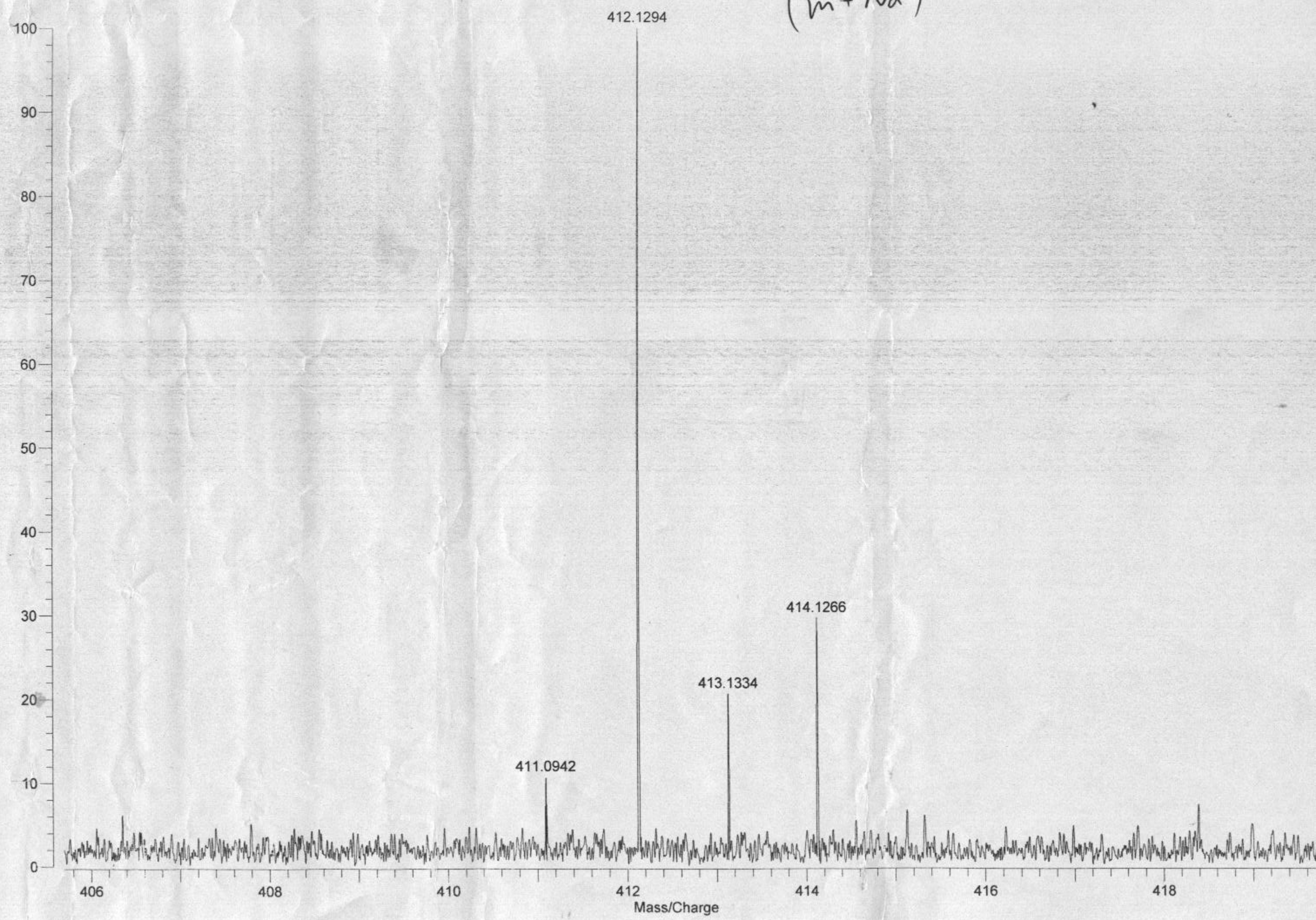

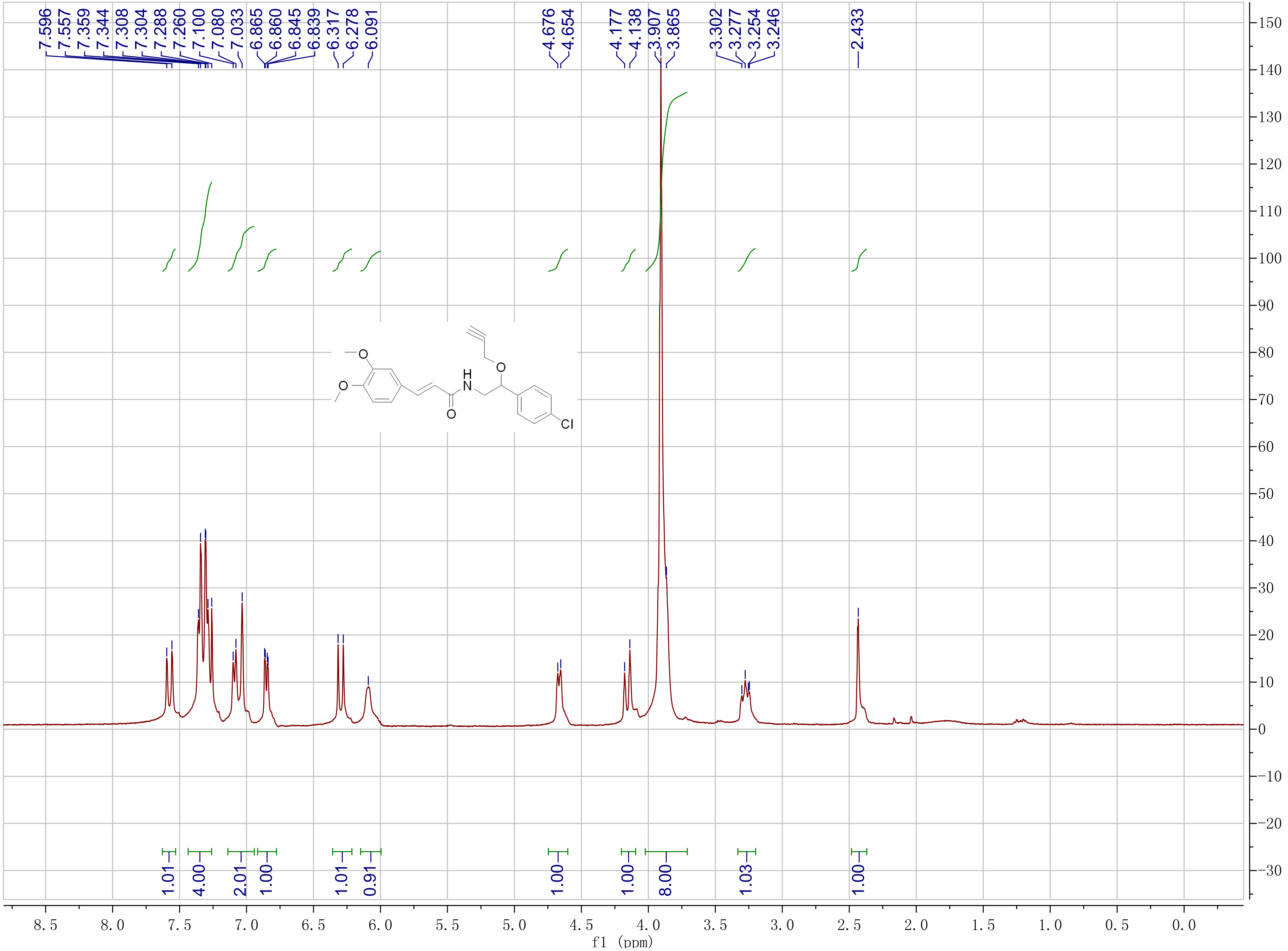
7.596
7.557
7.359
7.344
7.308
7.304
7.288
7.260
7.100
7.080
7.033
6.865
6.860
6.845
6.839
6.317
6.278
6.091
4.676
4.654
4.177
4.138
3.907
3.865
3.302
3.277
3.254
3.246
2.433
O
O
H
N
O
O
Cl
1.01
4.00
2.01
1.00
1.01
0.91
1.00
1.00
8.00
1.03
1.00
f1 (ppm)

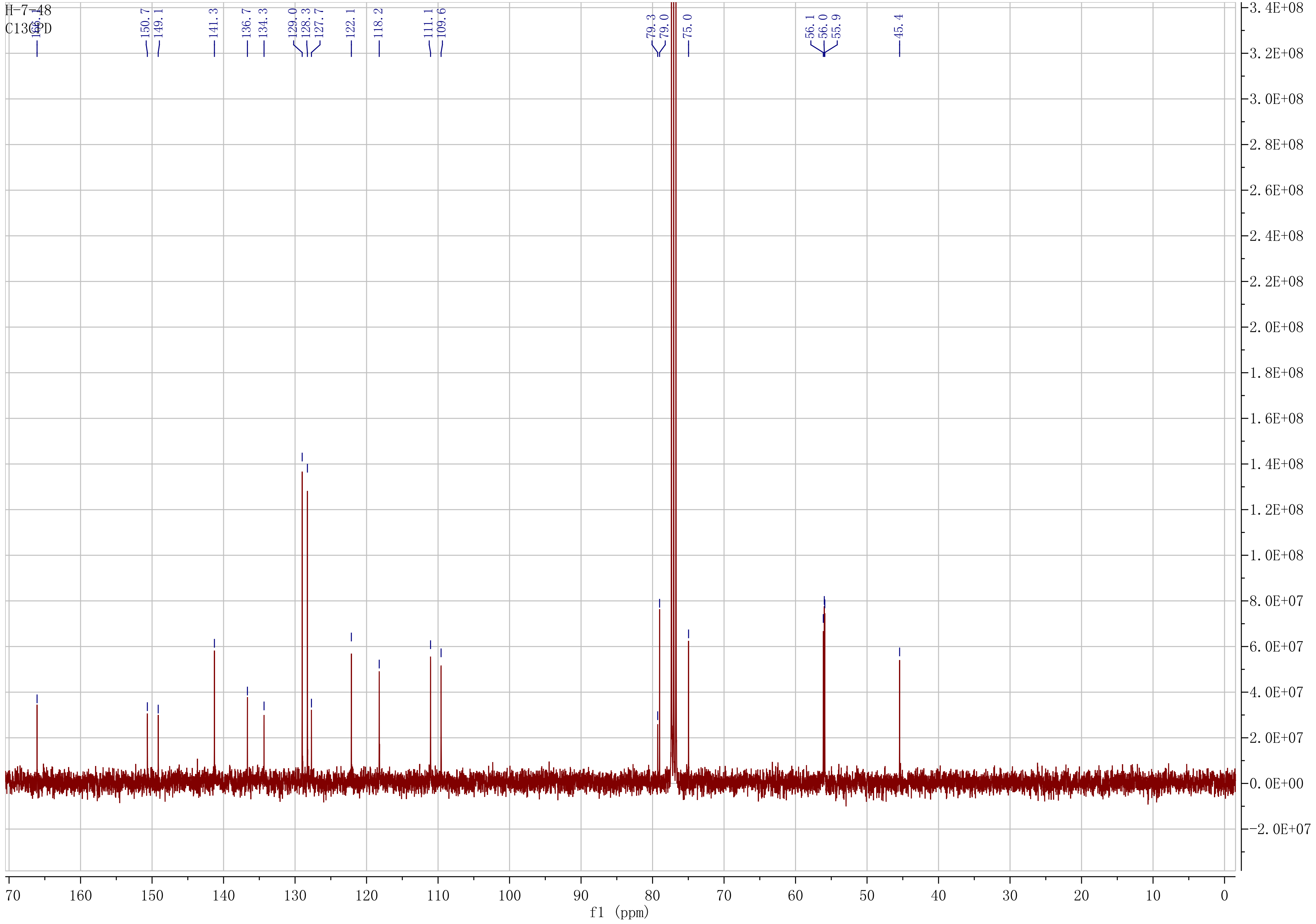

H-7-48
C13CPD
166.1
150.7
149.1
141.3
136.7
134.3
129.0
128.3
127.7
122.1
118.2
111.1
109.6
79.3
79.0
75.0
56.1
56.0
55.9
45.4
3.4E+08
3.2E+08
3.0E+08
2.8E+08
2.6E+08
2.4E+08
2.2E+08
2.0E+08
1.8E+08
1.6E+08
1.4E+08
1.2E+08
1.0E+08
8.0E+07
6.0E+07
4.0E+07
2.0E+07
0.0E+00
-2.0E+07
70
160
150
140
130
120
110
100
90
80
70
60
50
40
30
20
10
0
f1 (ppm)

Varian QFT-ESI
File: H-7-48_ESI.trans

Mode: Positive
Scans: 1

Date: 17-MAR-20
Time: 08:55:56
Scale: 8.3594

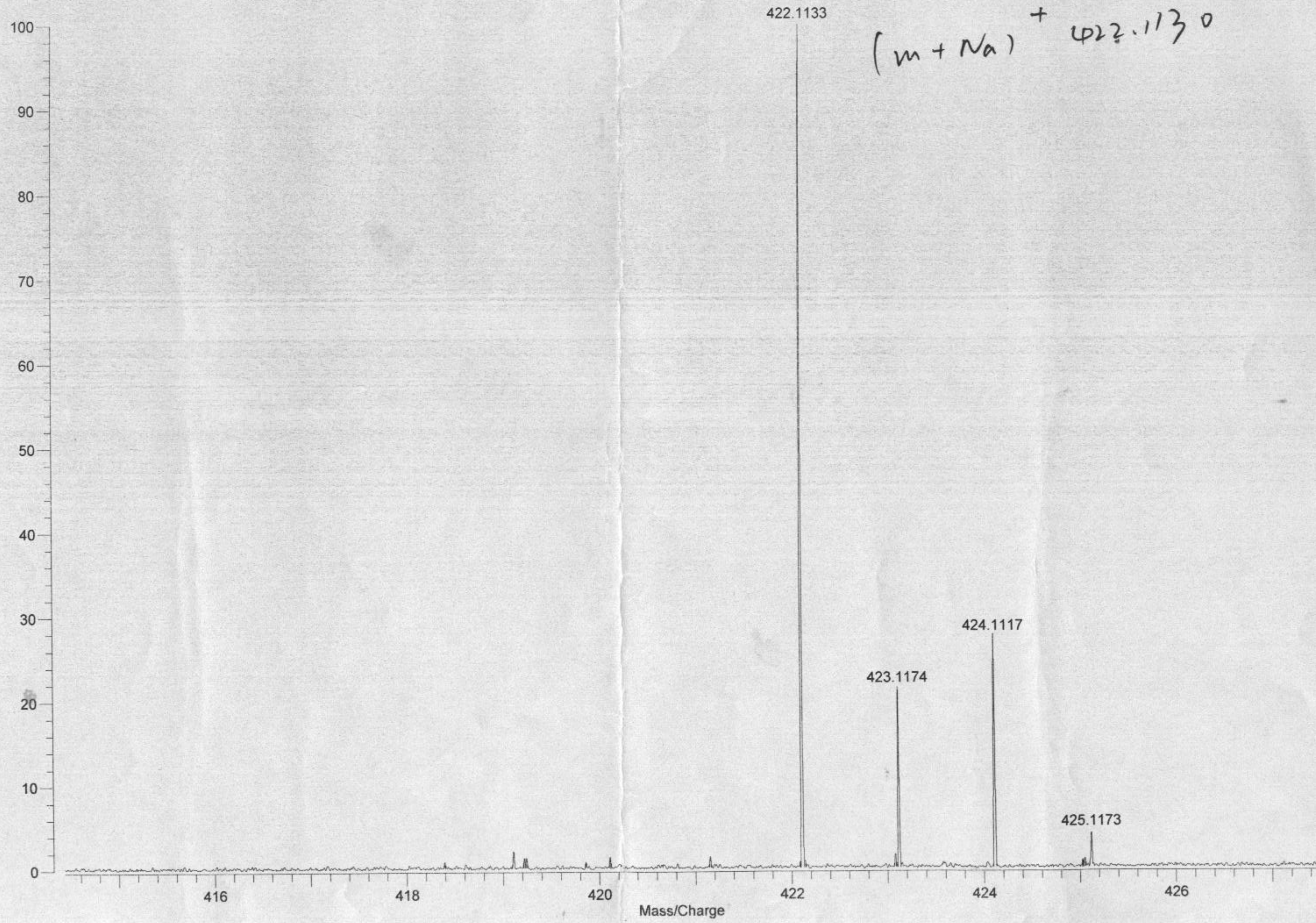

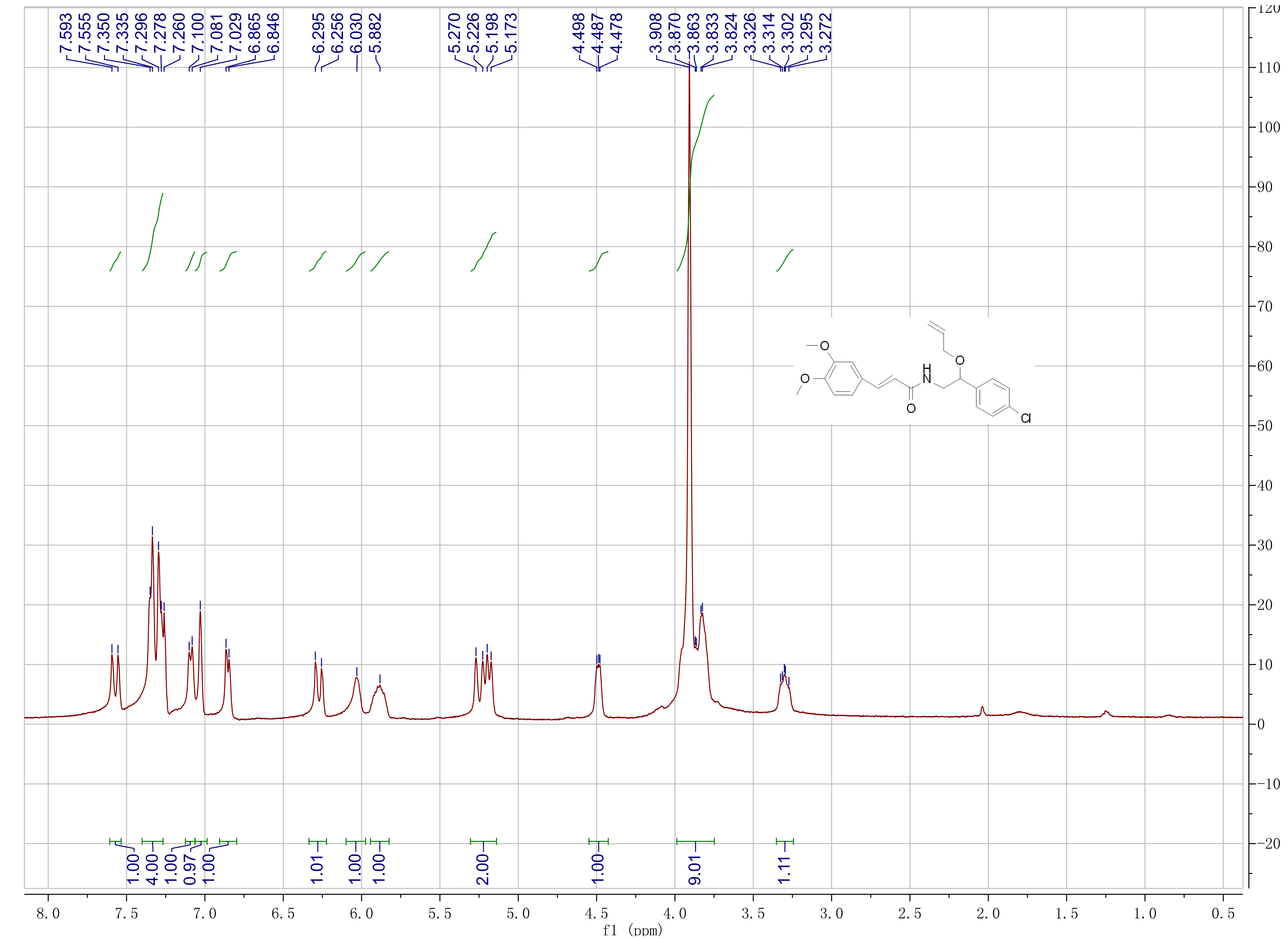
7.593
7.555
7.350
7.335
7.296
7.278
7.260
7.100
7.081
7.029
6.865
6.846
6.295
6.256
6.030
5.882
5.270
5.226
5.198
5.173
4.498
4.487
4.478
3.908
3.870
3.863
3.833
3.824
3.326
3.314
3.302
3.295
3.272
1.00
4.00
1.00
0.97
1.00
1.01
1.00
1.00
2.00
1.00
9.01
1.11
O
O
H
N
O
O
Cl
f1 (ppm)

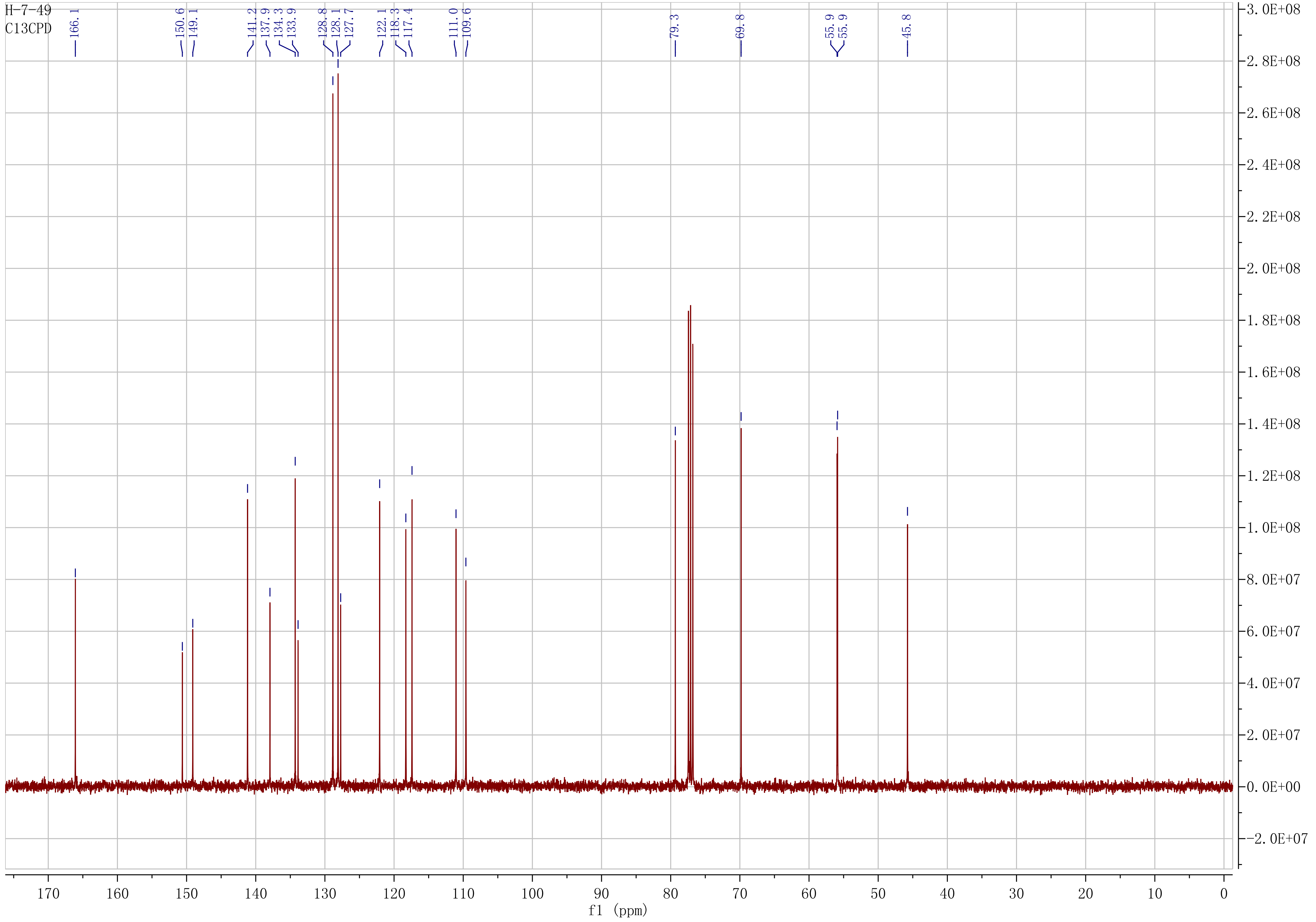
H-7-49
C13CPD
166.1
150.6
149.1
141.2
137.9
134.3
133.9
128.8
128.1
127.7
122.1
118.3
117.4
111.0
109.6
79.3
69.8
55.9
55.9
45.8
f1 (ppm)

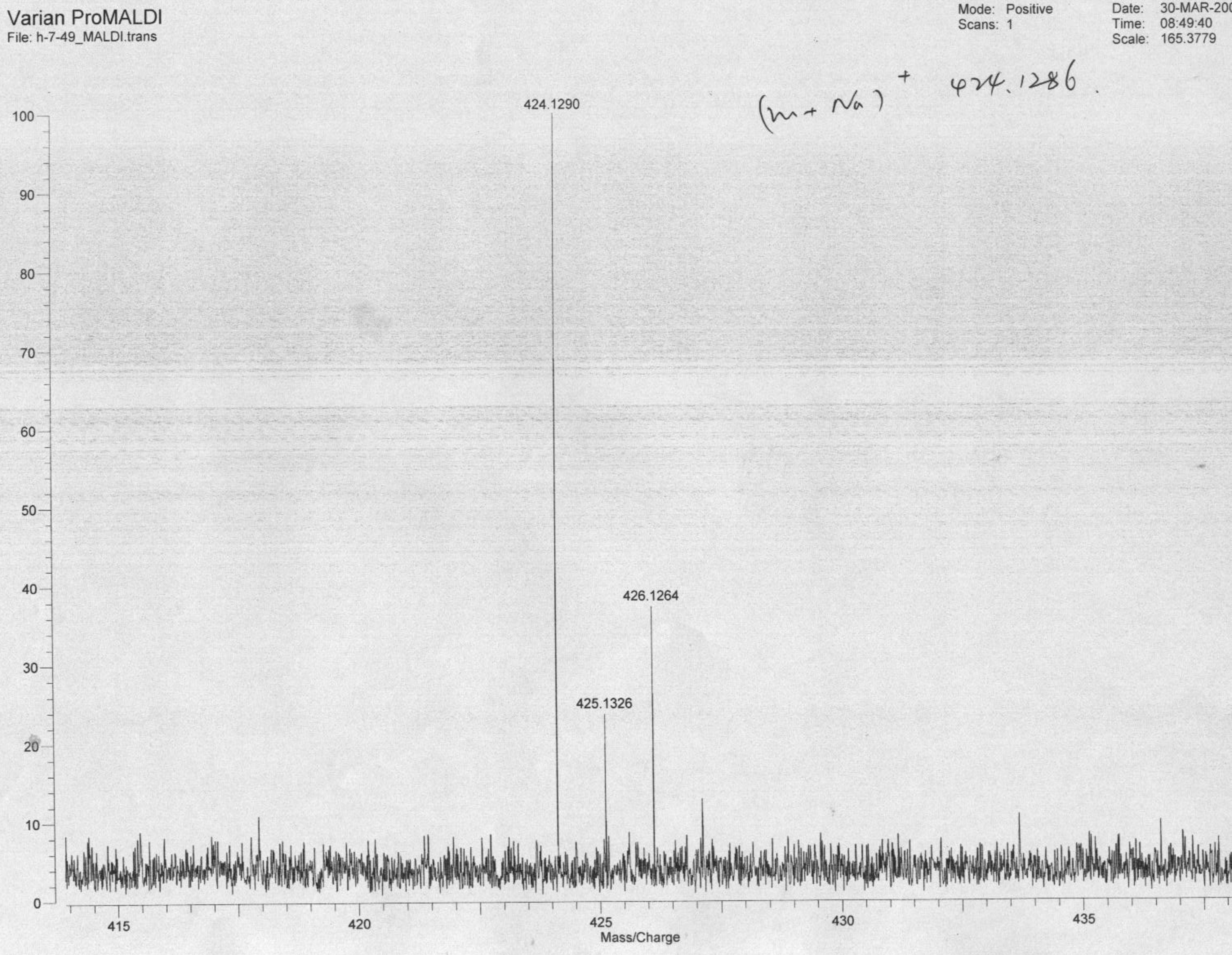

Varian ProMALDI
File: h-7-49_MALDI.trans
Mode: Positive
Scans: 1
Date: 30-MAR-200
Time: 08:49:40
Scale: 165.3779
(m+Na)+ 424.1286
424.1290
426.1264
425.1326
Mass/Charge

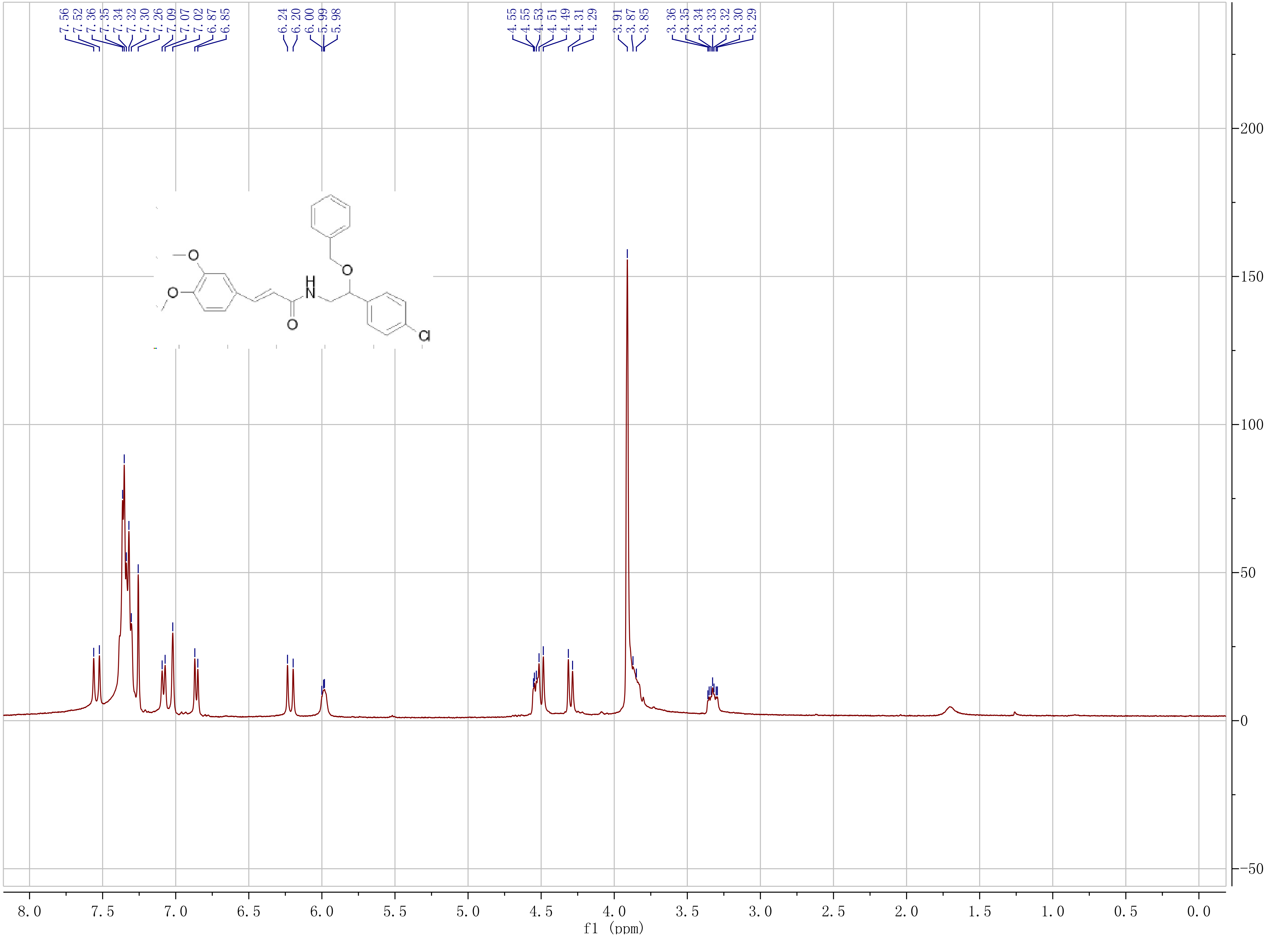
7.56 7.52 7.36 7.35 7.34 7.32 7.30 7.26 7.09 7.07 7.02 6.87 6.85
6.24 6.20 6.00 5.99 5.98
4.55 4.55 4.53 4.51 4.49 4.31 4.29
3.91 3.87 3.85
3.36 3.35 3.34 3.33 3.32 3.30 3.29
O
O
H
N
O
O
Cl
200
150
100
50
0
−50
8.0 7.5 7.0 6.5 6.0 5.5 5.0 4.5 4.0 3.5 3.0 2.5 2.0 1.5 1.0 0.5 0.0
f1 (ppm)

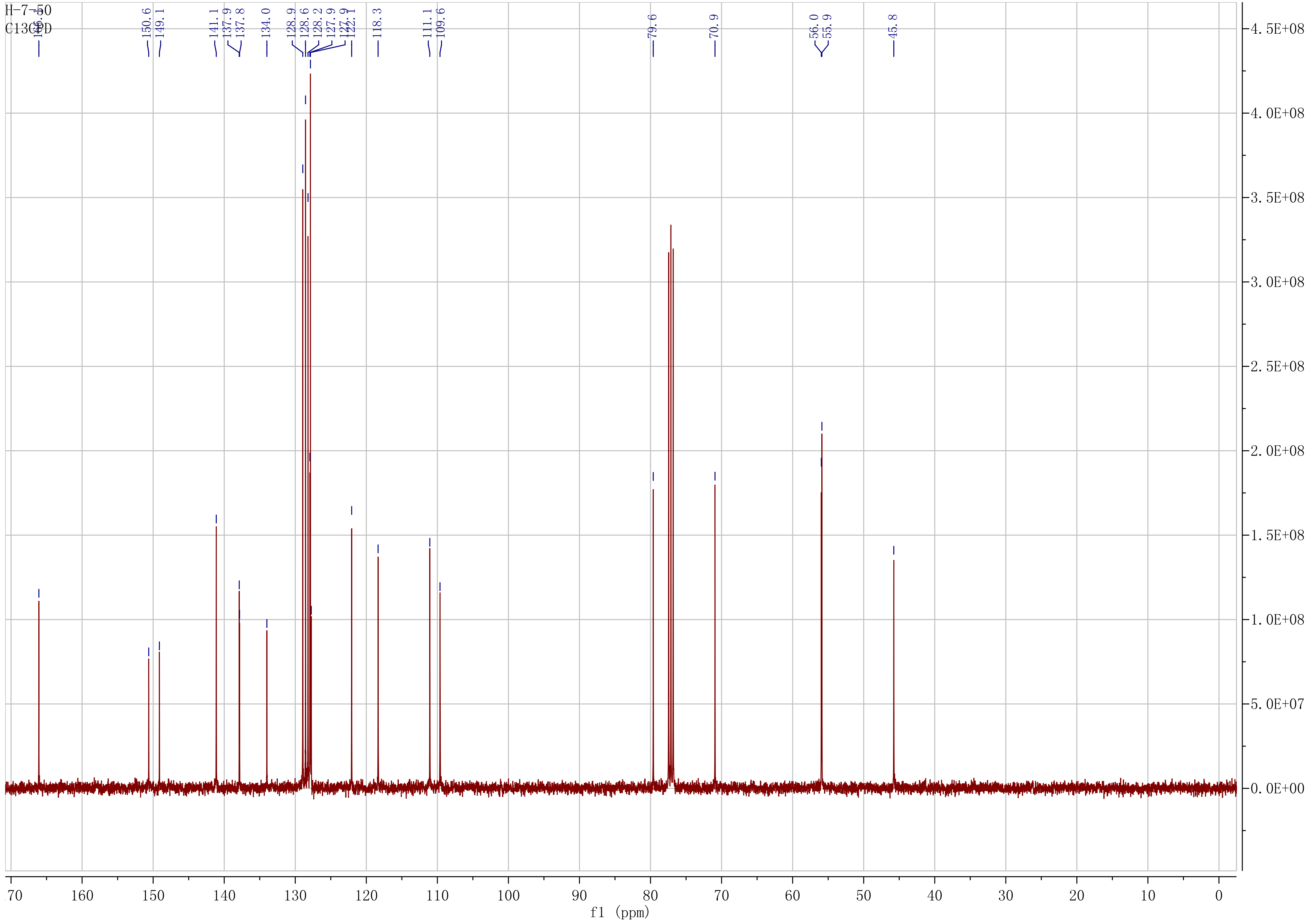
H-7-50
C13CPD
166.1
150.6
149.1
141.1
137.9
137.8
134.0
128.9
128.6
128.2
127.9
127.9
122.1
118.3
111.1
109.6
79.6
70.9
56.0
55.9
45.8
4.5E+08
4.0E+08
3.5E+08
3.0E+08
2.5E+08
2.0E+08
1.5E+08
1.0E+08
5.0E+07
0.0E+00
170
160
150
140
130
120
110
100
90
80
70
60
50
40
30
20
10
0
f1 (ppm)

| | | | | | | | |
|---|---|---|---|---|---|---|---|
| **Sample Name** | lc/ms | **Position** | Vial 3 | **Instrument Name** | Instrument 1 | **User Name** | |
| **Inj Vol** | -1 | **InjPosition** | | **SampleType** | Sample | **IRM Calibration Status** | Some Ions Missed |
| **Data Filename** | H-7-50.d | **ACQ Method** | | **Comment** | | **Acquired Time** | 4/9/2009 8:22:23 AM |

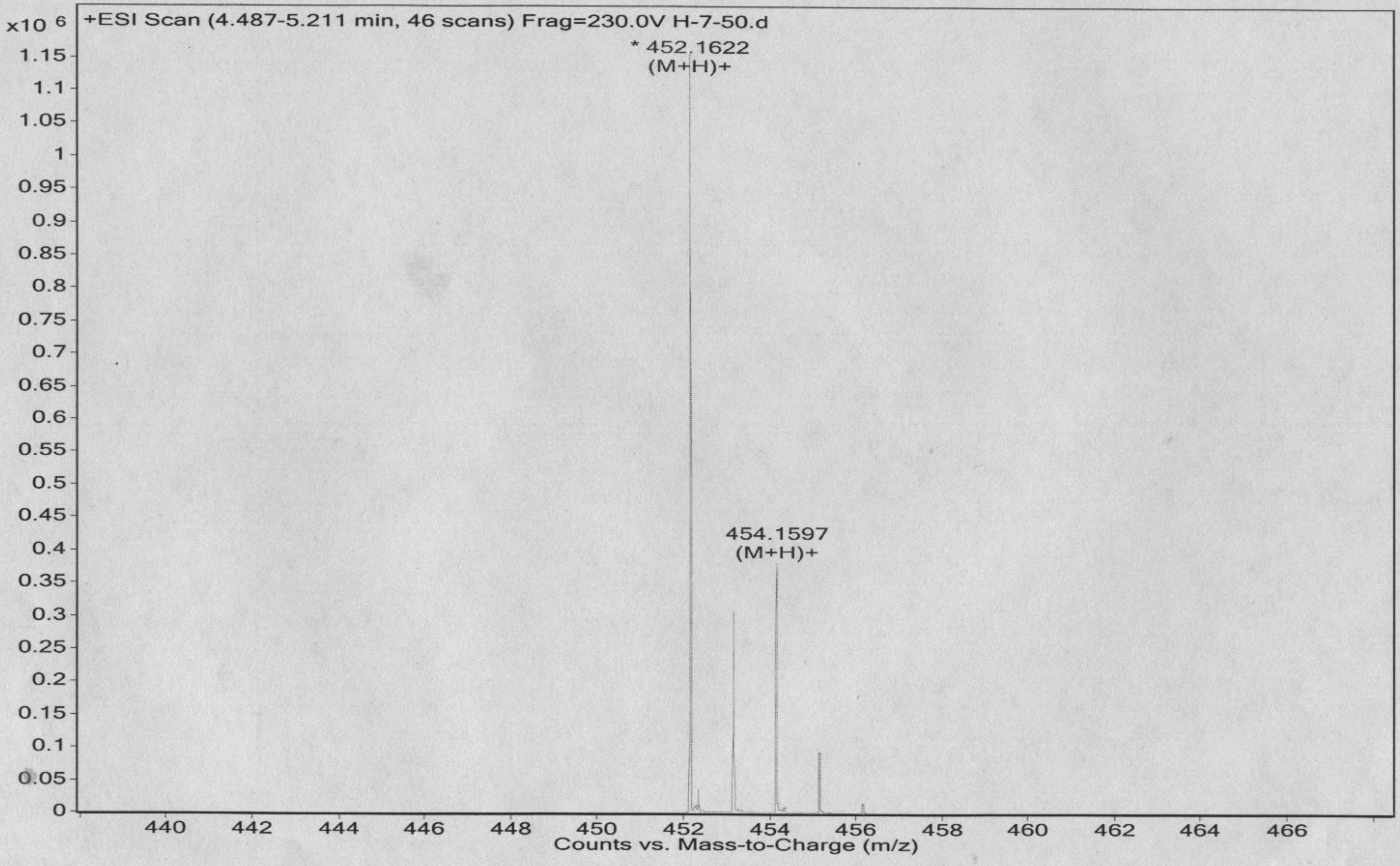

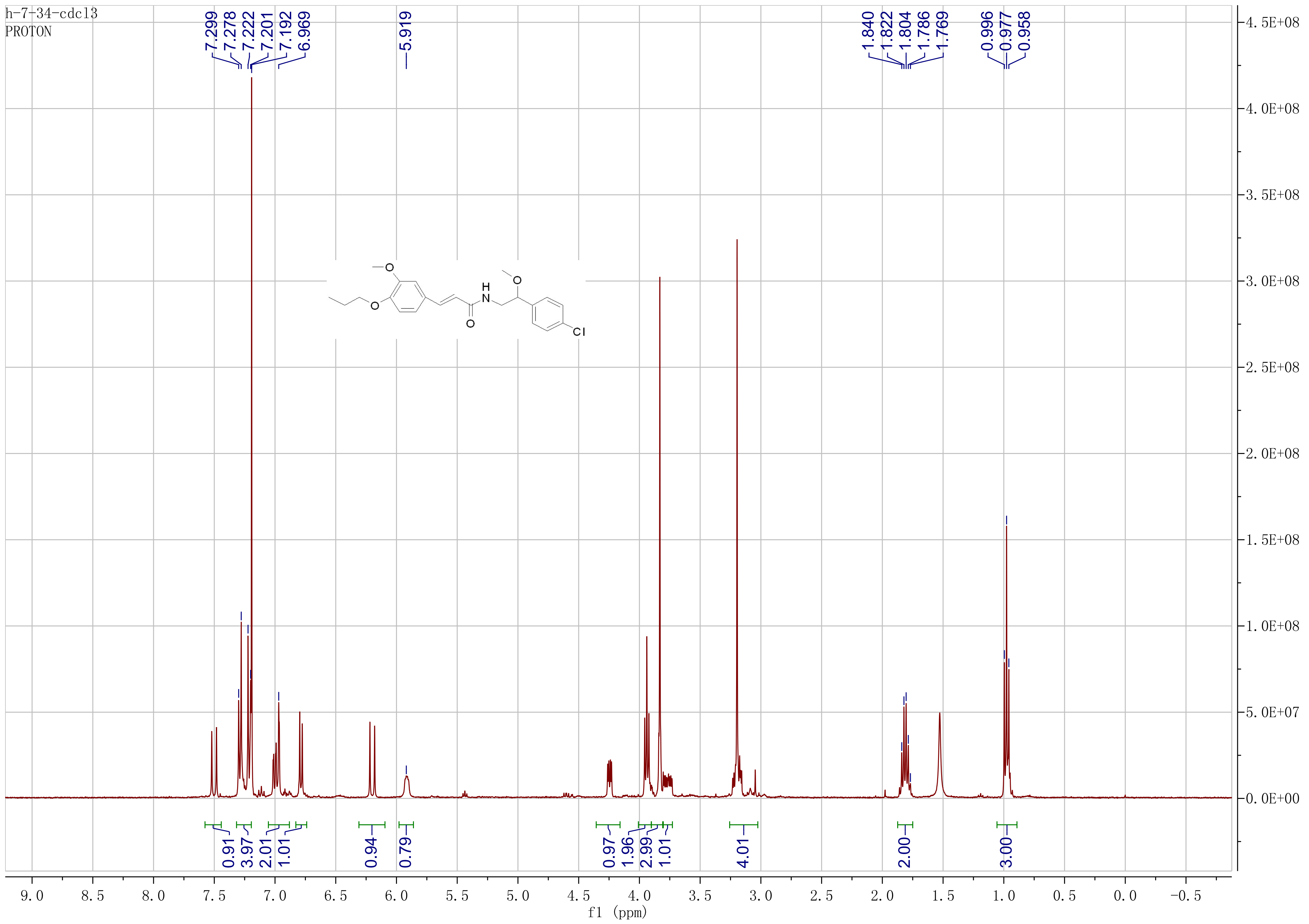
h-7-34-cdcl3
PROTON
7.299
7.278
7.222
7.201
7.192
6.969
5.919
1.840
1.822
1.804
1.786
1.769
0.996
0.977
0.958
0.91
3.97
2.01
1.01
0.94
0.79
0.97
1.96
2.99
1.01
4.01
2.00
3.00
H
N
O
O
O
O
Cl
f1 (ppm)

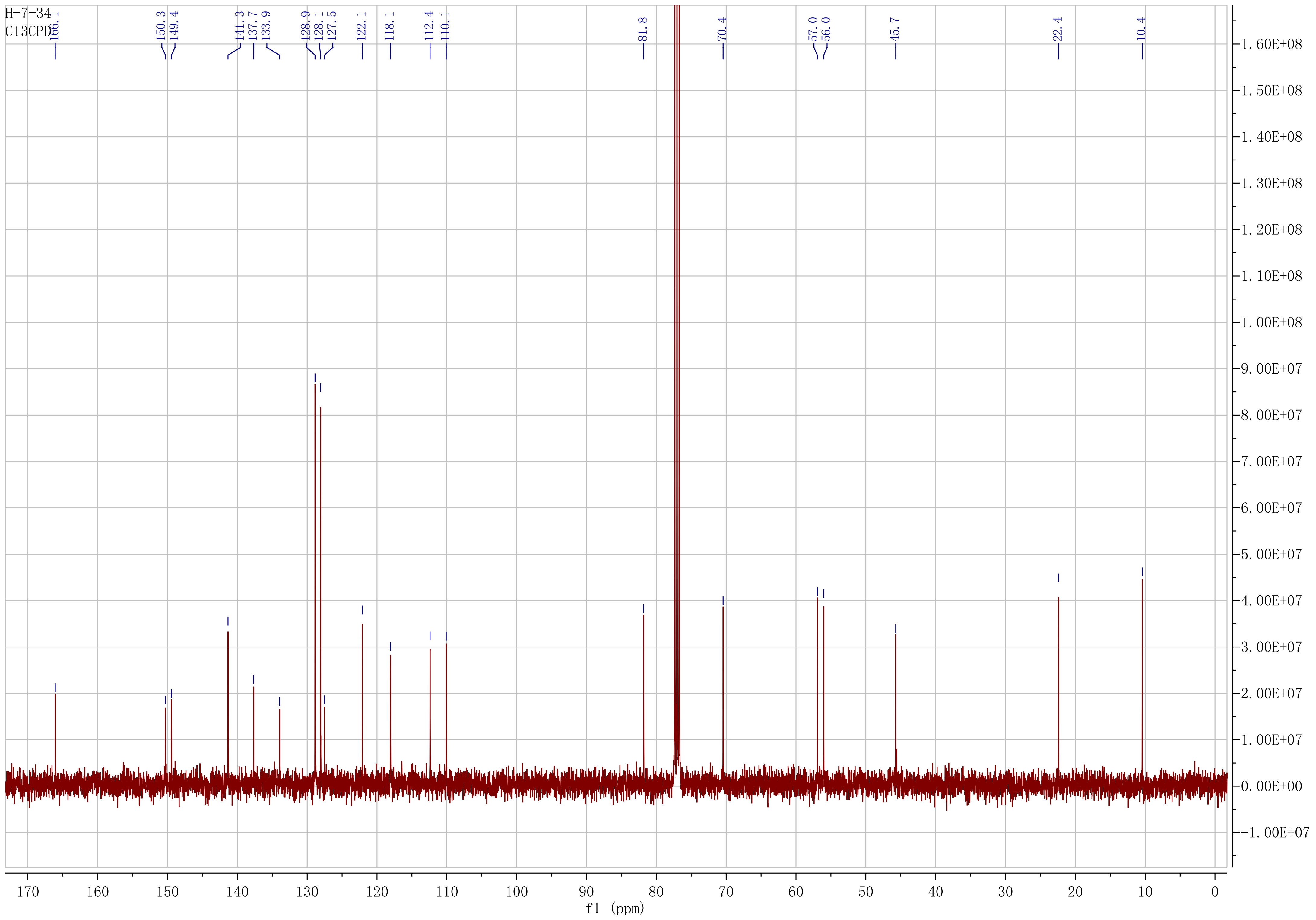
H-7-34
C13CPD
166.1
150.3
149.4
141.3
137.7
133.9
128.9
128.1
127.5
122.1
118.1
112.4
110.1
81.8
70.4
57.0
56.0
45.7
22.4
10.4
f1 (ppm)

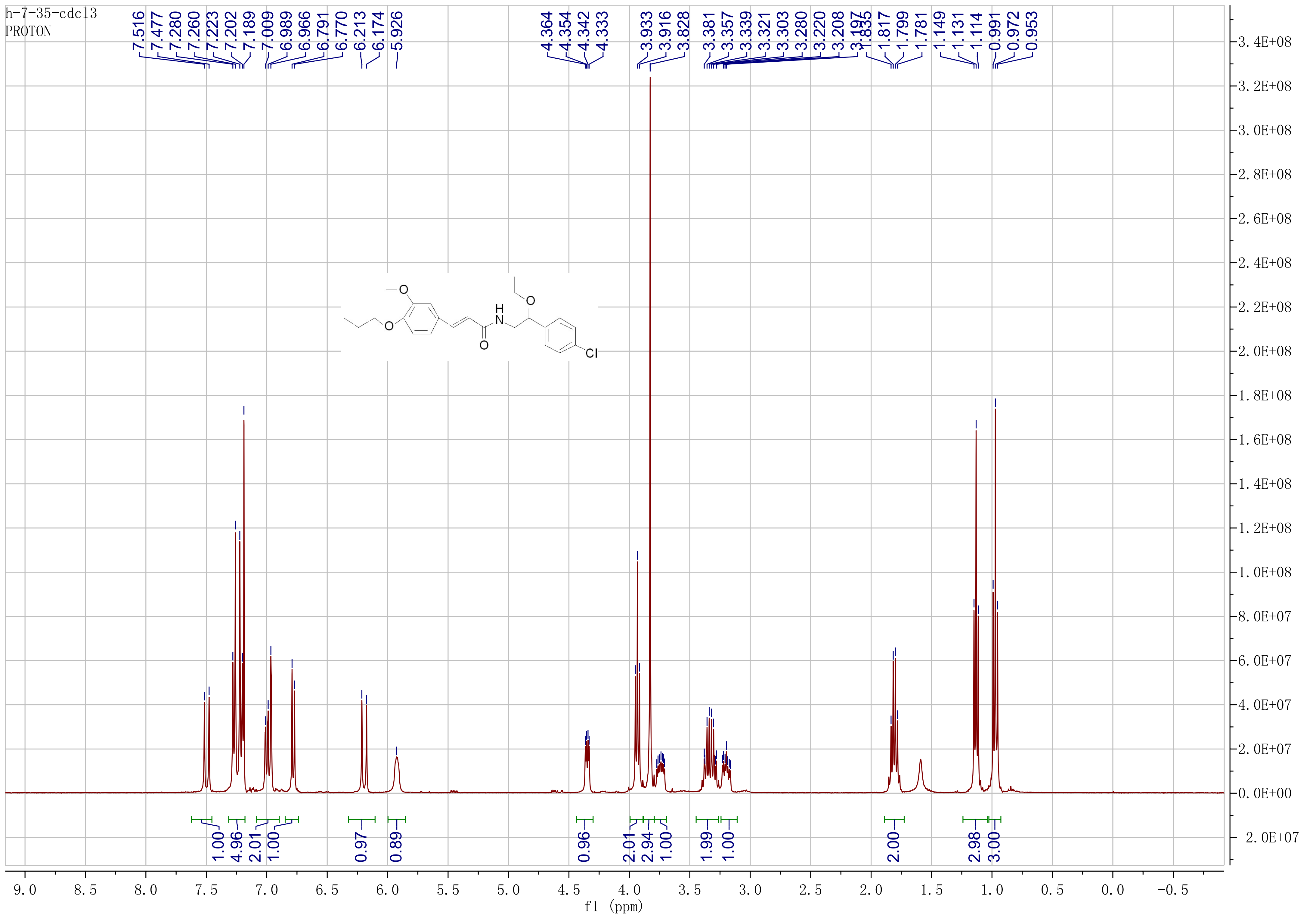

h-7-35-cdcl3
PROTON
7.516
7.477
7.280
7.260
7.223
7.202
7.189
7.009
6.989
6.966
6.791
6.770
6.213
6.174
5.926
4.364
4.354
4.342
4.333
3.933
3.916
3.828
3.381
3.357
3.339
3.321
3.303
3.280
3.220
3.208
3.197
1.835
1.817
1.799
1.781
1.149
1.131
1.114
0.991
0.972
0.953
1.00
4.96
2.01
1.00
0.97
0.89
0.96
2.01
2.94
1.00
1.99
1.00
2.00
2.98
3.00
O
H
N
O
O
O
Cl
f1 (ppm)

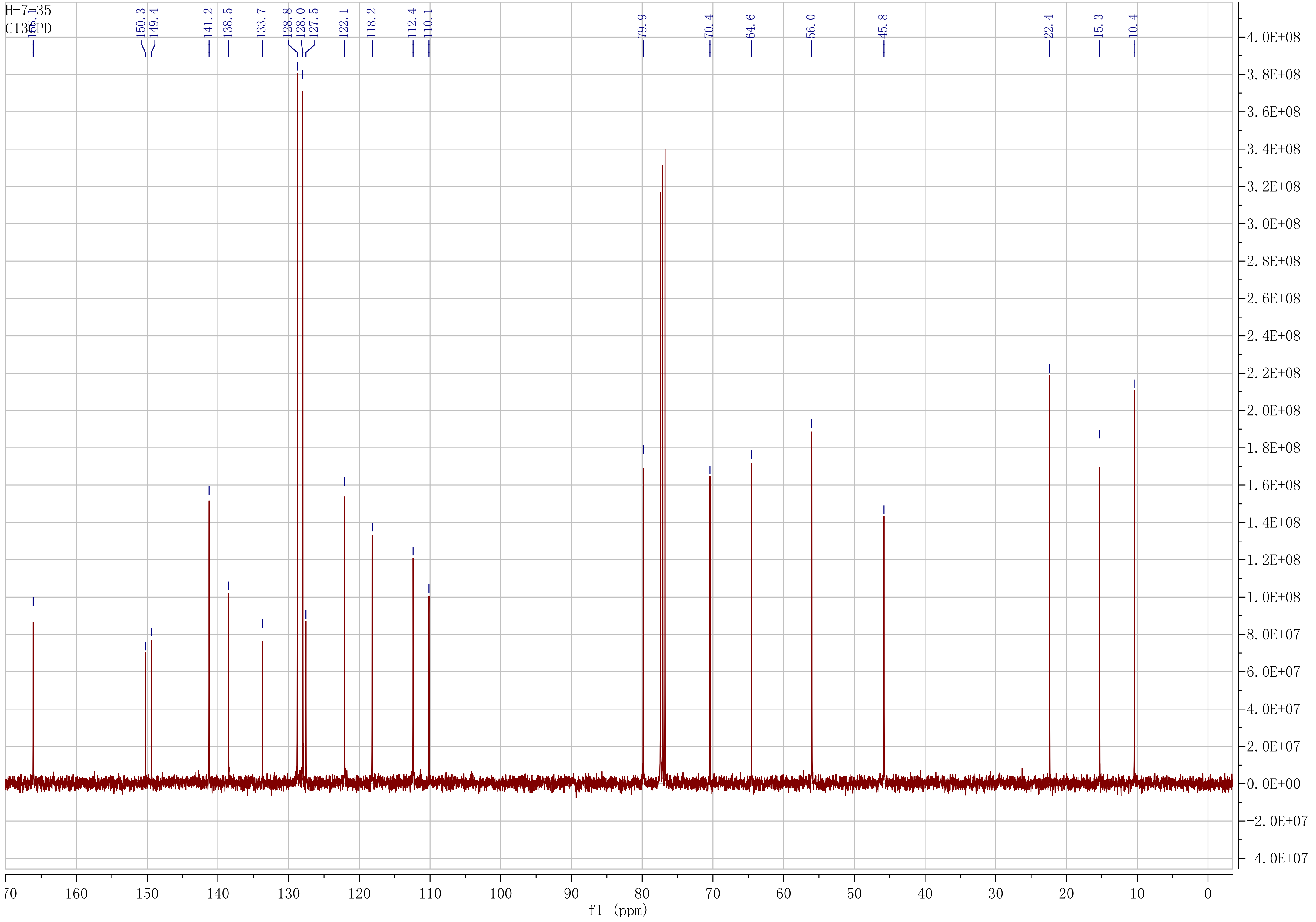
H-7-35
C13CPD
166.1
150.3
149.4
141.2
138.5
133.7
128.8
128.0
127.5
122.1
118.2
112.4
110.1
79.9
70.4
64.6
56.0
45.8
22.4
15.3
10.4
4.0E+08
3.8E+08
3.6E+08
3.4E+08
3.2E+08
3.0E+08
2.8E+08
2.6E+08
2.4E+08
2.2E+08
2.0E+08
1.8E+08
1.6E+08
1.4E+08
1.2E+08
1.0E+08
8.0E+07
6.0E+07
4.0E+07
2.0E+07
0.0E+00
-2.0E+07
-4.0E+07
70
160
150
140
130
120
110
100
90
80
70
60
50
40
30
20
10
0
f1 (ppm)

Varian ProMALDI
File: h-7-35_MALDI.trans

Mode: Positive
Scans: 1

Date: 30-MAR-200
Time: 08:43:01
Scale: 202.0668

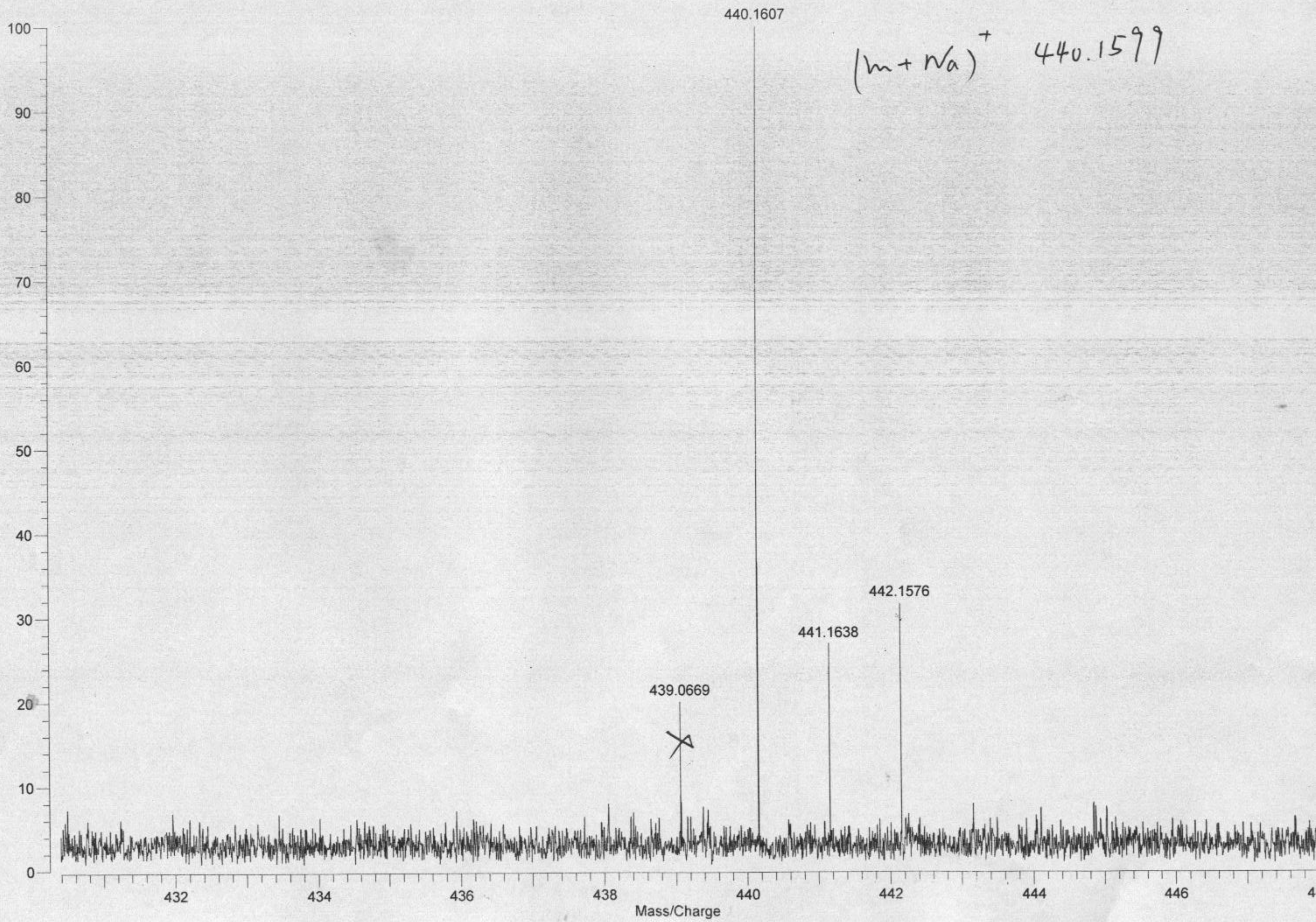

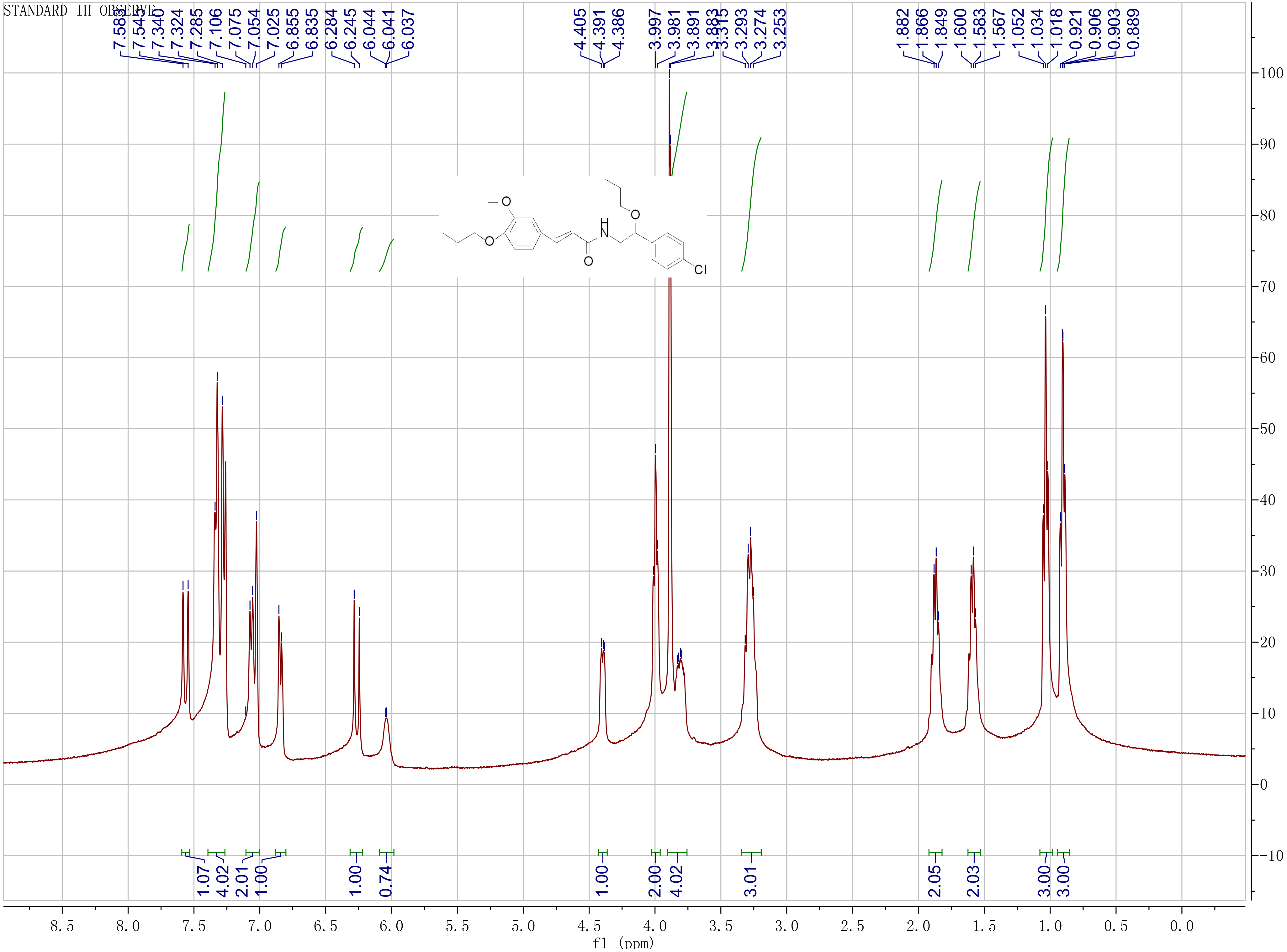
STANDARD 1H OBSERVE
7.545
7.340
7.324
7.285
7.106
7.075
7.054
7.025
6.855
6.835
6.284
6.245
6.044
6.041
6.037
4.405
4.391
4.386
3.997
3.981
3.891
3.315
3.293
3.274
3.253
1.882
1.866
1.849
1.600
1.583
1.567
1.052
1.034
1.018
0.921
0.906
0.903
0.889
O
O
H
N
O
O
Cl
1.07
4.02
2.01
1.00
1.00
0.74
1.00
2.00
4.02
3.01
2.05
2.03
3.00
3.00
f1 (ppm)

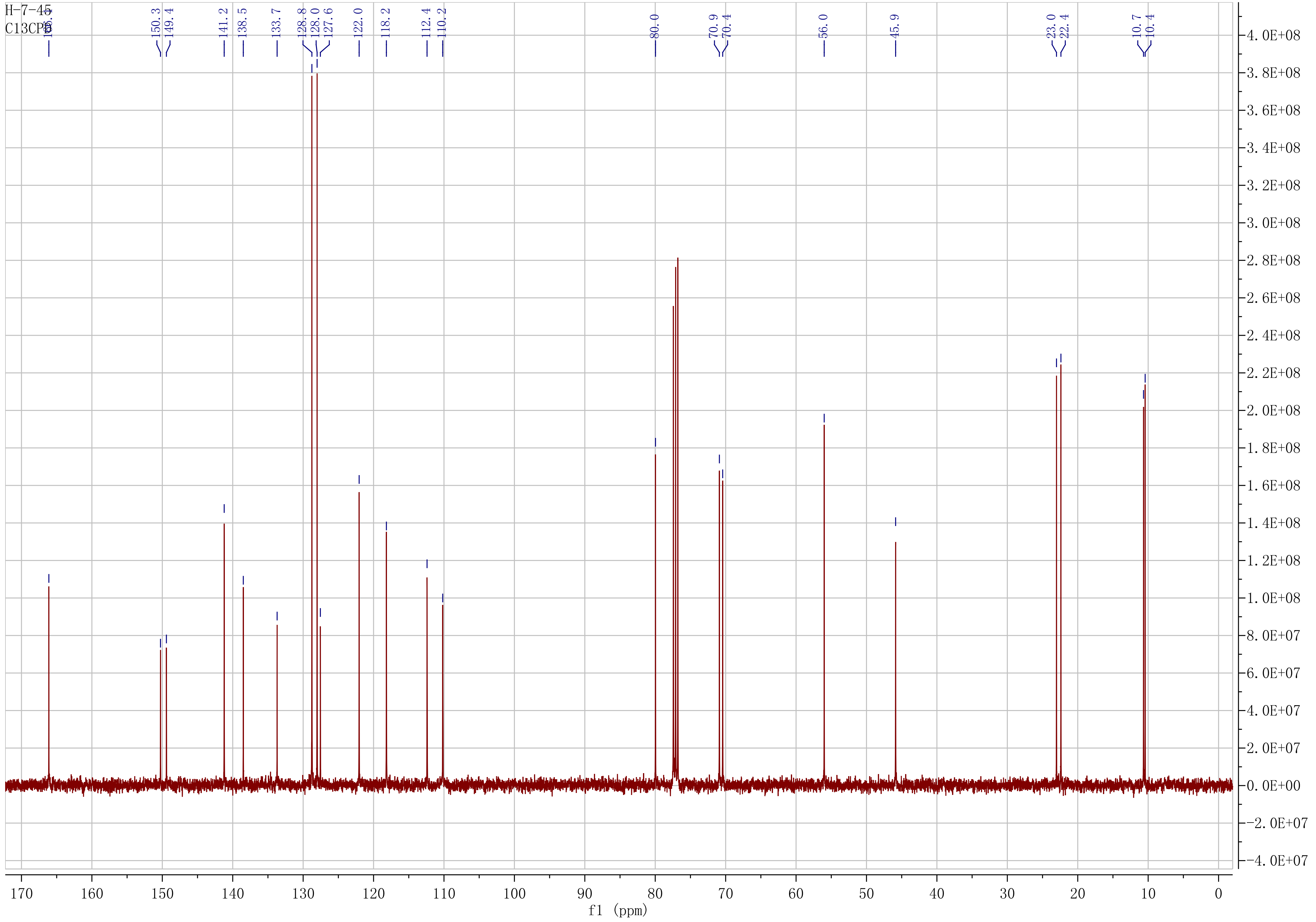

H-7-45
C13CPD
150.3
149.4
141.2
138.5
133.7
128.8
128.0
127.6
122.0
118.2
112.4
110.2
80.0
70.9
70.4
56.0
45.9
23.0
22.4
10.7
10.4
f1 (ppm)

Varian ProMALDI
File: h-7-45_MALDI.trans

Mode: Positive
Scans: 1

Date: 29-MAR-200
Time: 20:19:42
Scale: 64.7860

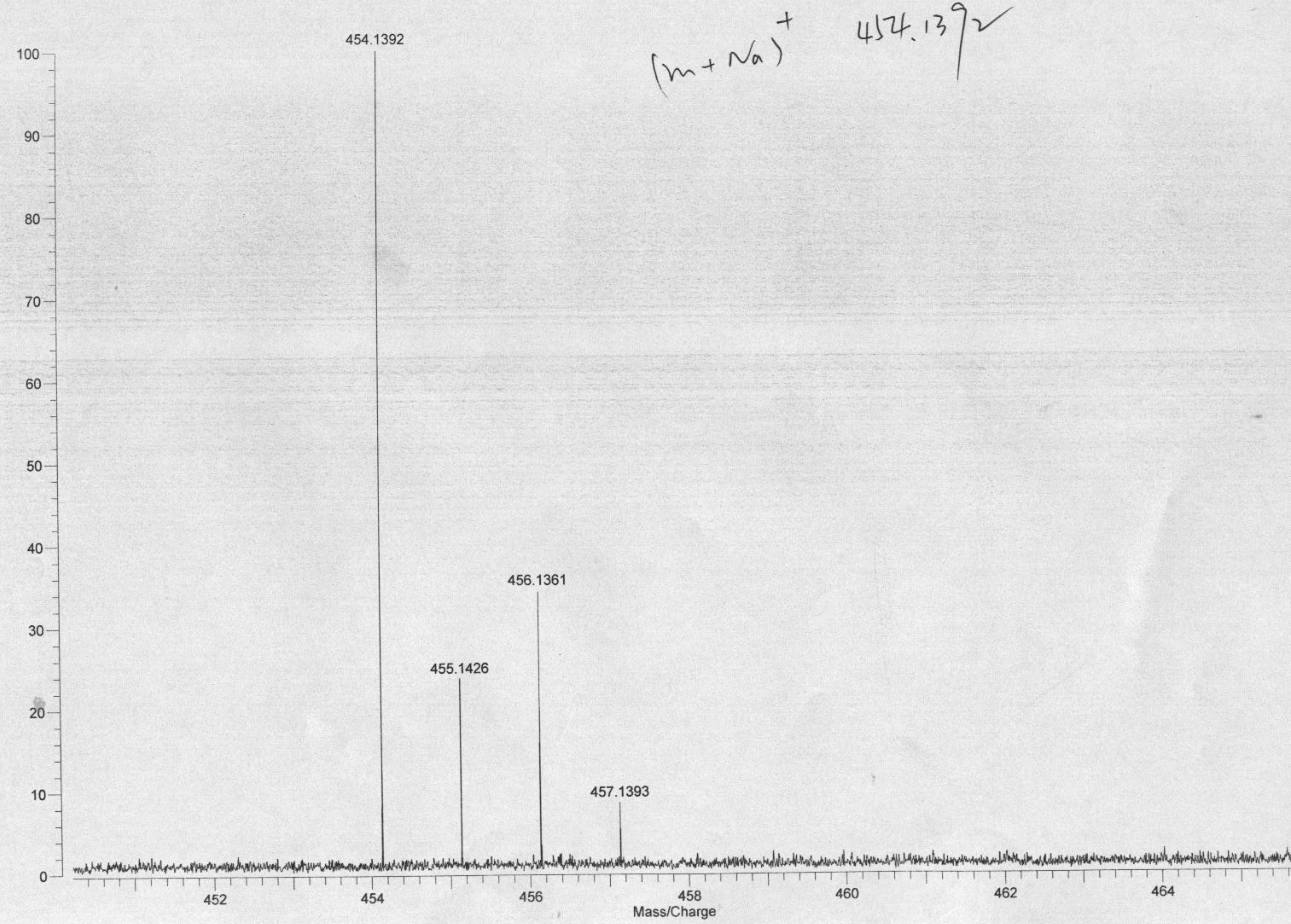

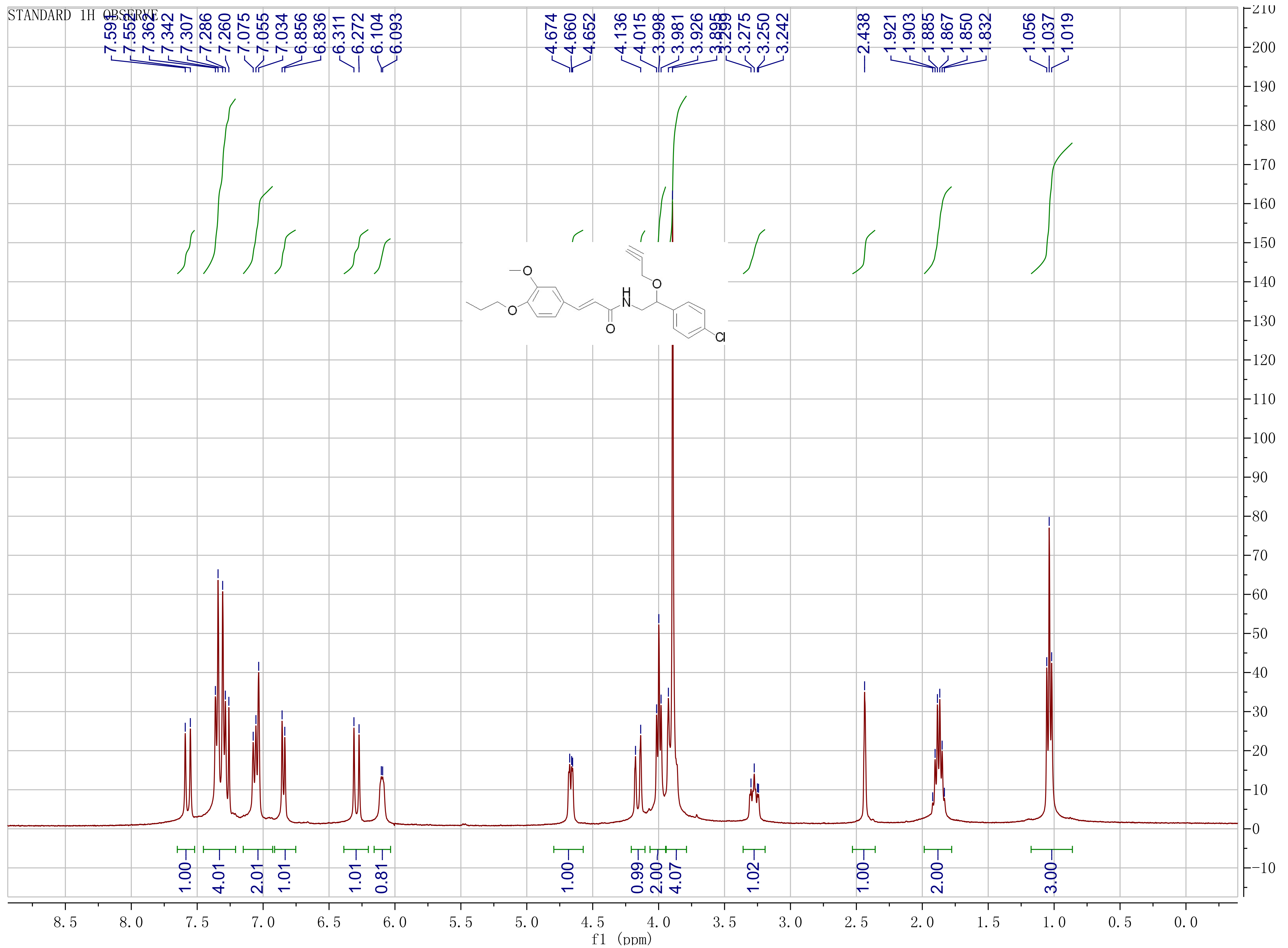
STANDARD 1H OBSERVE
7.342
7.307
7.286
7.260
7.075
7.055
7.034
6.856
6.836
6.311
6.272
6.104
6.093
4.674
4.660
4.652
4.136
4.015
3.998
3.981
3.926
3.275
3.250
3.242
2.438
1.921
1.903
1.885
1.867
1.850
1.832
1.056
1.037
1.019
1.00
4.01
2.01
1.01
1.01
0.81
1.00
0.99
2.00
4.07
1.02
1.00
2.00
3.00
O
H
N
O
O
O
Cl
8.5
8.0
7.5
7.0
6.5
6.0
5.5
5.0
4.5
4.0
3.5
3.0
2.5
2.0
1.5
1.0
0.5
0.0
f1 (ppm)

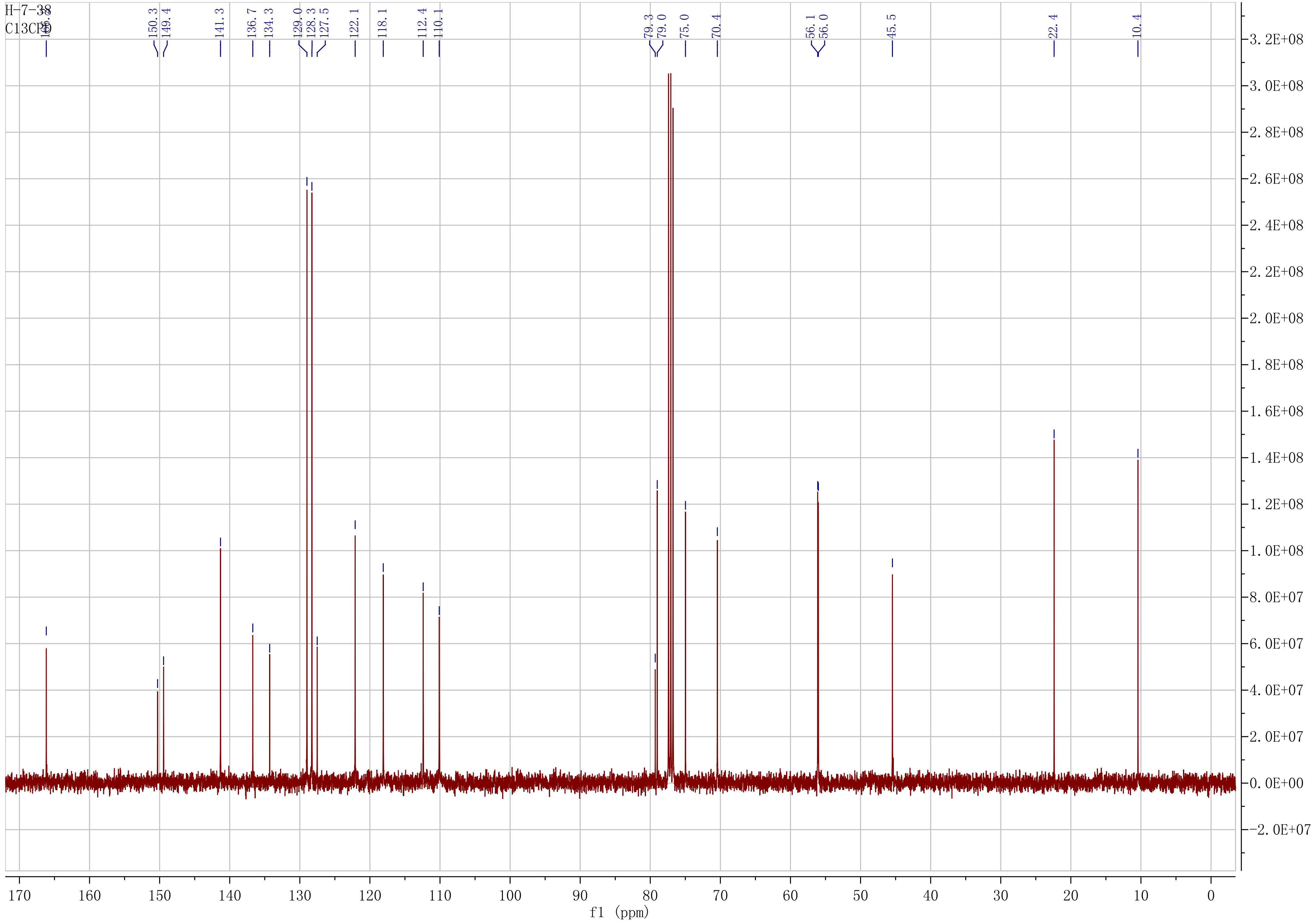

H-7-38
C13CPD
166.2
150.3
149.4
141.3
136.7
134.3
129.0
128.3
127.5
122.1
118.1
112.4
110.1
79.3
79.0
75.0
70.4
56.1
56.0
45.5
22.4
10.4
f1 (ppm)

| | | | | | | | |
|---|---|---|---|---|---|---|---|
| **Sample Name** | lc/ms | **Position** | Vial 3 | **Instrument Name** | Instrument 1 | **User Name** | |
| **Inj Vol** | -1 | **InjPosition** | | **SampleType** | Sample | **IRM Calibration Status** | Some Ions Missed |
| **Data Filename** | H-7-38.d | **ACQ Method** | | **Comment** | | **Acquired Time** | 4/9/2009 8:12:13 AM |

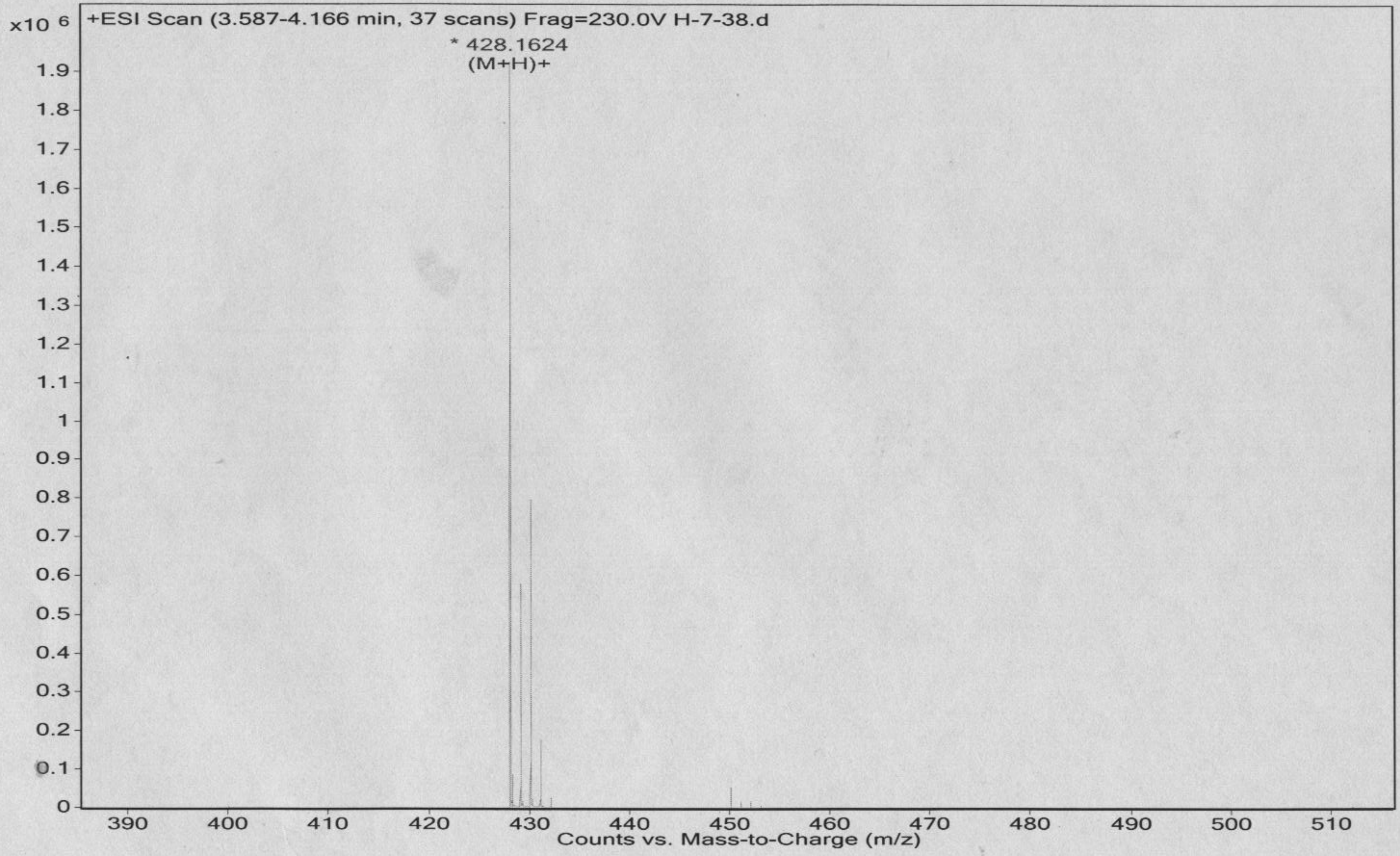

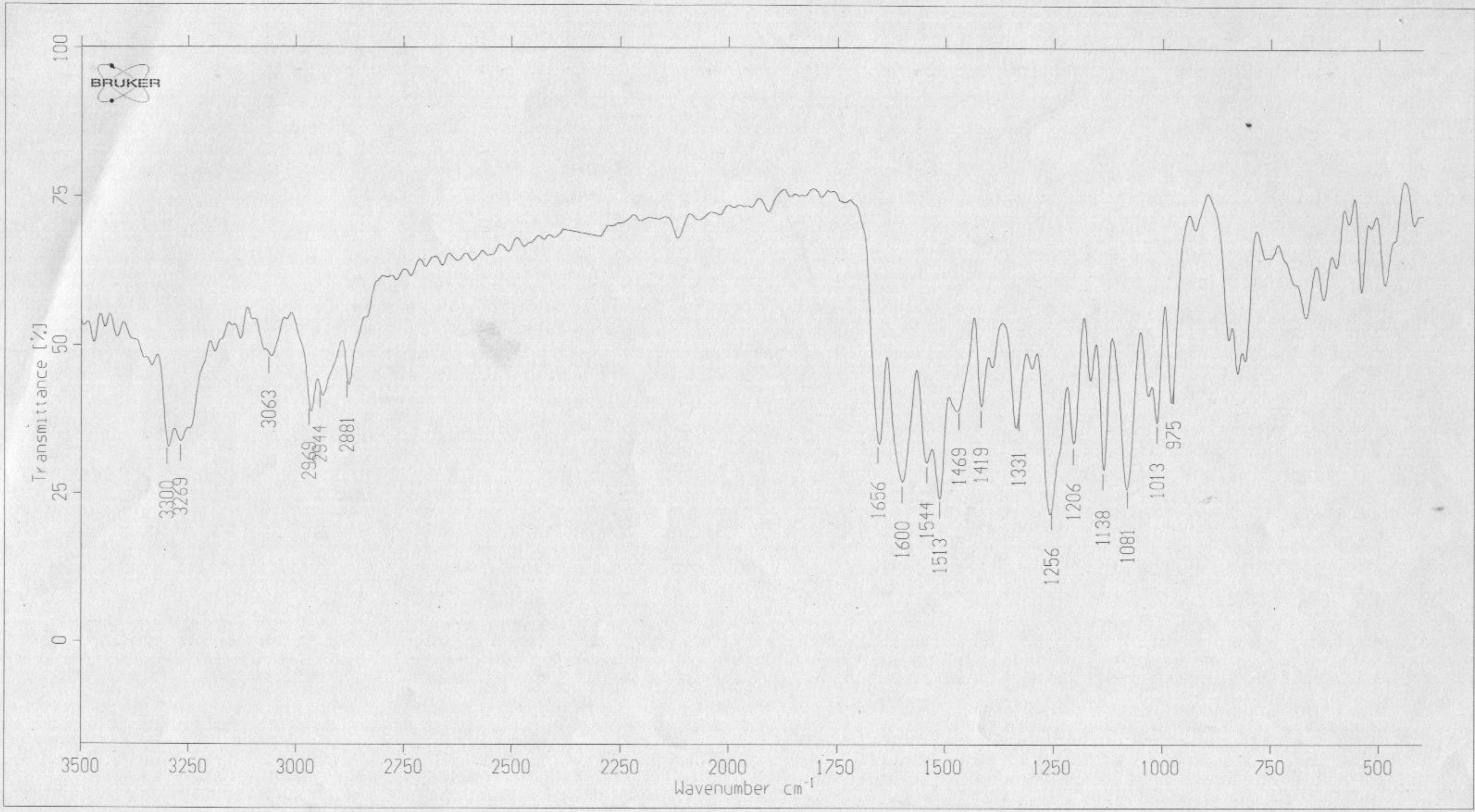


sample : H-7-38
technique :
customer :
comments :

freq range : 399.1926 - 3999.6401
resolution : 4.0
zerofilling : 2

Instrument : EQUINOX55
acq. m : Double Sided Forward-Backward

sample scans : 6

measured : 20/ 4/2009

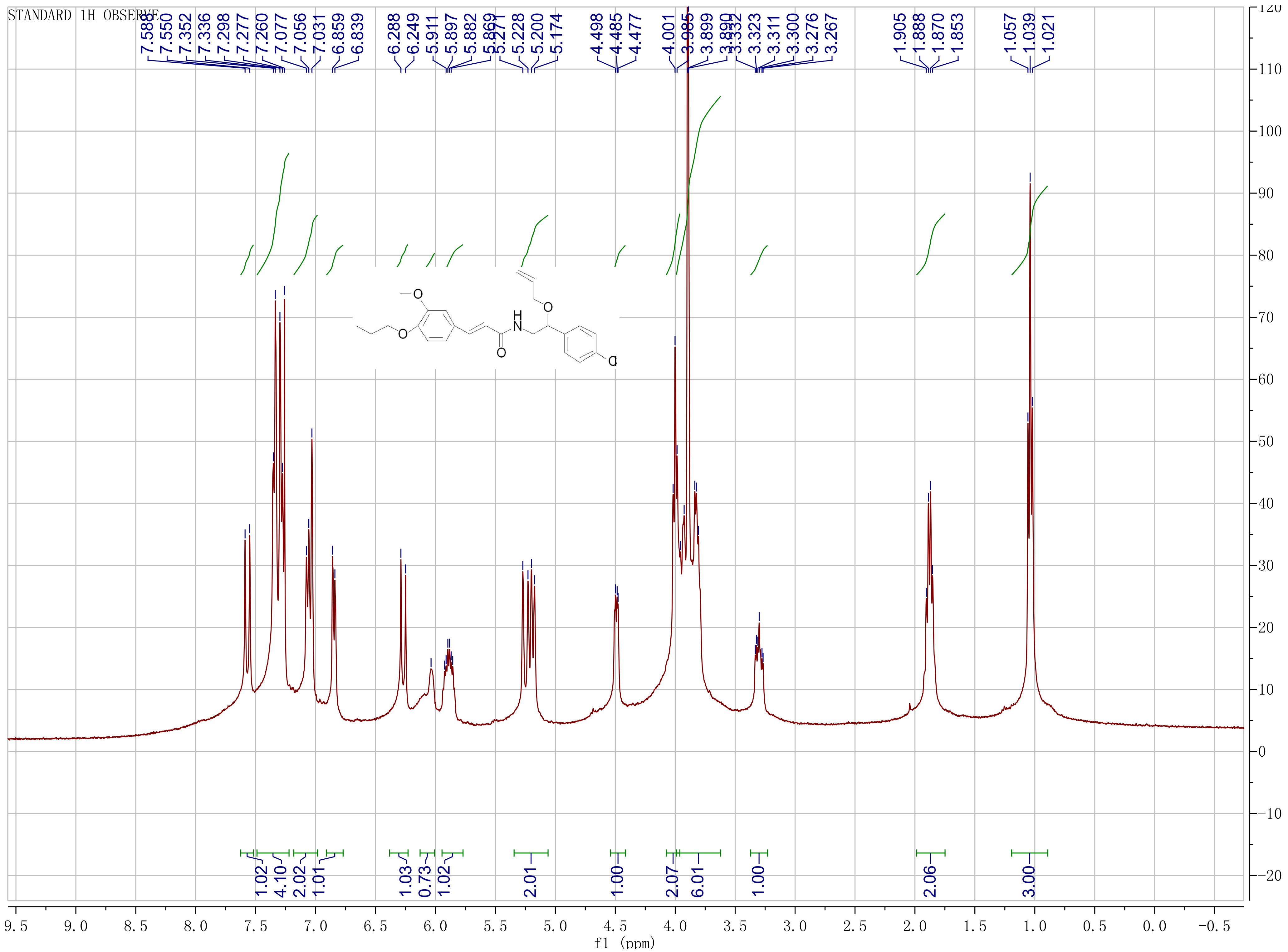
STANDARD 1H OBSERVE
7.588
7.550
7.352
7.336
7.298
7.277
7.260
7.077
7.056
7.031
6.859
6.839
6.288
6.249
5.911
5.897
5.882
5.869
5.271
5.228
5.200
5.174
4.498
4.485
4.477
4.001
3.985
3.899
3.890
3.332
3.323
3.311
3.300
3.276
3.267
1.905
1.888
1.870
1.853
1.057
1.039
1.021
O
O
H
N
O
O
Cl
1.02
4.10
2.02
1.01
1.03
0.73
1.02
2.01
1.00
2.07
6.01
1.00
2.06
3.00
f1 (ppm)

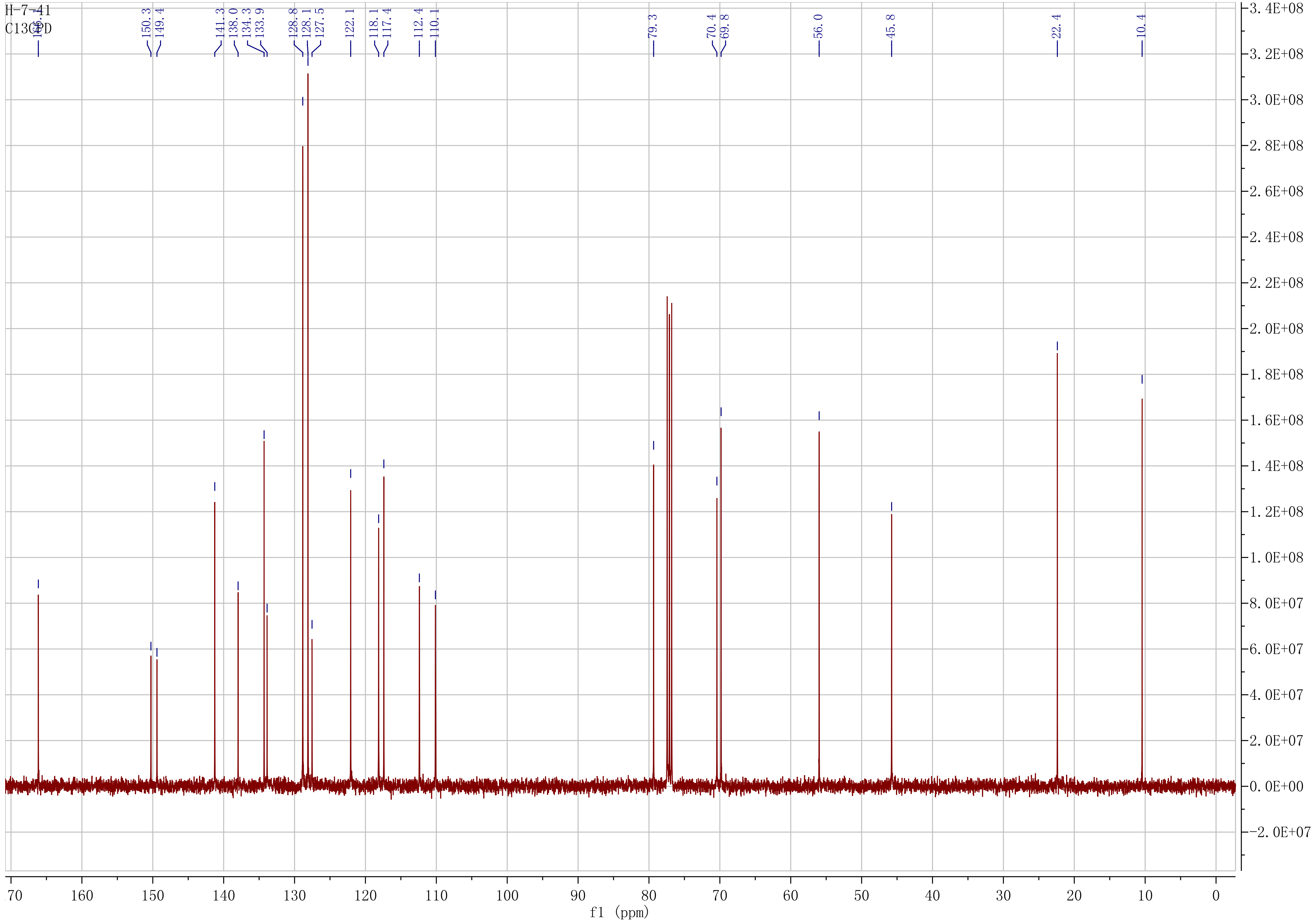
H-7-41
C13CPD
150.3
149.4
141.3
138.0
134.3
133.9
128.8
128.1
127.5
122.1
118.1
117.4
112.4
110.1
79.3
70.4
69.8
56.0
45.8
22.4
10.4
f1 (ppm)

Varian ProMALDI
File: h-7-41_MALDI.trans

Mode: Positive
Scans: 1

Date: 29-MAR-20[illegible]
Time: 19:45:44
Scale: 25.4524

(m+Na)+ 452.1599

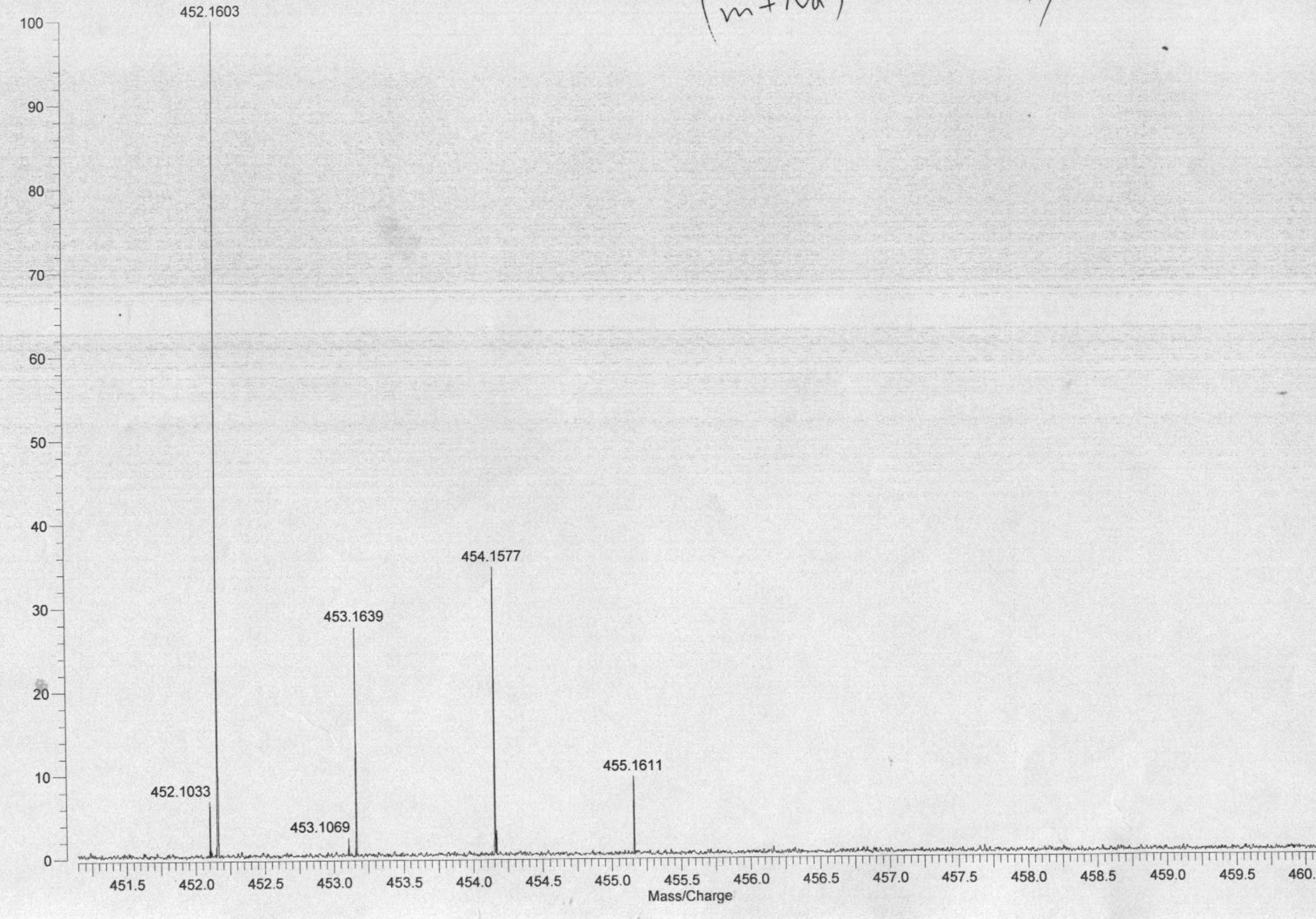

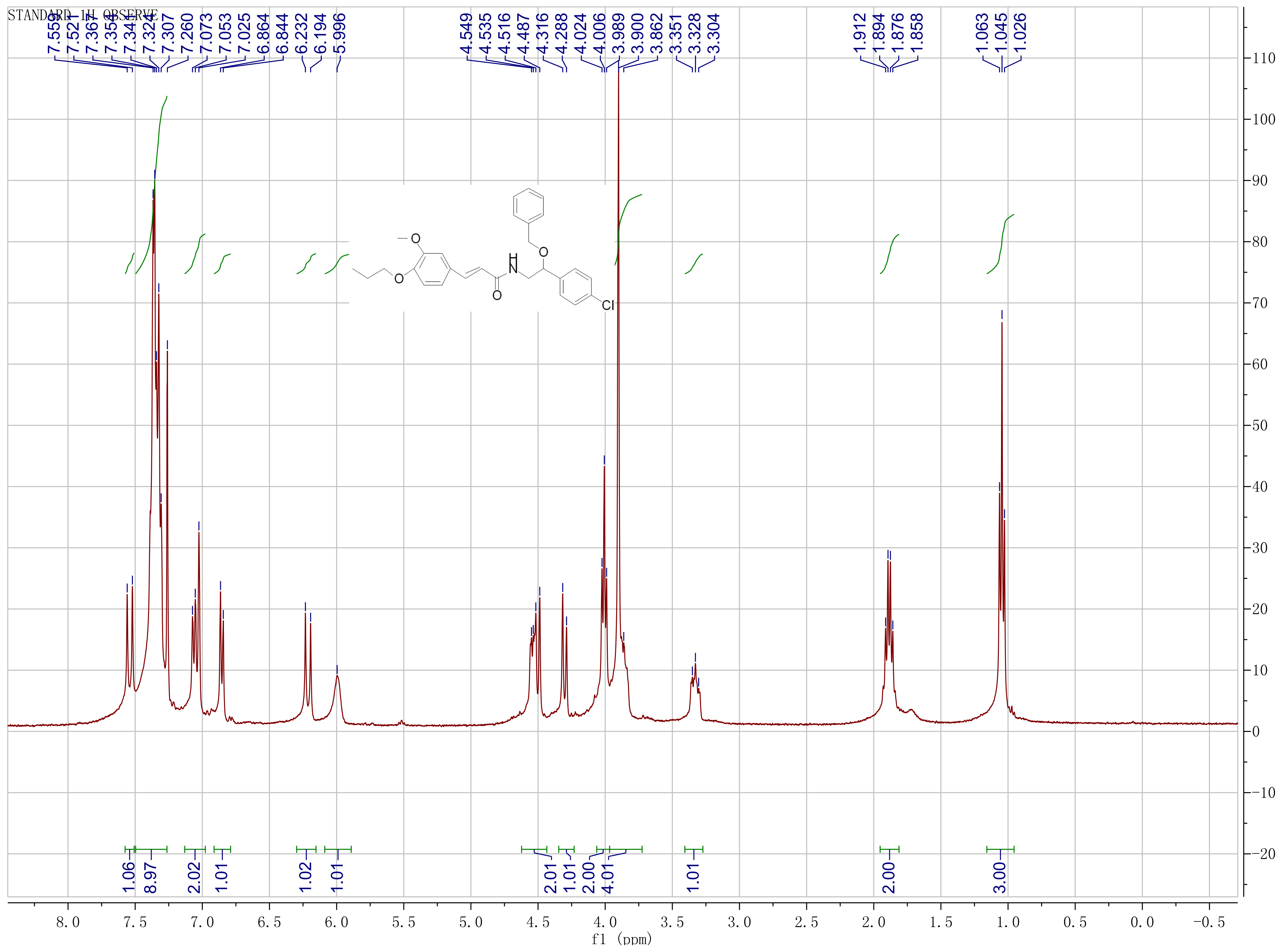
STANDARD 1H OBSERVE
7.521
7.361
7.354
7.341
7.324
7.307
7.260
7.073
7.053
7.025
6.864
6.844
6.232
6.194
5.996
4.549
4.535
4.516
4.487
4.316
4.288
4.024
4.006
3.989
3.900
3.862
3.351
3.328
3.304
1.912
1.894
1.876
1.858
1.063
1.045
1.026
1.06
8.97
2.02
1.01
1.02
1.01
2.01
1.01
2.00
4.01
1.01
2.00
3.00
O
O
H
N
O
O
Cl
f1 (ppm)

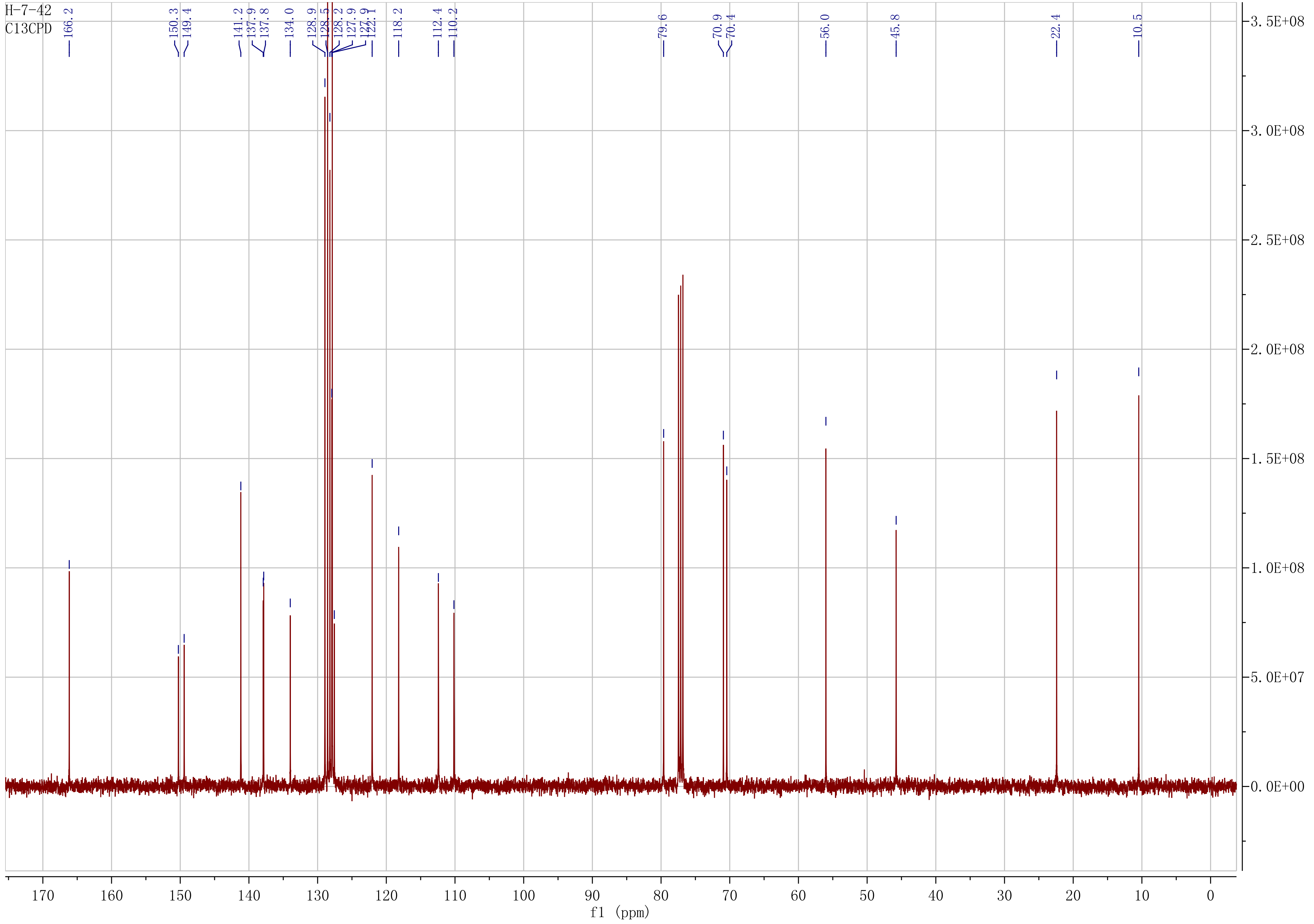
H-7-42
C13CPD
166.2
150.3
149.4
141.2
137.9
137.8
134.0
128.9
128.5
128.2
127.9
127.9
122.1
118.2
112.4
110.2
79.6
70.9
70.4
56.0
45.8
22.4
10.5
3.5E+08
3.0E+08
2.5E+08
2.0E+08
1.5E+08
1.0E+08
5.0E+07
0.0E+00
170
160
150
140
130
120
110
100
90
80
70
60
50
40
30
20
10
0
f1 (ppm)

Varian ProMALDI
File: h-7-42_MALDI.trans

Mode: Positive
Scans: 1

Date: 29-MAR-200
Time: 20:12:00
Scale: 111.4903

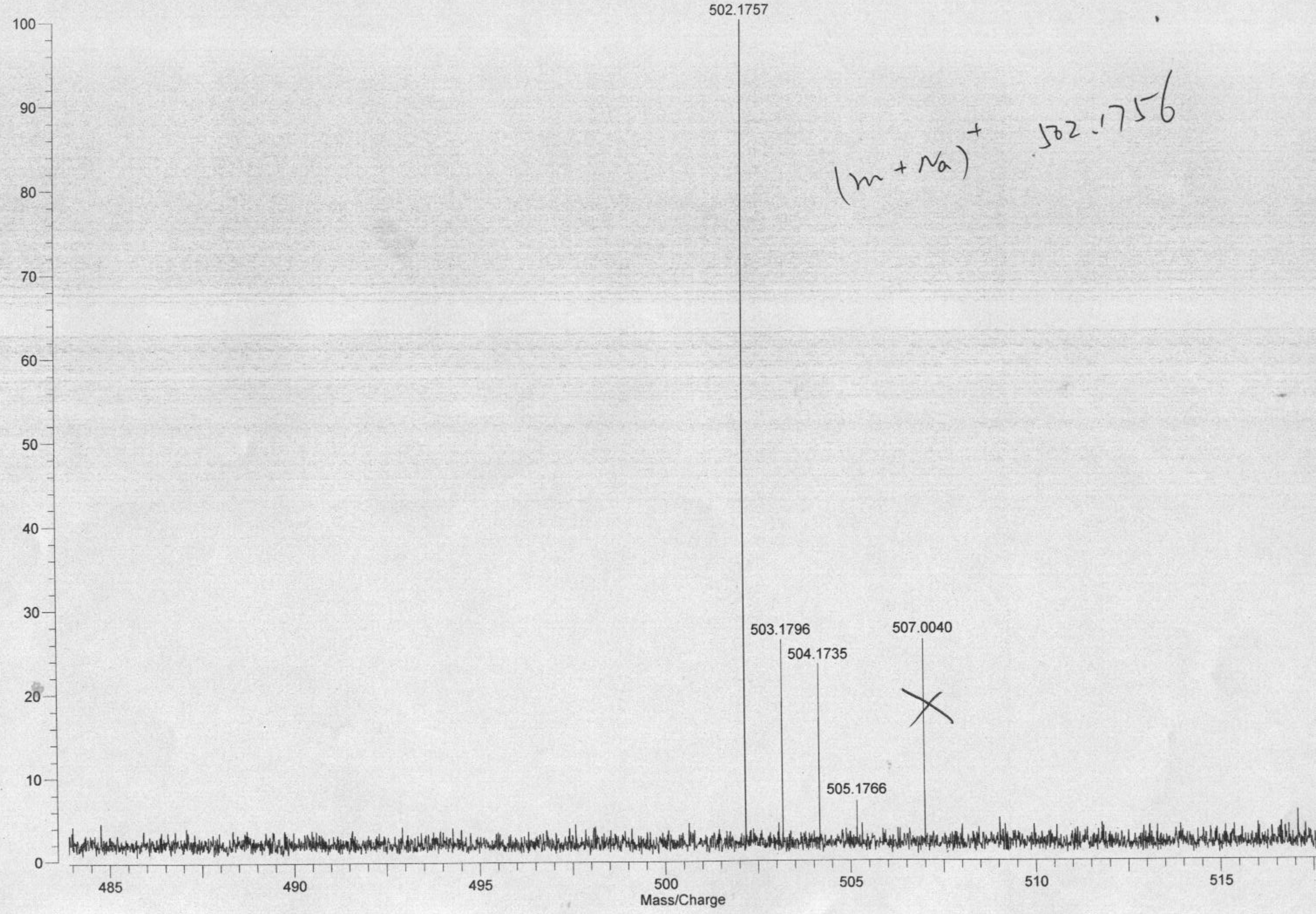

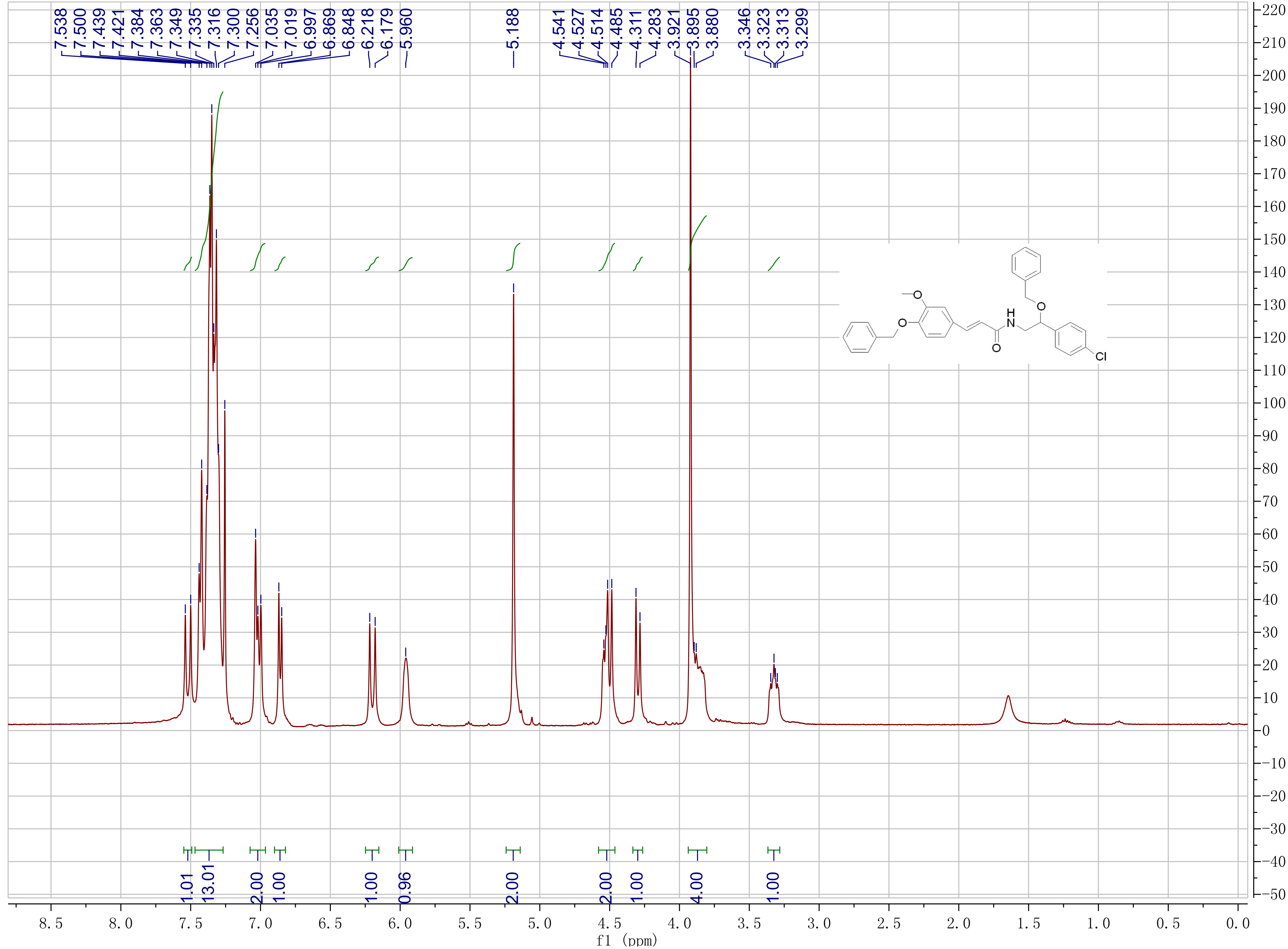

7.538
7.500
7.439
7.421
7.384
7.363
7.349
7.335
7.316
7.300
7.256
7.035
7.019
6.997
6.869
6.848
6.218
6.179
5.960
5.188
4.541
4.527
4.514
4.485
4.311
4.283
3.921
3.895
3.880
3.346
3.323
3.313
3.299
1.01
13.01
2.00
1.00
1.00
0.96
2.00
2.00
1.00
4.00
1.00
f1 (ppm)
O
H
N
Cl

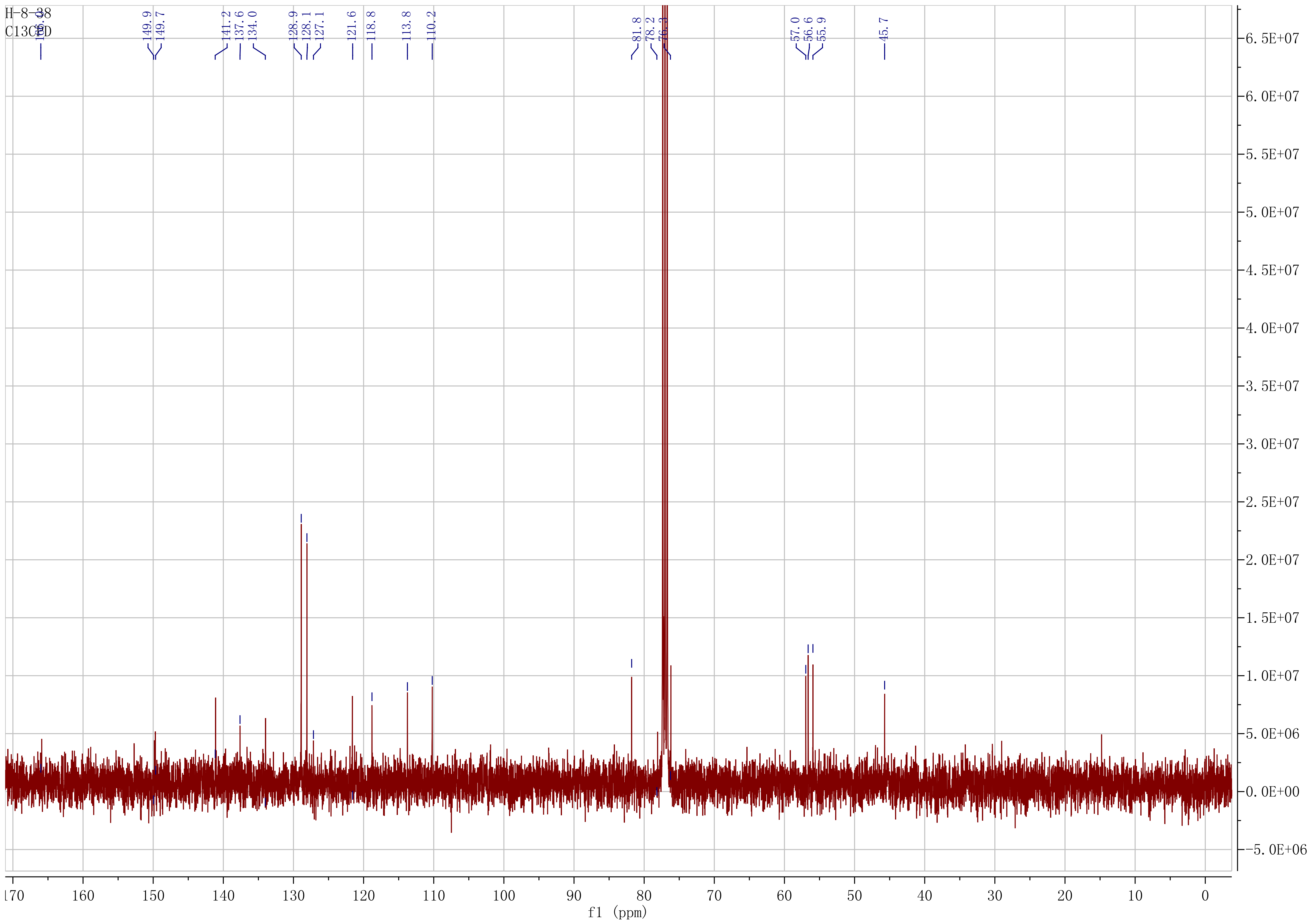
H-8-38
C13CPD
166.0
149.9
149.7
141.2
137.6
134.0
128.9
128.1
127.1
121.6
118.8
113.8
110.2
81.8
78.2
76.3
57.0
56.6
55.9
45.7
f1 (ppm)

| | | | | | | | |
|---|---|---|---|---|---|---|---|
| **Sample Name** | lc/ms | **Position** | Vial 3 | **Instrument Name** | Instrument 1 | **User Name** | |
| **Inj Vol** | -1 | **InjPosition** | | **SampleType** | Sample | **IRM Calibration Status** | Some Ions Missed |
| **Data Filename** | H-8-38.d | **ACQ Method** | | **Comment** | | **Acquired Time** | 4/9/2009 8:31:16 AM |

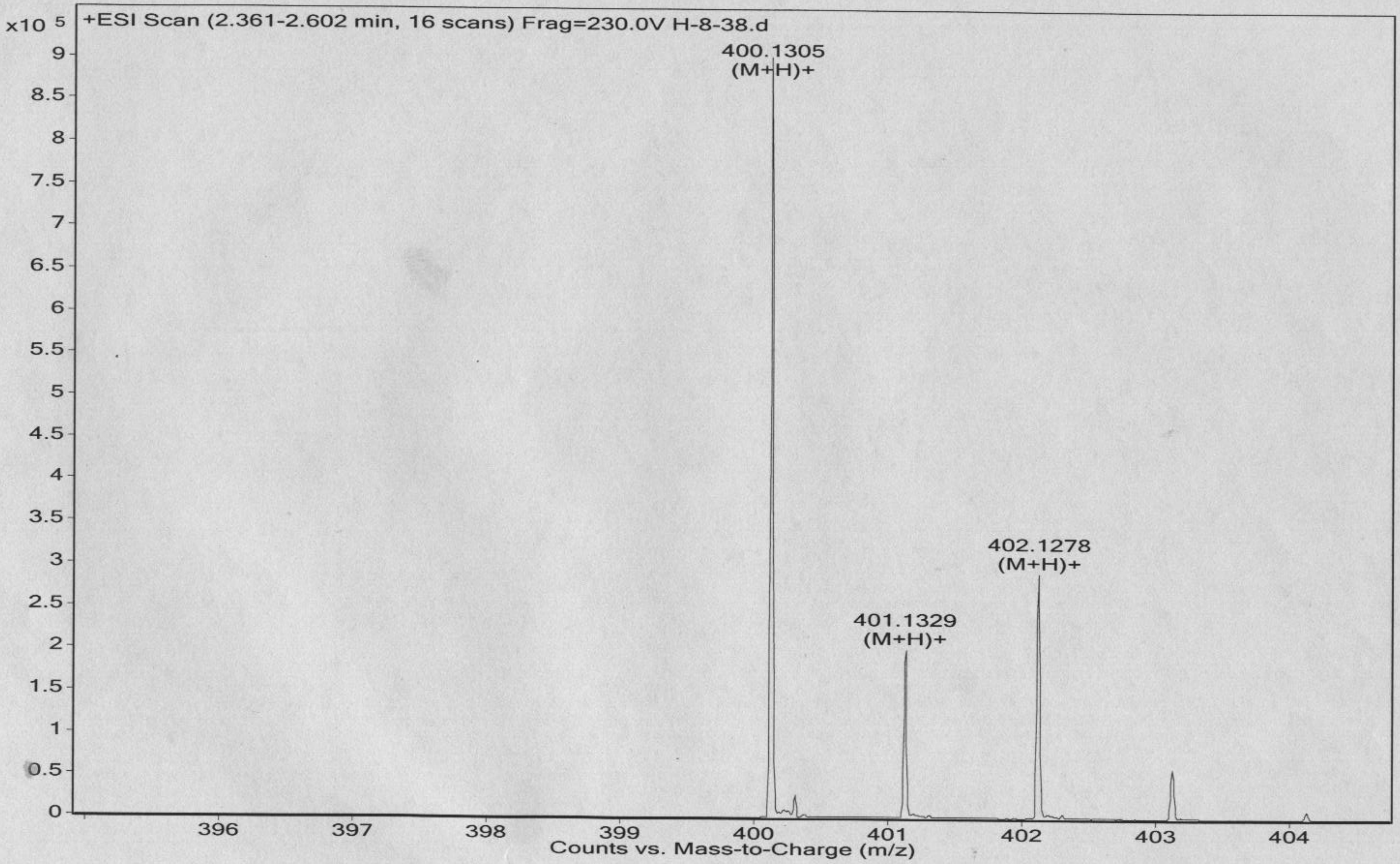

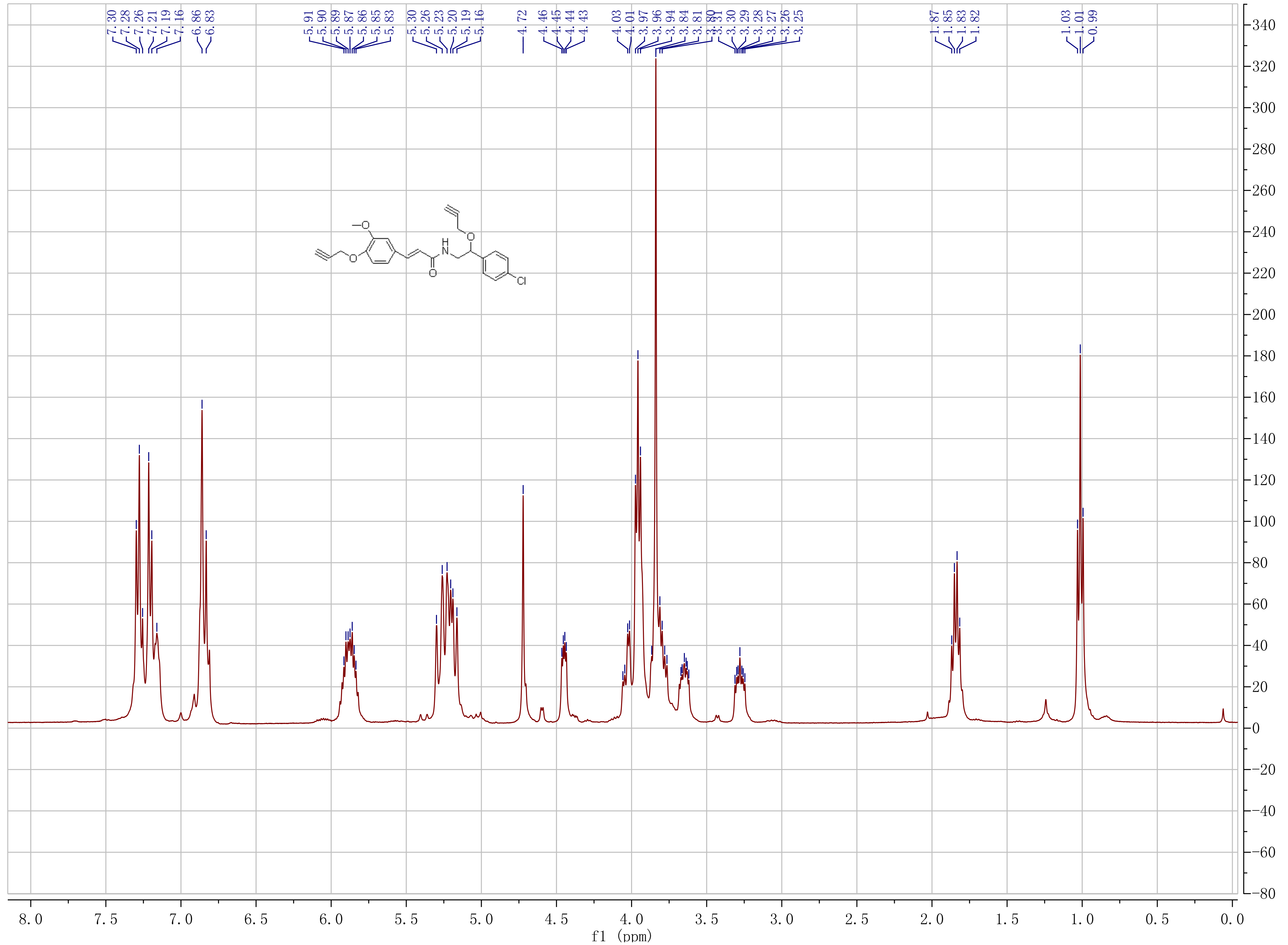
7.30
7.28
7.26
7.21
7.19
7.16
6.86
6.83
5.91
5.90
5.89
5.87
5.86
5.85
5.83
5.30
5.26
5.23
5.20
5.19
5.16
4.72
4.46
4.45
4.44
4.43
4.03
4.01
3.97
3.96
3.94
3.84
3.81
3.80
3.31
3.30
3.29
3.28
3.27
3.26
3.25
1.87
1.85
1.83
1.82
1.03
1.01
0.99
O
O
H
N
O
O
Cl
f1 (ppm)

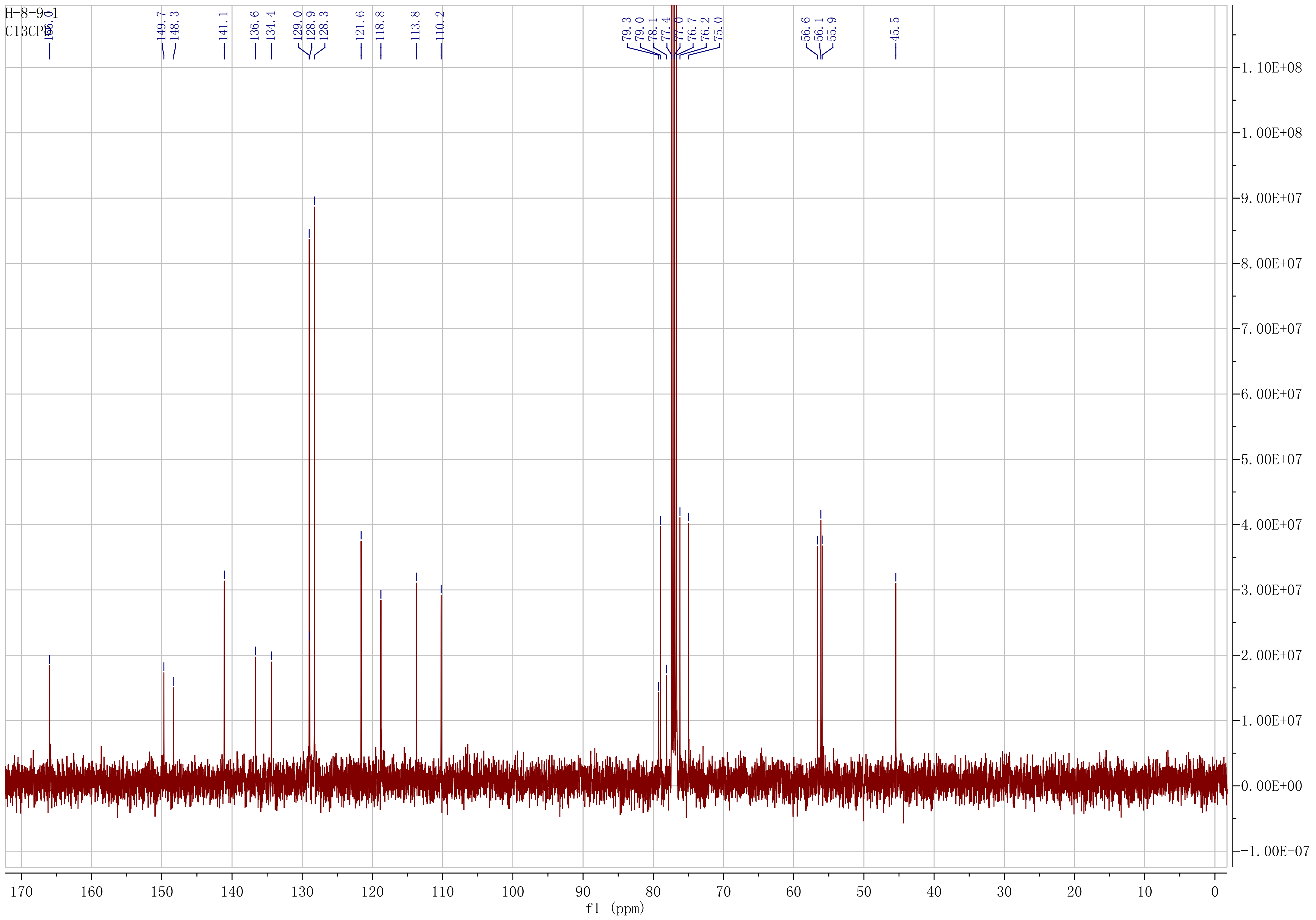
H-8-9-1
C13CPD
166.0
149.7
148.3
141.1
136.6
134.4
129.0
128.9
128.3
121.6
118.8
113.8
110.2
79.3
79.0
78.1
77.4
77.0
76.7
76.2
75.0
56.6
56.1
55.9
45.5
1.10E+08
1.00E+08
9.00E+07
8.00E+07
7.00E+07
6.00E+07
5.00E+07
4.00E+07
3.00E+07
2.00E+07
1.00E+07
0.00E+00
-1.00E+07
170
160
150
140
130
120
110
100
90
80
70
60
50
40
30
20
10
0
f1 (ppm)

Varian ProMALDI
File: h-8-9-1_MALDI.trans

Mode: Positive
Scans: 1

Date: 30-MAR-200
Time: 08:53:36
Scale: 62.4709

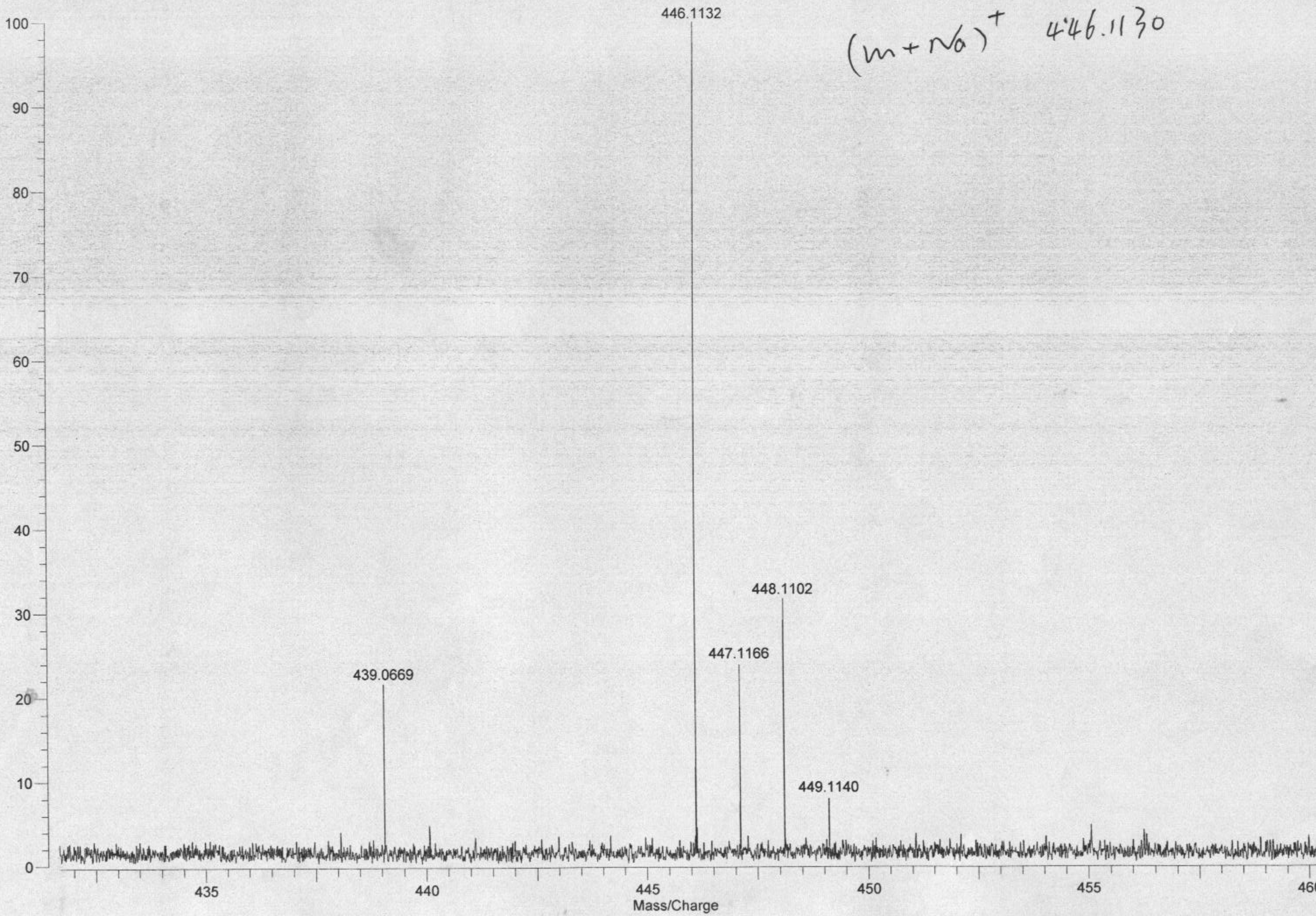

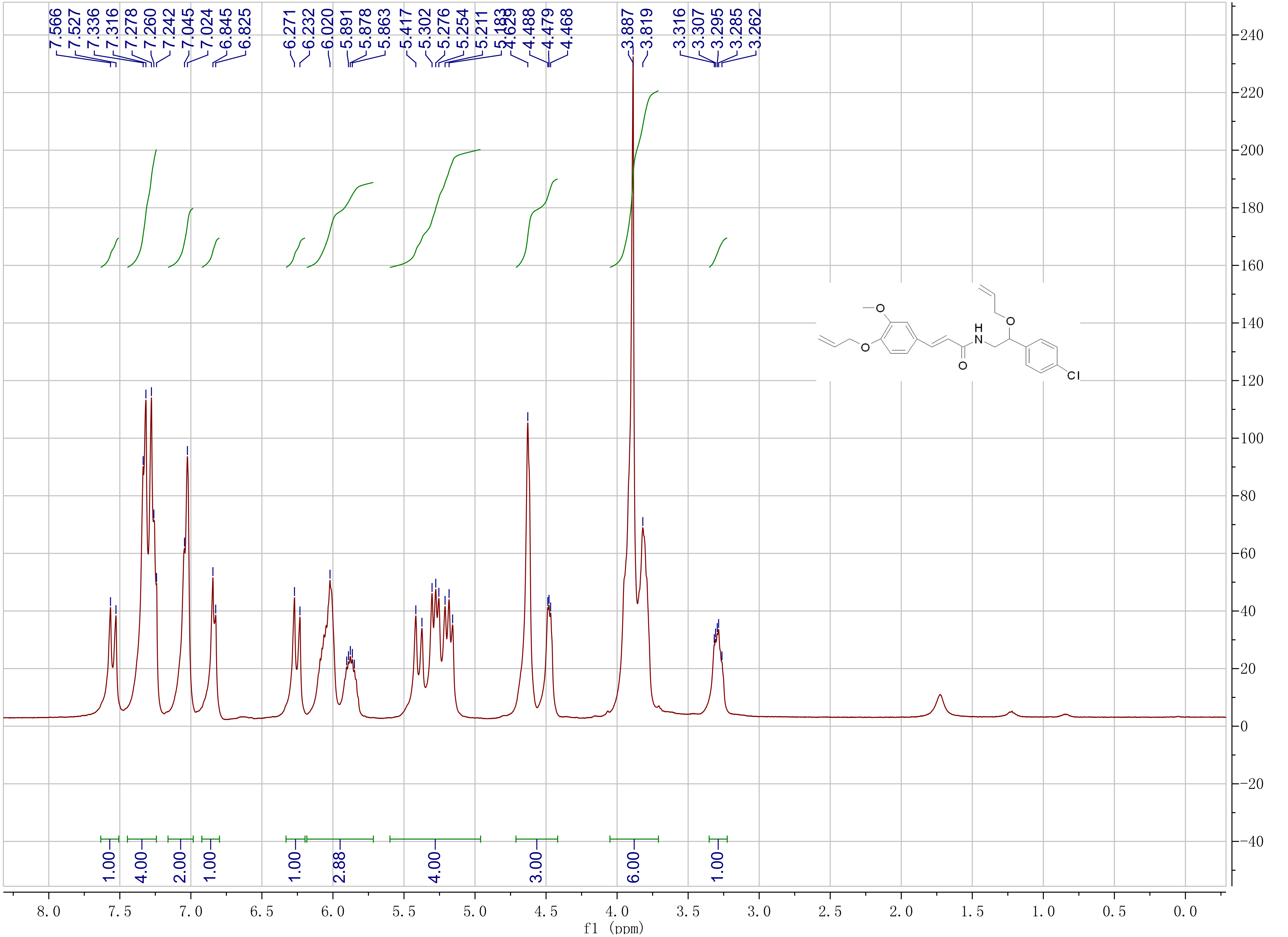

7.566
7.527
7.336
7.316
7.278
7.260
7.242
7.045
7.024
6.845
6.825
6.271
6.232
6.020
5.891
5.878
5.863
5.417
5.302
5.276
5.254
5.211
5.183
4.629
4.488
4.479
4.468
3.887
3.819
3.316
3.307
3.295
3.285
3.262
1.00
4.00
2.00
1.00
1.00
2.88
4.00
3.00
6.00
1.00
O
H
N
O
O
O
Cl
f1 (ppm)

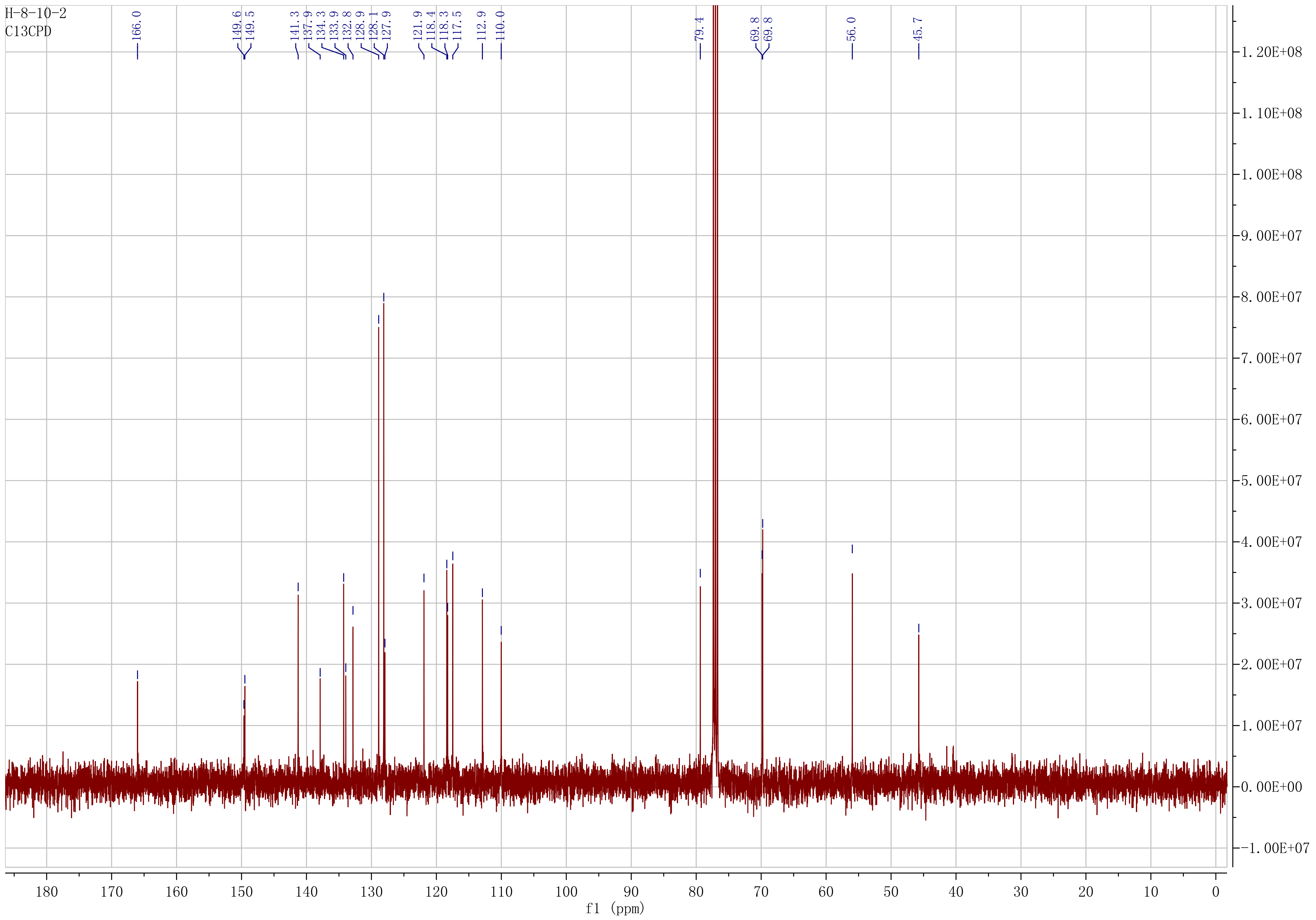
H-8-10-2
C13CPD
166.0
149.6
149.5
141.3
137.9
134.3
133.9
132.8
128.9
128.1
127.9
121.9
118.4
118.3
117.5
112.9
110.0
79.4
69.8
69.8
56.0
45.7
f1 (ppm)

Varian ProMALDI
File: h-8-10-2_MALDI.trans

Mode: Positive
Scans: 1

Date: 29-MAR-200
Time: 21:19:48
Scale: 145.8876

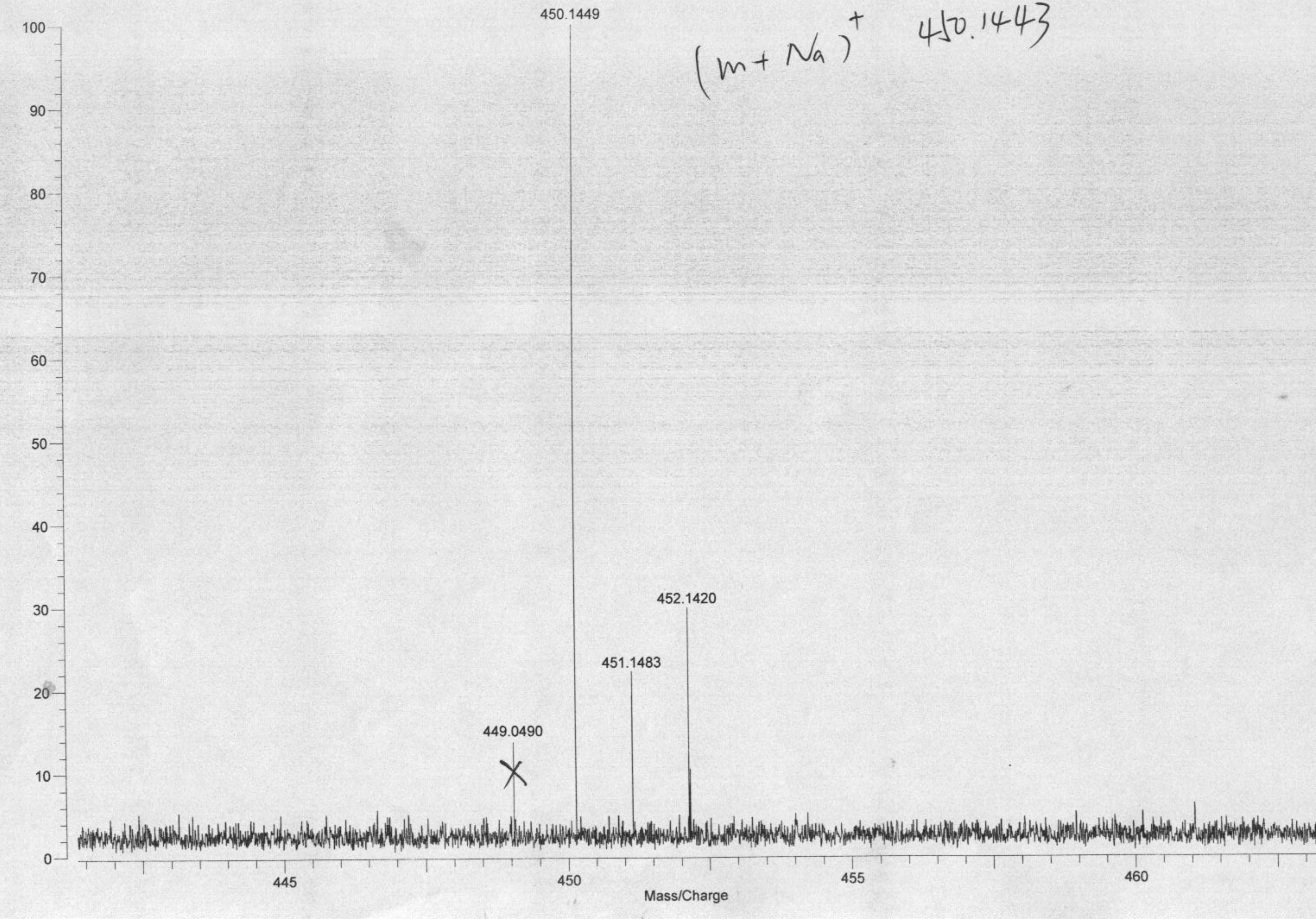

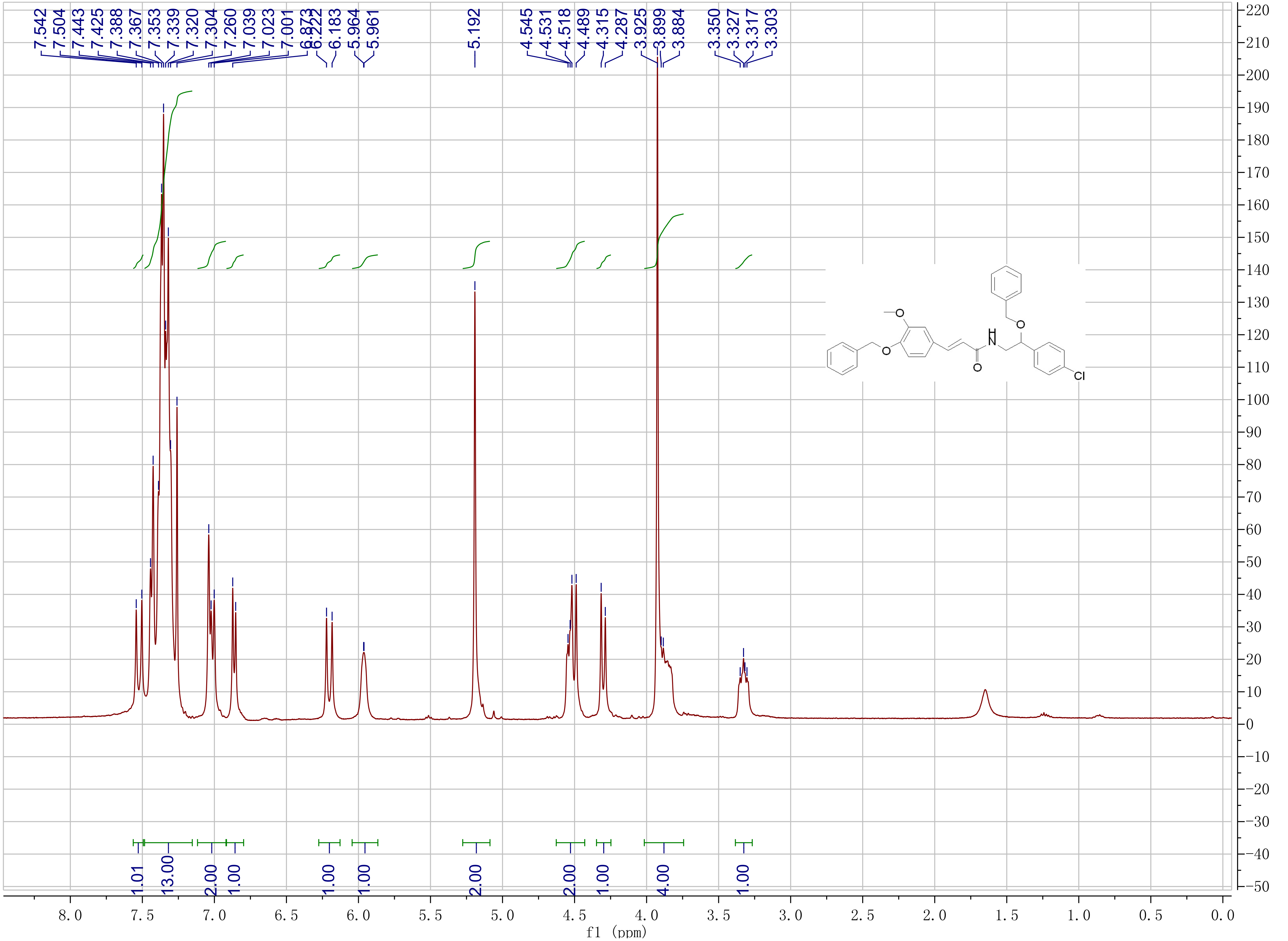

7.542
7.504
7.443
7.425
7.388
7.367
7.353
7.339
7.320
7.304
7.260
7.039
7.023
7.001
6.873
6.222
6.183
5.964
5.961
5.192
4.545
4.531
4.518
4.489
4.315
4.287
3.925
3.899
3.884
3.350
3.327
3.317
3.303
1.01
13.00
2.00
1.00
1.00
1.00
2.00
2.00
1.00
4.00
1.00
O
O
H
N
O
O
Cl
f1 (ppm)

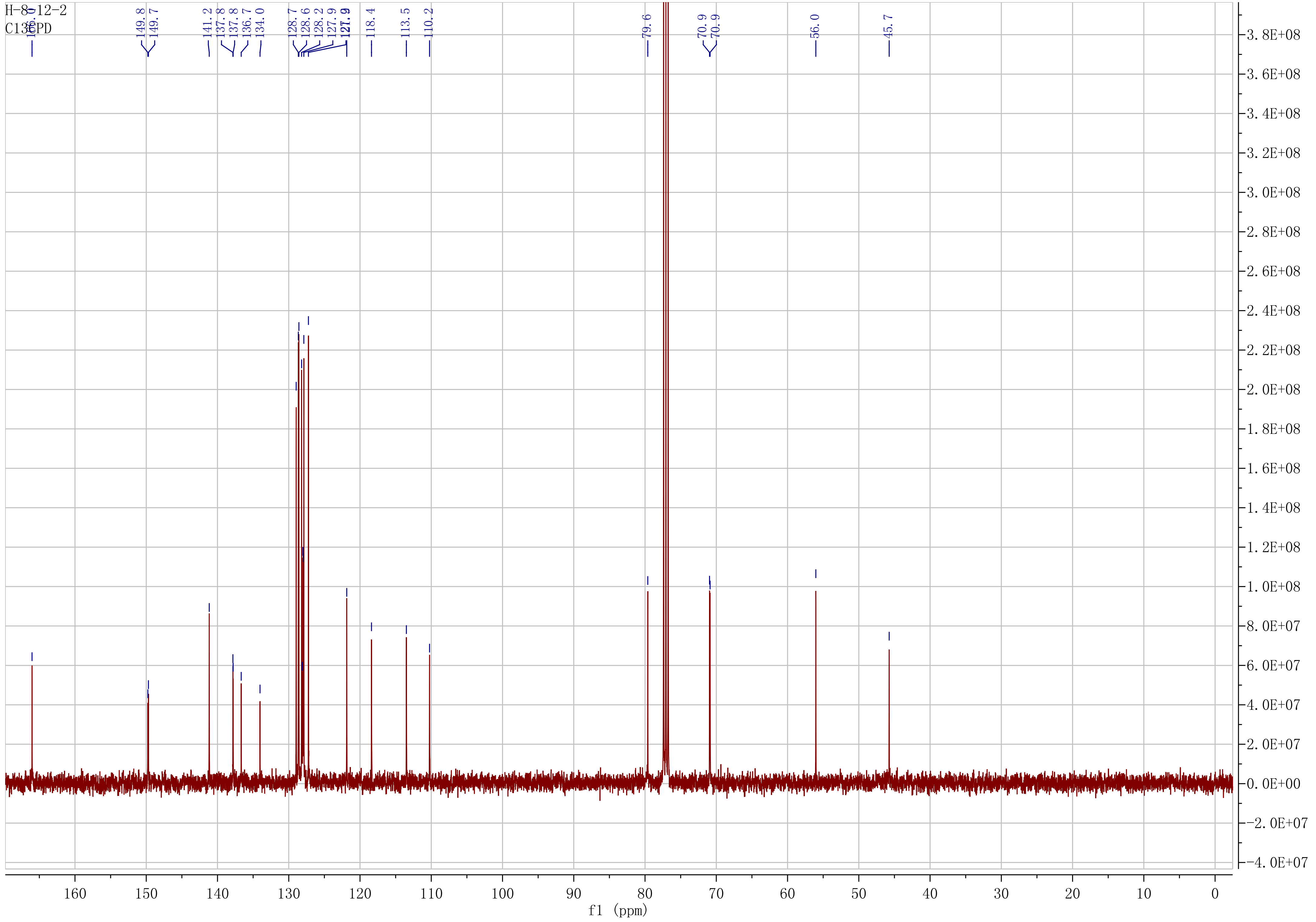

H-8-12-2
C13CPD
166.0
149.8
149.7
141.2
137.8
137.8
136.7
134.0
128.7
128.6
128.2
127.9
118.4
113.5
110.2
79.6
70.9
70.9
56.0
45.7
f1 (ppm)

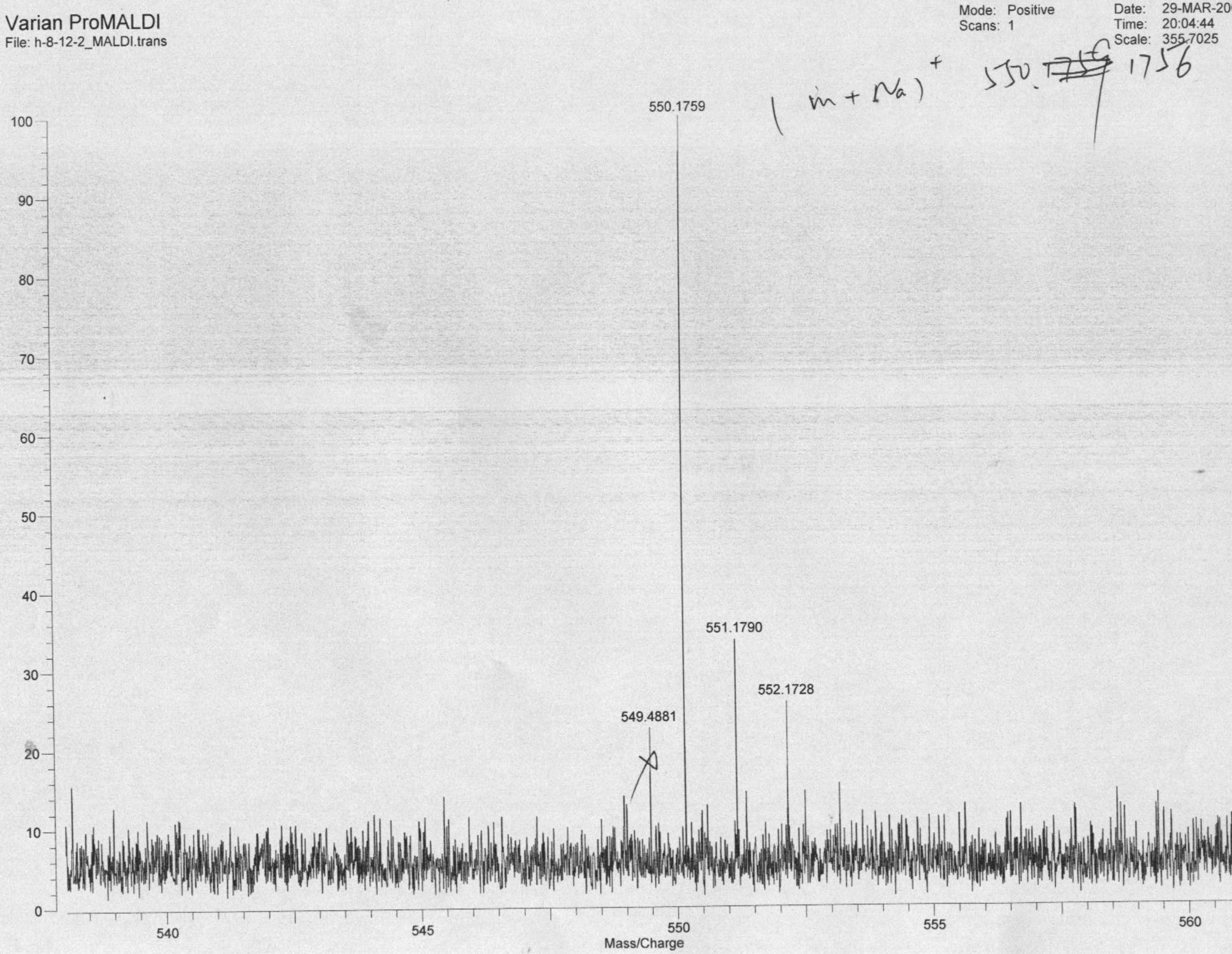
Varian ProMALDI
File: h-8-12-2_MALDI.trans
Mode: Positive
Scans: 1
Date: 29-MAR-20
Time: 20:04:44
Scale: 355.7025
(m + Na)+ 550.1756
550.1759
551.1790
552.1728
549.4881
100
90
80
70
60
50
40
30
20
10
0
540
545
550
555
560
Mass/Charge

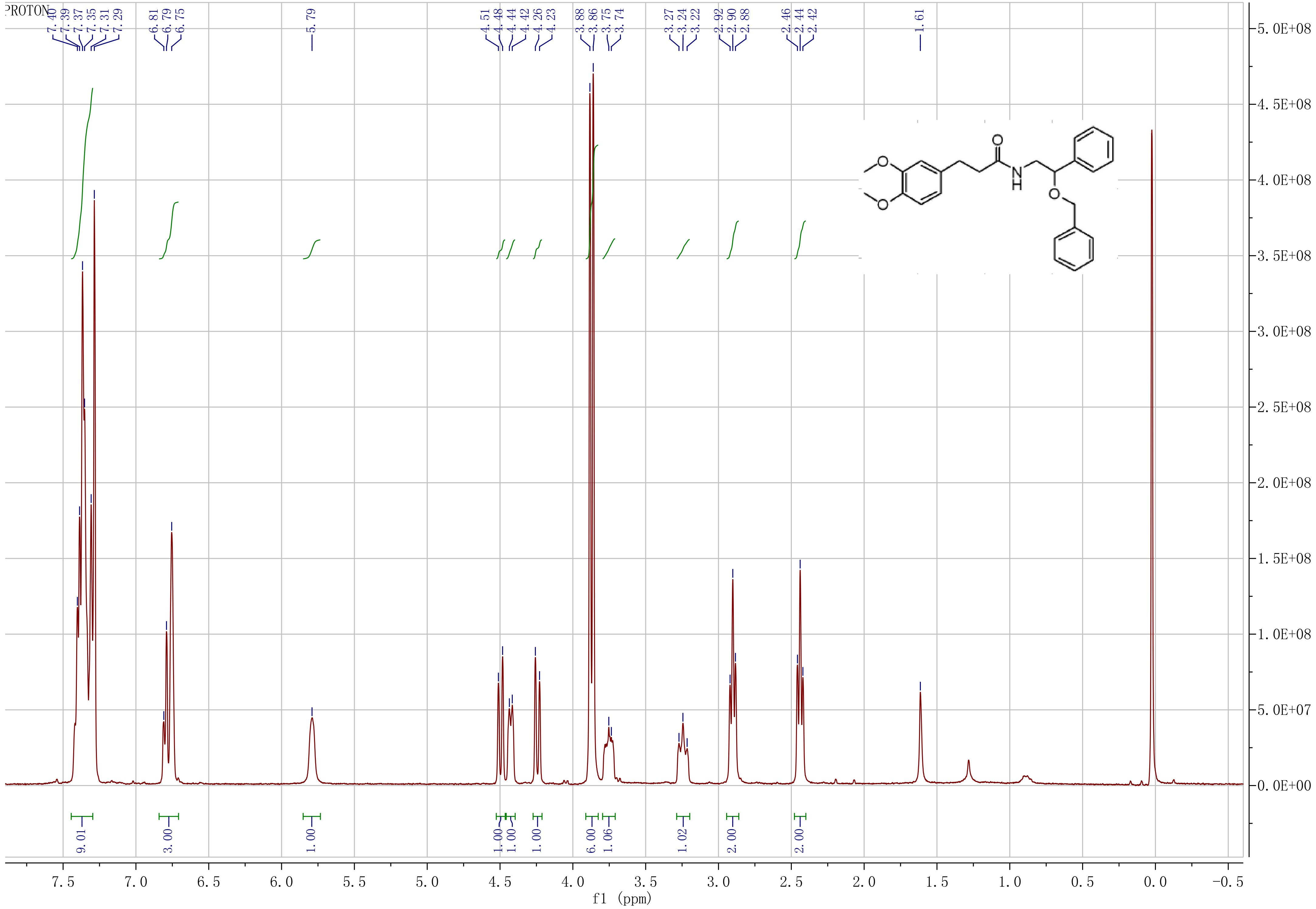
PROTON
7.40 7.39 7.37 7.35 7.31 7.29
6.81 6.79 6.75
5.79
4.51 4.48 4.44 4.42 4.26 4.23
3.88 3.86 3.75 3.74
3.27 3.24 3.22
2.92 2.90 2.88
2.46 2.44 2.42
1.61
9.01
3.00
1.00
1.00
1.00
1.00
6.00
1.06
1.02
2.00
2.00
O
N
H
O
O
O
5.0E+08
4.5E+08
4.0E+08
3.5E+08
3.0E+08
2.5E+08
2.0E+08
1.5E+08
1.0E+08
5.0E+07
0.0E+00
7.5 7.0 6.5 6.0 5.5 5.0 4.5 4.0 3.5 3.0 2.5 2.0 1.5 1.0 0.5 0.0 -0.5
f1 (ppm)

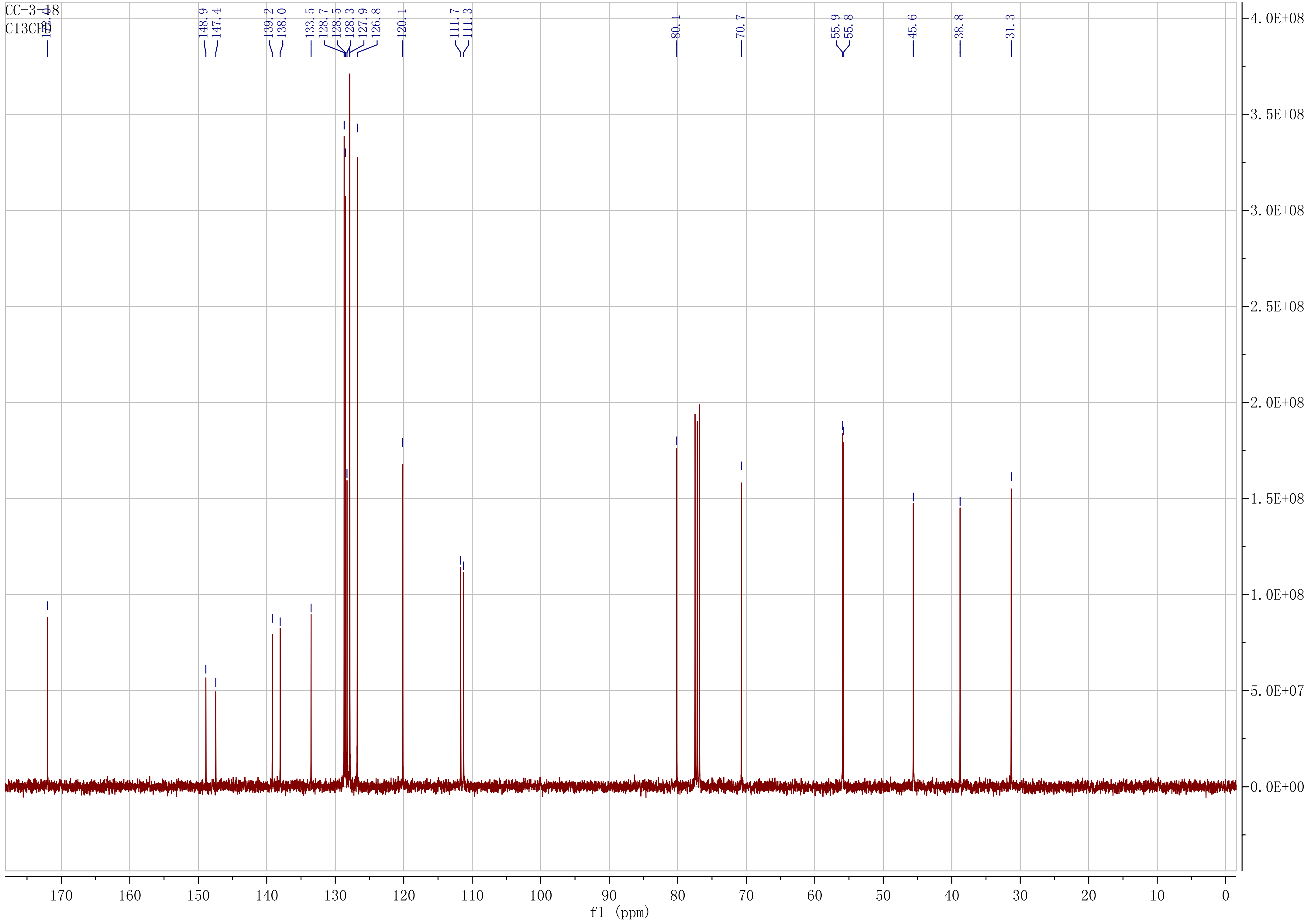
CC-3-18
C13CPD
172.0
148.9
147.4
139.2
138.0
133.5
128.7
128.5
128.3
127.9
126.8
120.1
111.7
111.3
80.1
70.7
55.9
55.8
45.6
38.8
31.3
4.0E+08
3.5E+08
3.0E+08
2.5E+08
2.0E+08
1.5E+08
1.0E+08
5.0E+07
0.0E+00
170
160
150
140
130
120
110
100
90
80
70
60
50
40
30
20
10
0
f1 (ppm)

Varian ProMALDI
File: cc-3-18_MALDI.trans

Mode: Positive
Scans: 1

Date: 01-JU[illegible]
Time: 17:52[illegible]
Scale: 2.250[illegible]

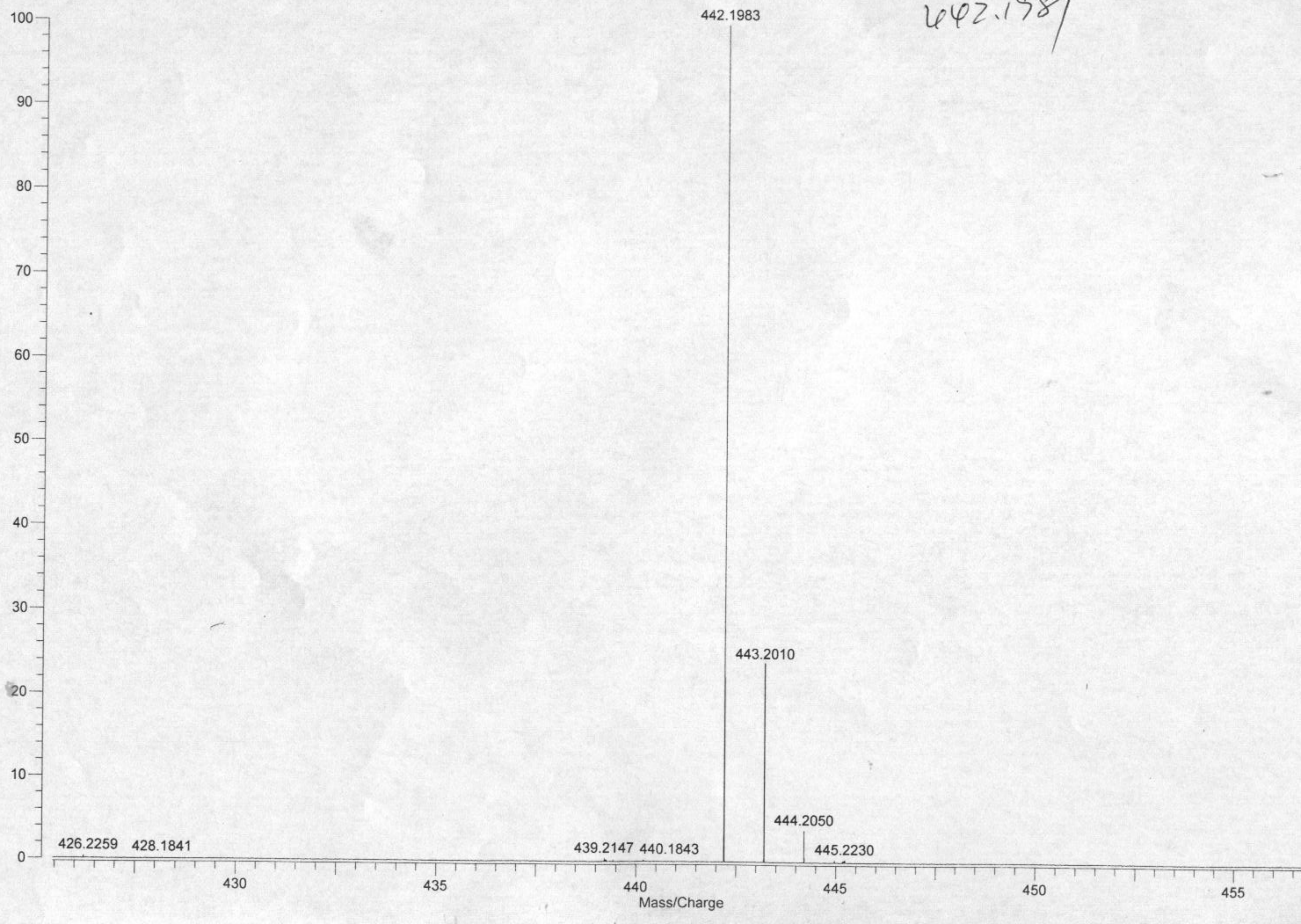

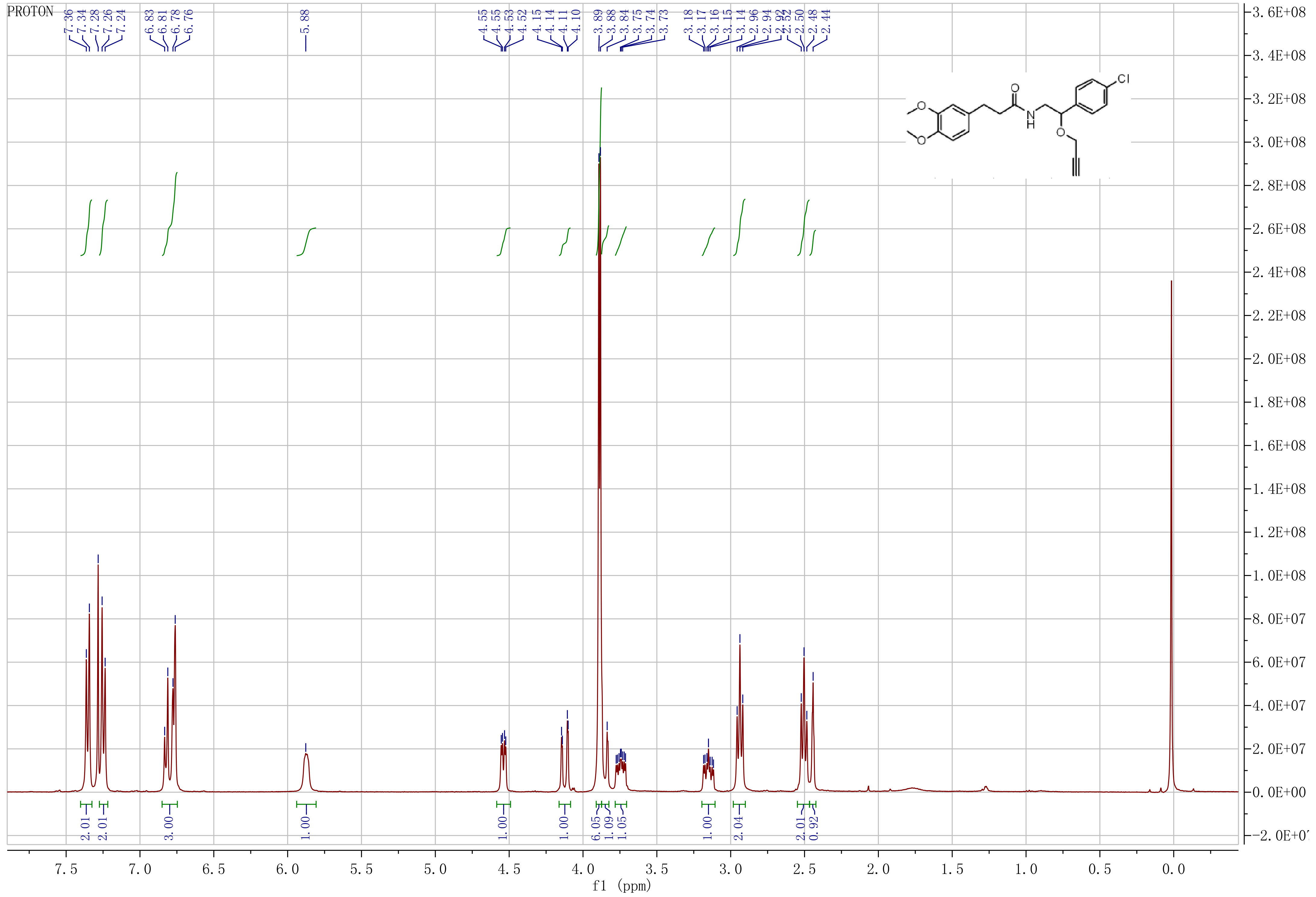
PROTON
7.36 7.34 7.28 7.26 7.24
6.83 6.81 6.78 6.76
5.88
4.55 4.55 4.53 4.52 4.15 4.14 4.11 4.10
3.89 3.88 3.84 3.75 3.74 3.73
3.18 3.17 3.16 3.15 3.14 2.96 2.94 2.92 2.52 2.50 2.48 2.44
2.01 2.01 3.00 1.00 1.00 1.00 6.05 1.09 1.05 1.00 2.04 2.01 0.92
Cl
O
N
H
O
O
O
f1 (ppm)

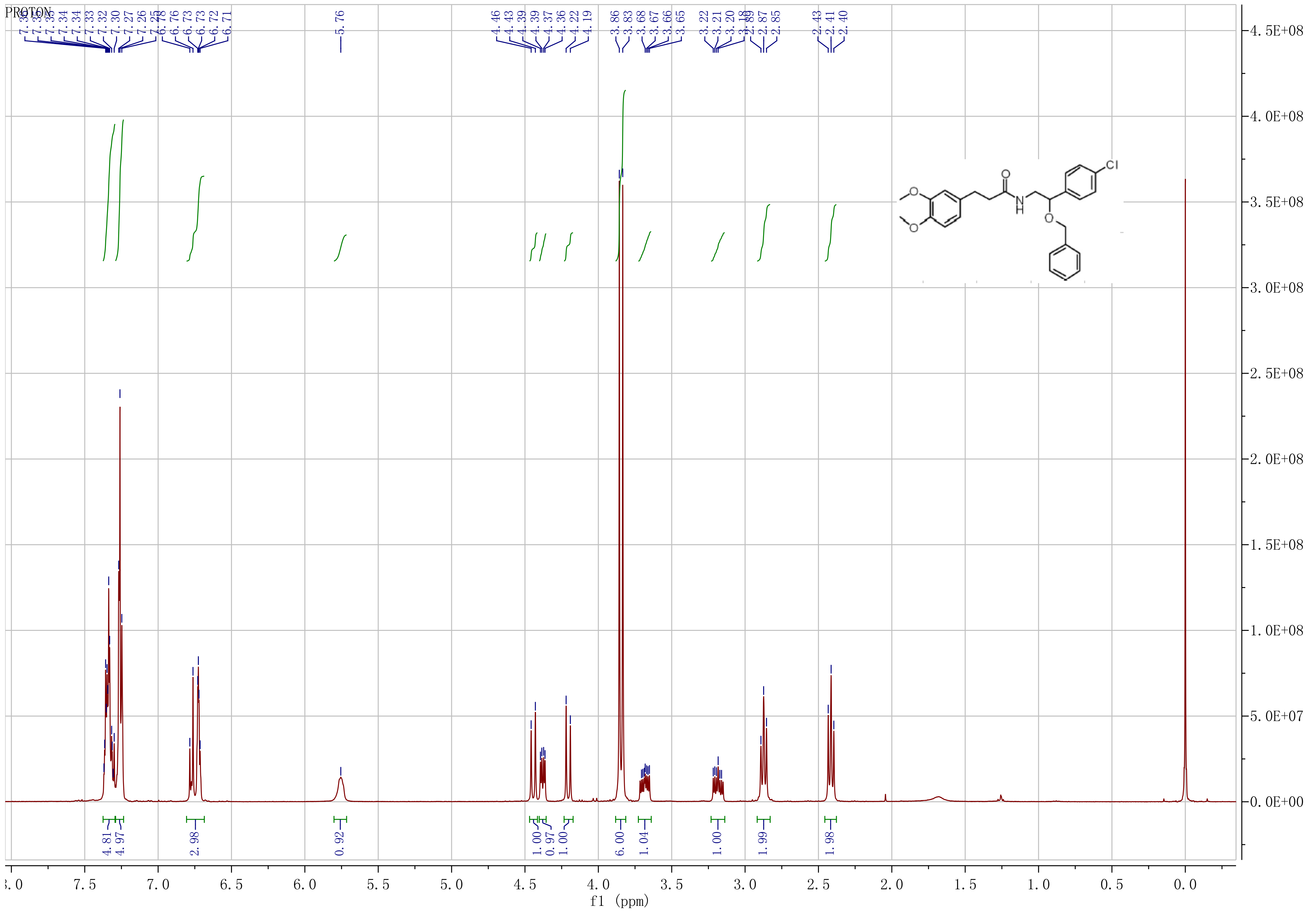
PROTON
5.76
f1 (ppm)
Cl
O
N
H

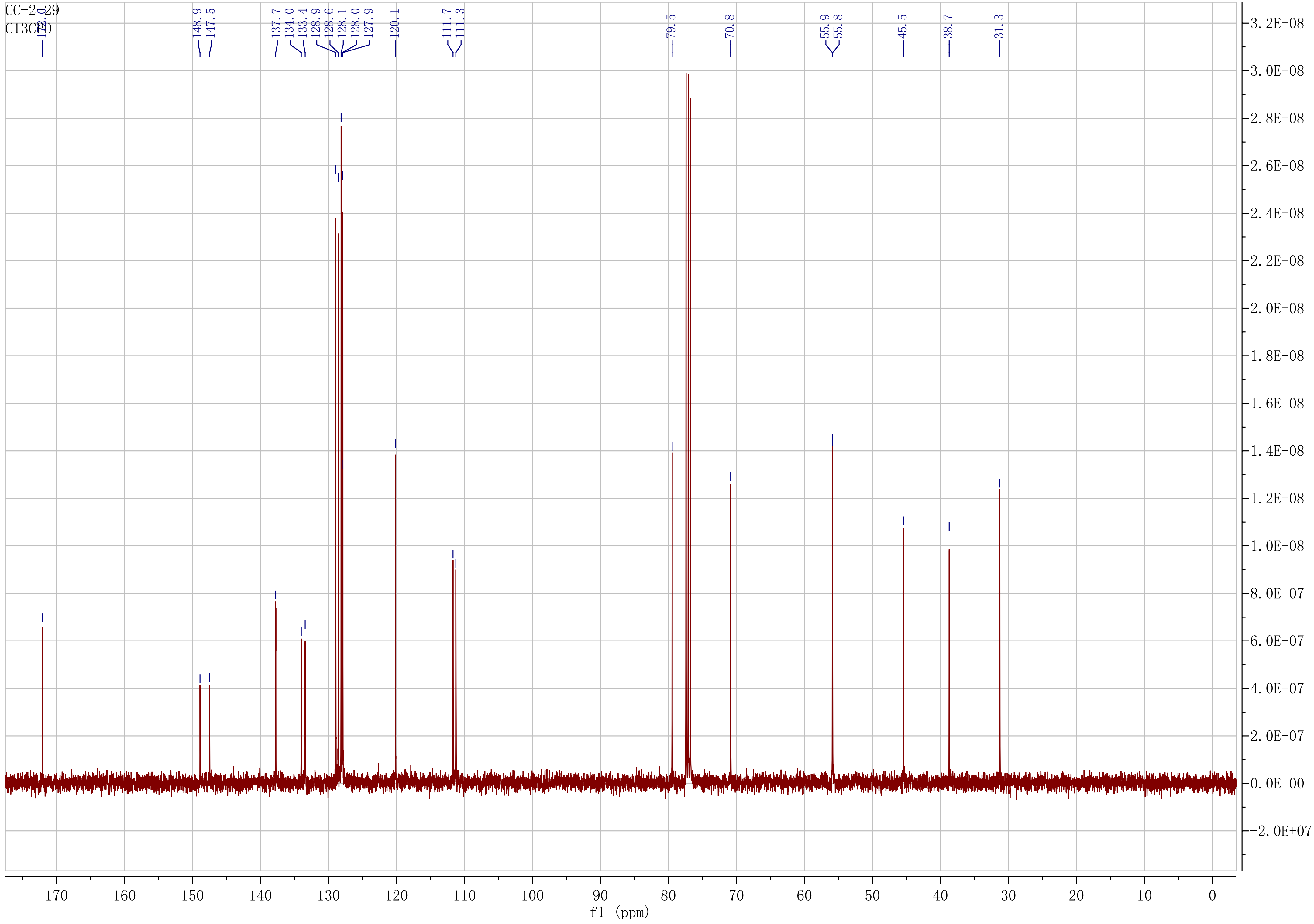
CC-2-29
C13CPD
172.0
148.9
147.5
137.7
134.0
133.4
128.9
128.6
128.1
128.0
127.9
120.1
111.7
111.3
79.5
70.8
55.9
55.8
45.5
38.7
31.3
3.2E+08
3.0E+08
2.8E+08
2.6E+08
2.4E+08
2.2E+08
2.0E+08
1.8E+08
1.6E+08
1.4E+08
1.2E+08
1.0E+08
8.0E+07
6.0E+07
4.0E+07
2.0E+07
0.0E+00
-2.0E+07
170
160
150
140
130
120
110
100
90
80
70
60
50
40
30
20
10
0
f1 (ppm)

18R

Varian ProMALDI
File: cc-2-31_MALDI.trans

Mode: Positive
Scans: 1

Date: 01-JUN-2011
Time: 17:56:00
Scale: 5.6266

476.1599

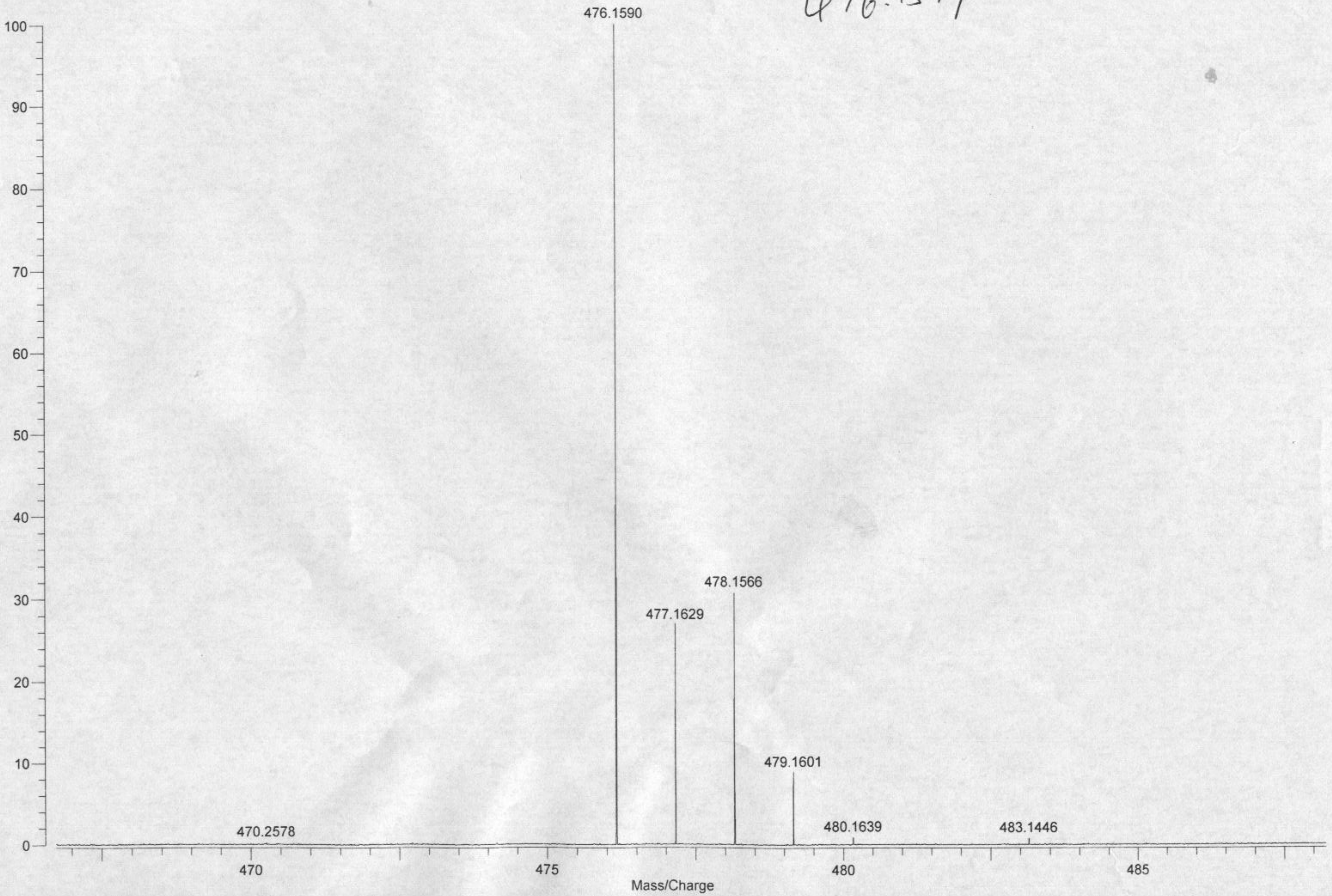

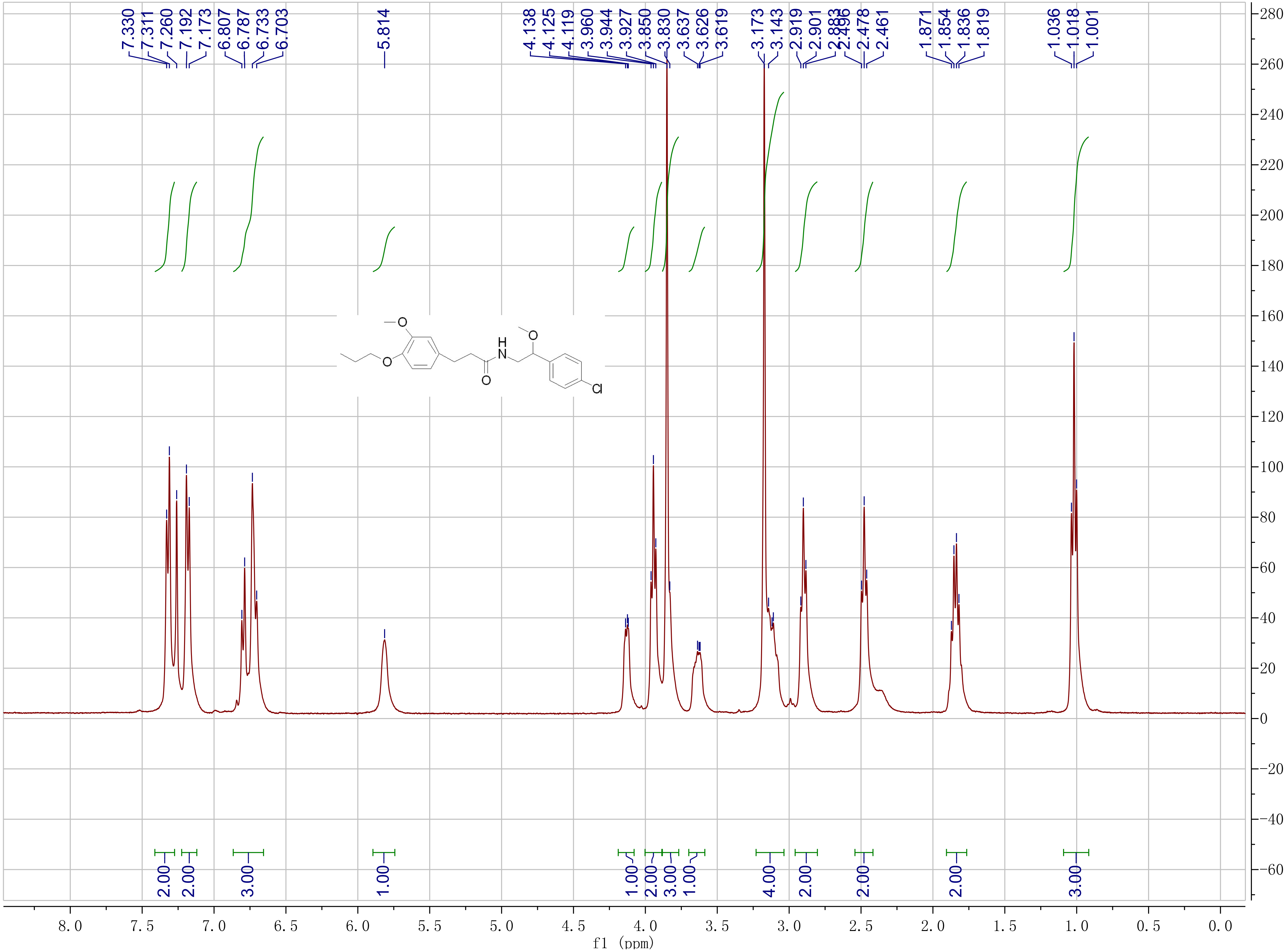

7.330
7.311
7.260
7.192
7.173
6.807
6.787
6.733
6.703
5.814
4.138
4.125
4.119
3.960
3.944
3.927
3.850
3.830
3.637
3.626
3.619
3.173
3.143
2.919
2.901
2.883
2.496
2.478
2.461
1.871
1.854
1.836
1.819
1.036
1.018
1.001
2.00
2.00
3.00
1.00
1.00
2.00
3.00
1.00
4.00
2.00
2.00
2.00
3.00
O
H
N
O
O
O
Cl
f1 (ppm)

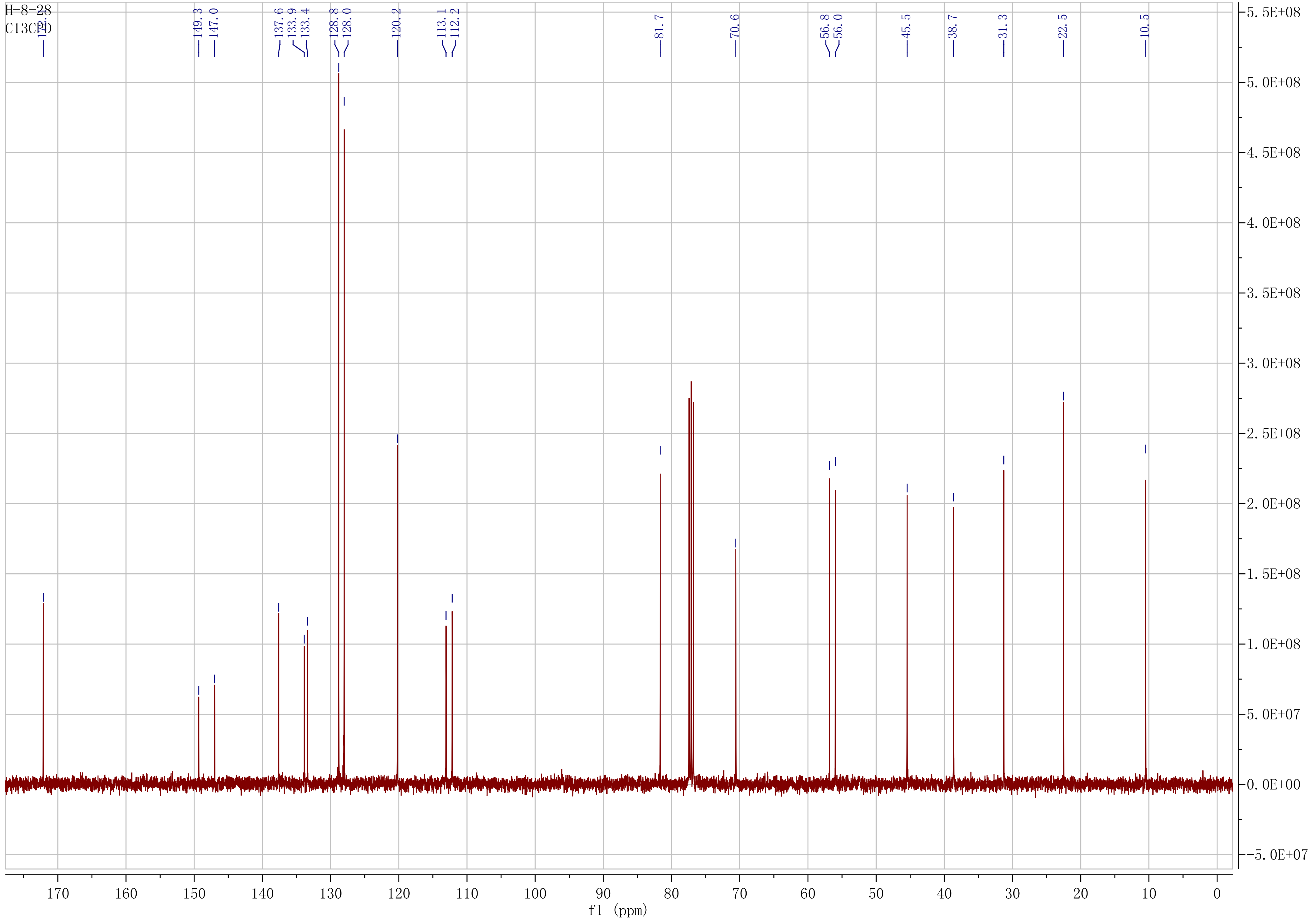
H-8-28
C13CPD
172.
149.3
147.0
137.6
133.9
133.4
128.8
128.0
120.2
113.1
112.2
81.7
70.6
56.8
56.0
45.5
38.7
31.3
22.5
10.5
5.5E+08
5.0E+08
4.5E+08
4.0E+08
3.5E+08
3.0E+08
2.5E+08
2.0E+08
1.5E+08
1.0E+08
5.0E+07
0.0E+00
-5.0E+07
170
160
150
140
130
120
110
100
90
80
70
60
50
40
30
20
10
0
f1 (ppm)

| | | | | | | | |
|---|---|---|---|---|---|---|---|
| **Sample Name** | lc/ms | **Position** | P1-A1 | **Instrument Name** | Instrument 1 | **User Name** | |
| **Inj Vol** | -1 | **InjPosition** | | **SampleType** | Sample | **IRM Calibration Status** | Some Ions Missed |
| **Data Filename** | H-8-28.d | **ACQ Method** | | **Comment** | | **Acquired Time** | 4/9/2009 9:00:10 AM |

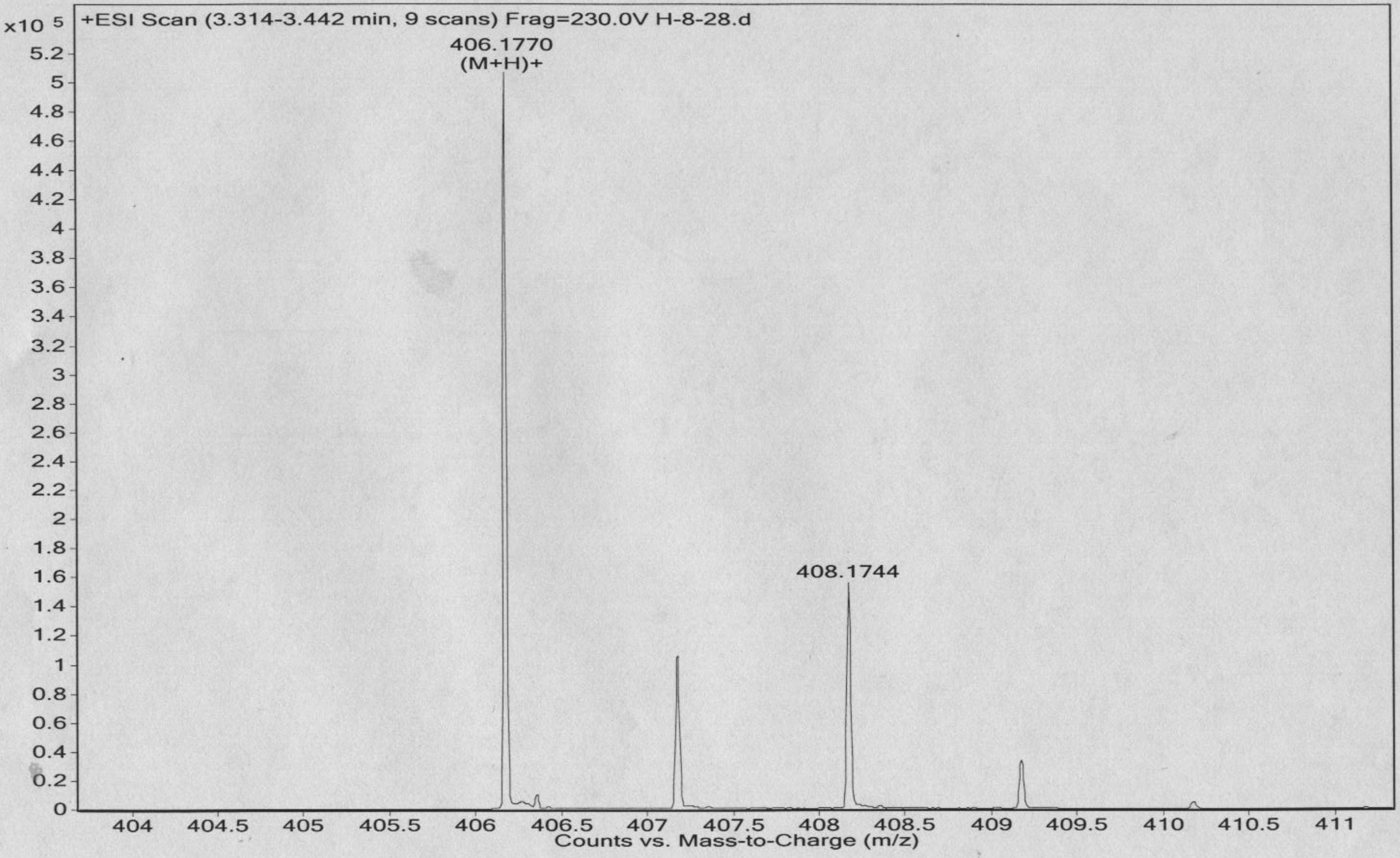

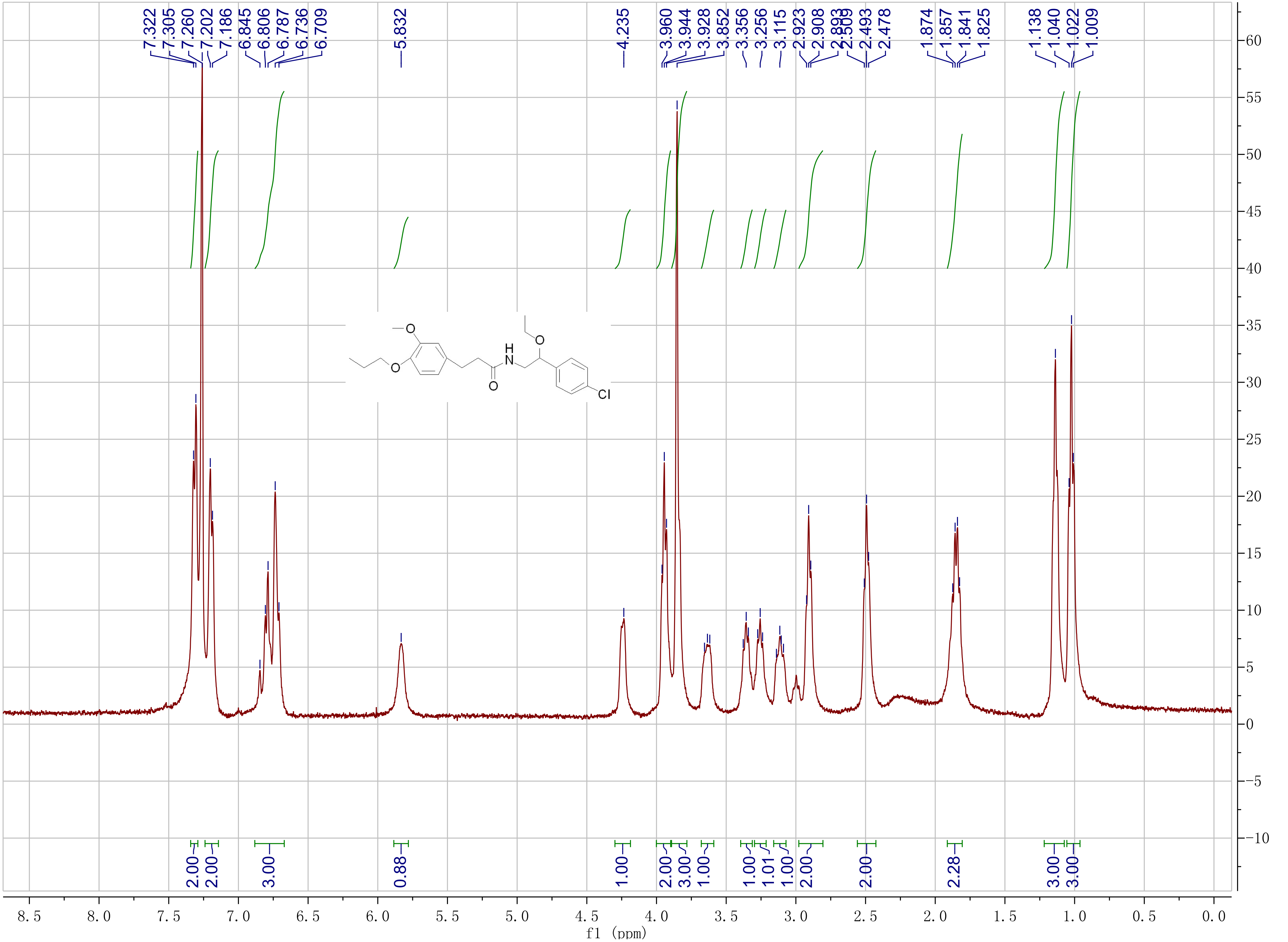
7.322
7.305
7.260
7.202
7.186
6.845
6.806
6.787
6.736
6.709
5.832
4.235
3.960
3.944
3.928
3.852
3.356
3.256
3.115
2.923
2.908
2.893
2.509
2.493
2.478
1.874
1.857
1.841
1.825
1.138
1.040
1.022
1.009
O
O
H
N
O
O
Cl
2.00
2.00
3.00
0.88
1.00
2.00
3.00
1.00
1.00
1.01
1.00
2.00
2.00
2.28
3.00
3.00
8.5
8.0
7.5
7.0
6.5
6.0
5.5
5.0
4.5
4.0
3.5
3.0
2.5
2.0
1.5
1.0
0.5
0.0
f1 (ppm)
60
55
50
45
40
35
30
25
20
15
10
5
0
−5
−10

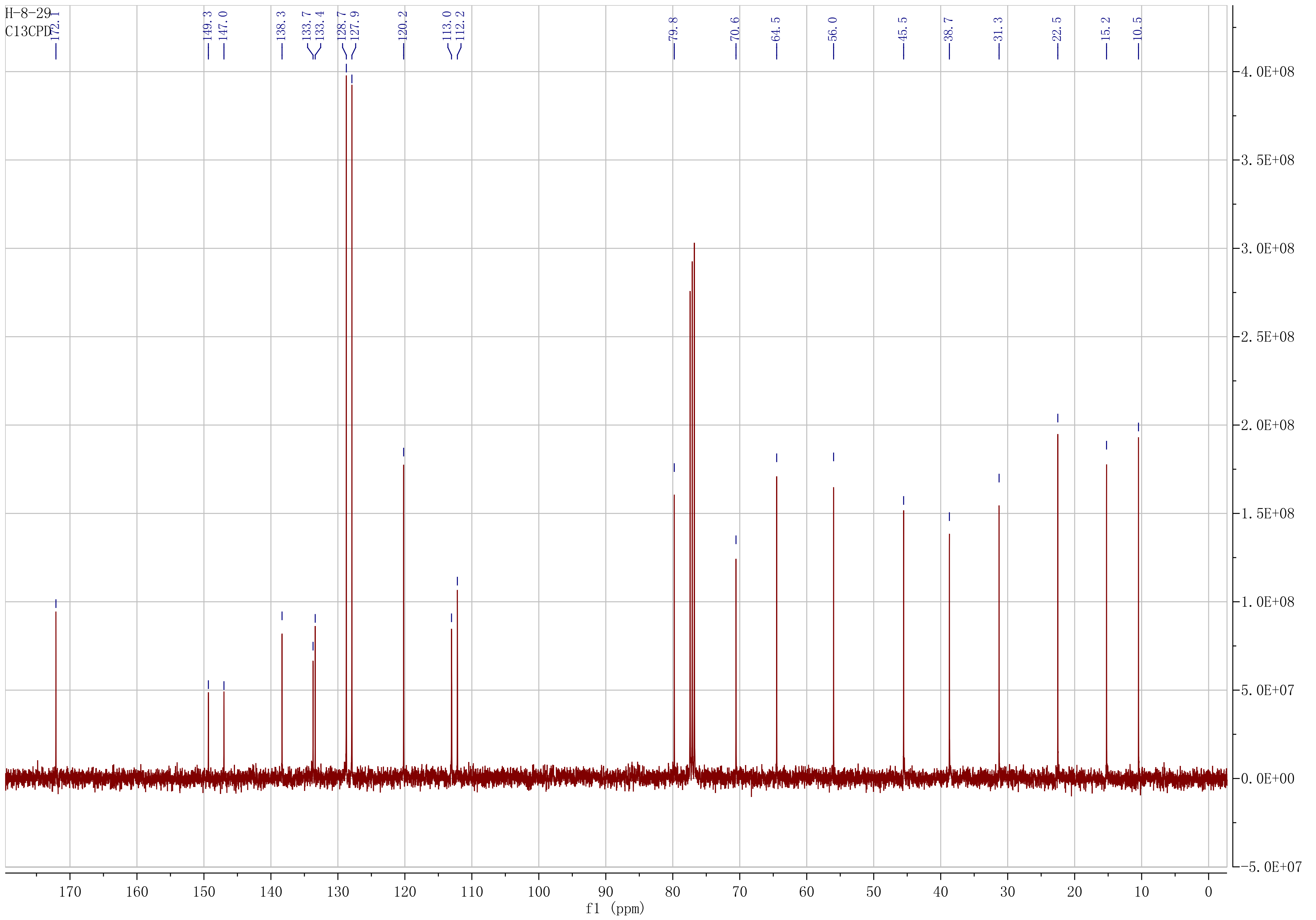
H-8-29
C13CPD
172.1
149.3
147.0
138.3
133.7
133.4
128.7
127.9
120.2
113.0
112.2
79.8
70.6
64.5
56.0
45.5
38.7
31.3
22.5
15.2
10.5
4.0E+08
3.5E+08
3.0E+08
2.5E+08
2.0E+08
1.5E+08
1.0E+08
5.0E+07
0.0E+00
-5.0E+07
170
160
150
140
130
120
110
100
90
80
70
60
50
40
30
20
10
0
f1 (ppm)

| | | | | | | | |
|---|---|---|---|---|---|---|---|
| **Sample Name** | lc/ms | **Position** | P1-A2 | **Instrument Name** | Instrument 1 | **User Name** | |
| **Inj Vol** | -1 | **InjPosition** | | **SampleType** | Sample | **IRM Calibration Status** | Some Ions Missed |
| **Data Filename** | H-8-29.d | **ACQ Method** | | **Comment** | | **Acquired Time** | 4/9/2009 9:09:32 AM |

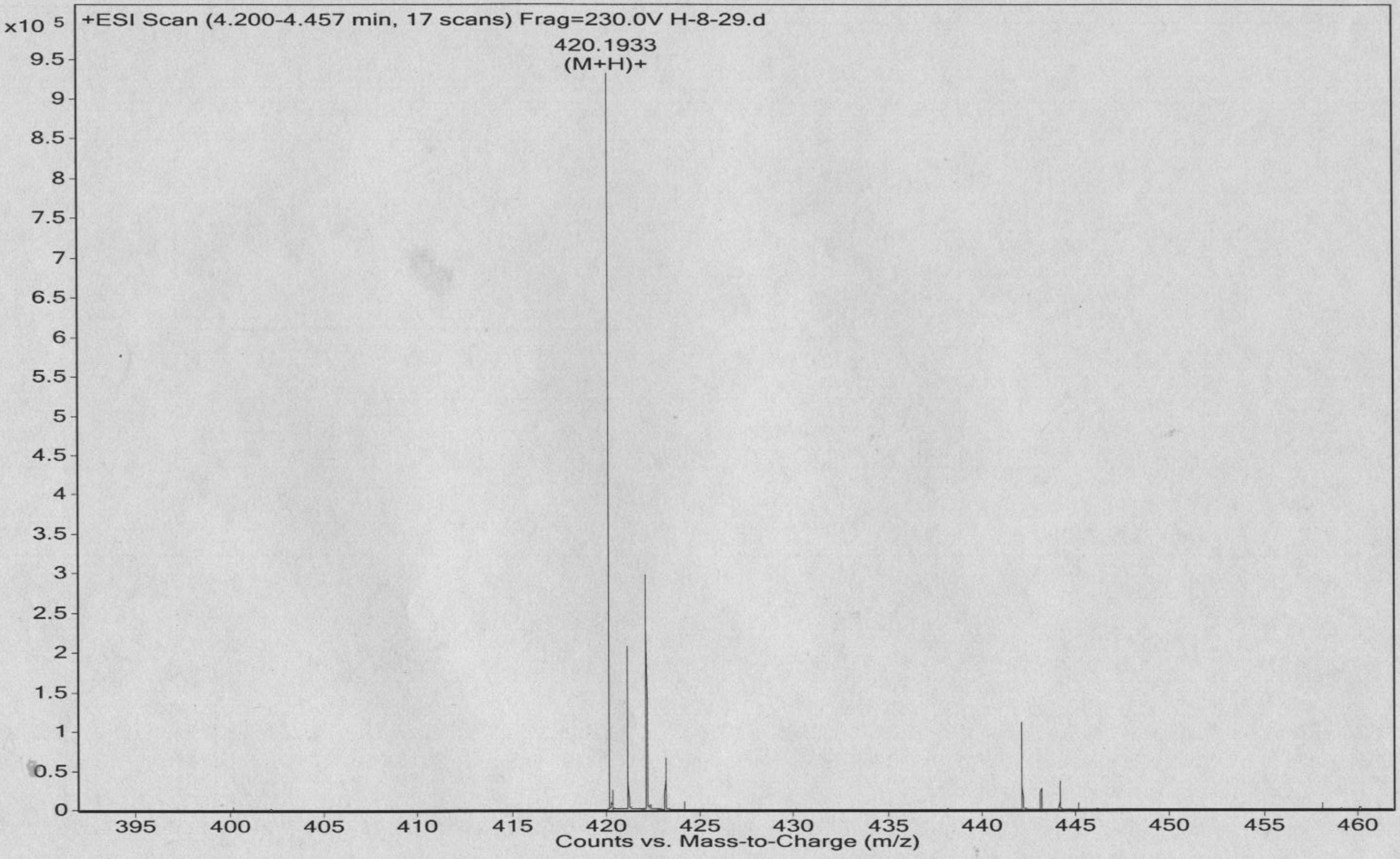

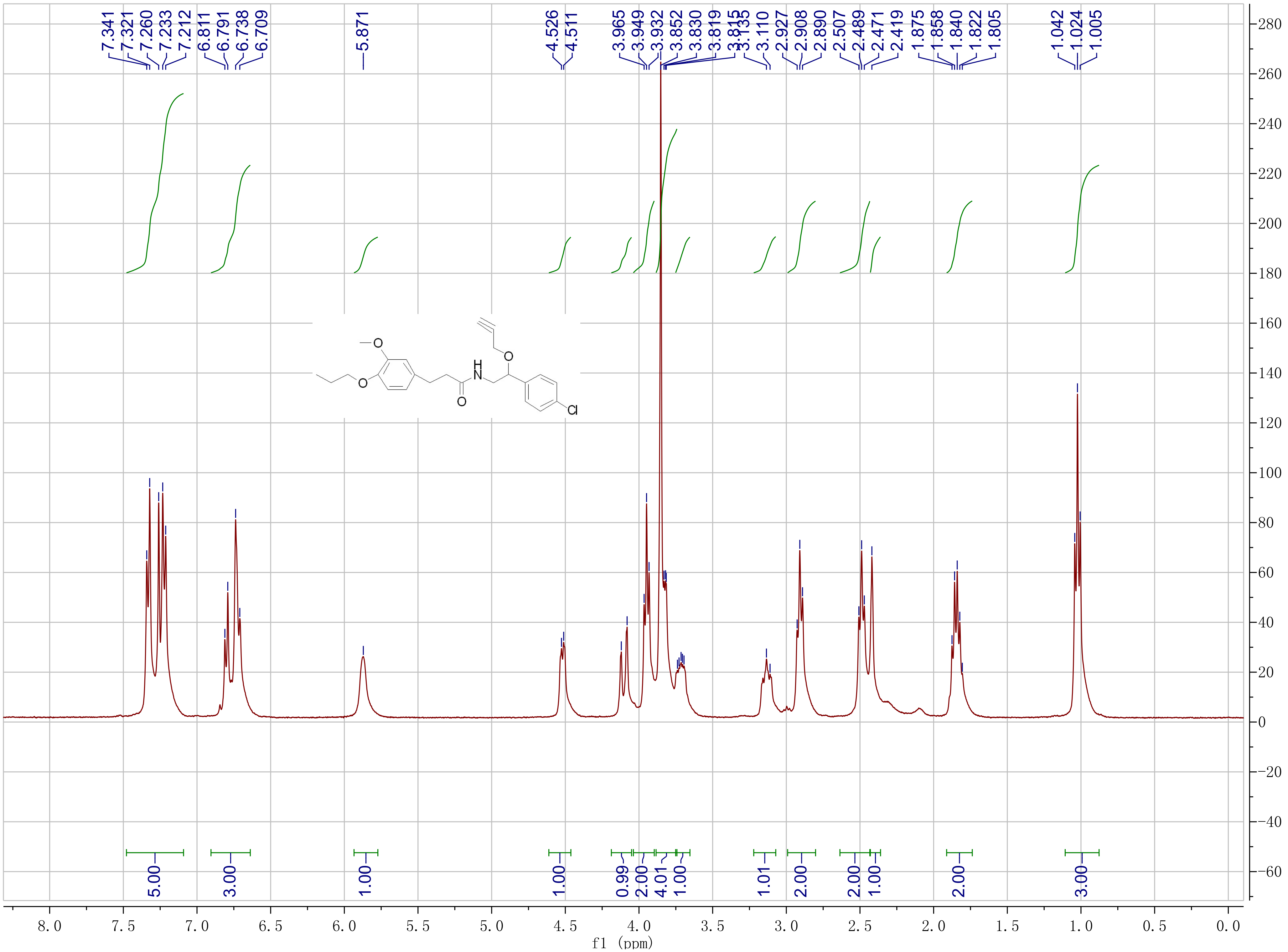

7.341
7.321
7.260
7.233
7.212
6.811
6.791
6.738
6.709
5.871
4.526
4.511
3.965
3.949
3.932
3.852
3.830
3.819
3.135
3.110
2.927
2.908
2.890
2.507
2.489
2.471
2.419
1.875
1.858
1.840
1.822
1.805
1.042
1.024
1.005
5.00
3.00
1.00
1.00
0.99
2.00
4.01
1.00
1.01
2.00
2.00
1.00
2.00
3.00
f1 (ppm)

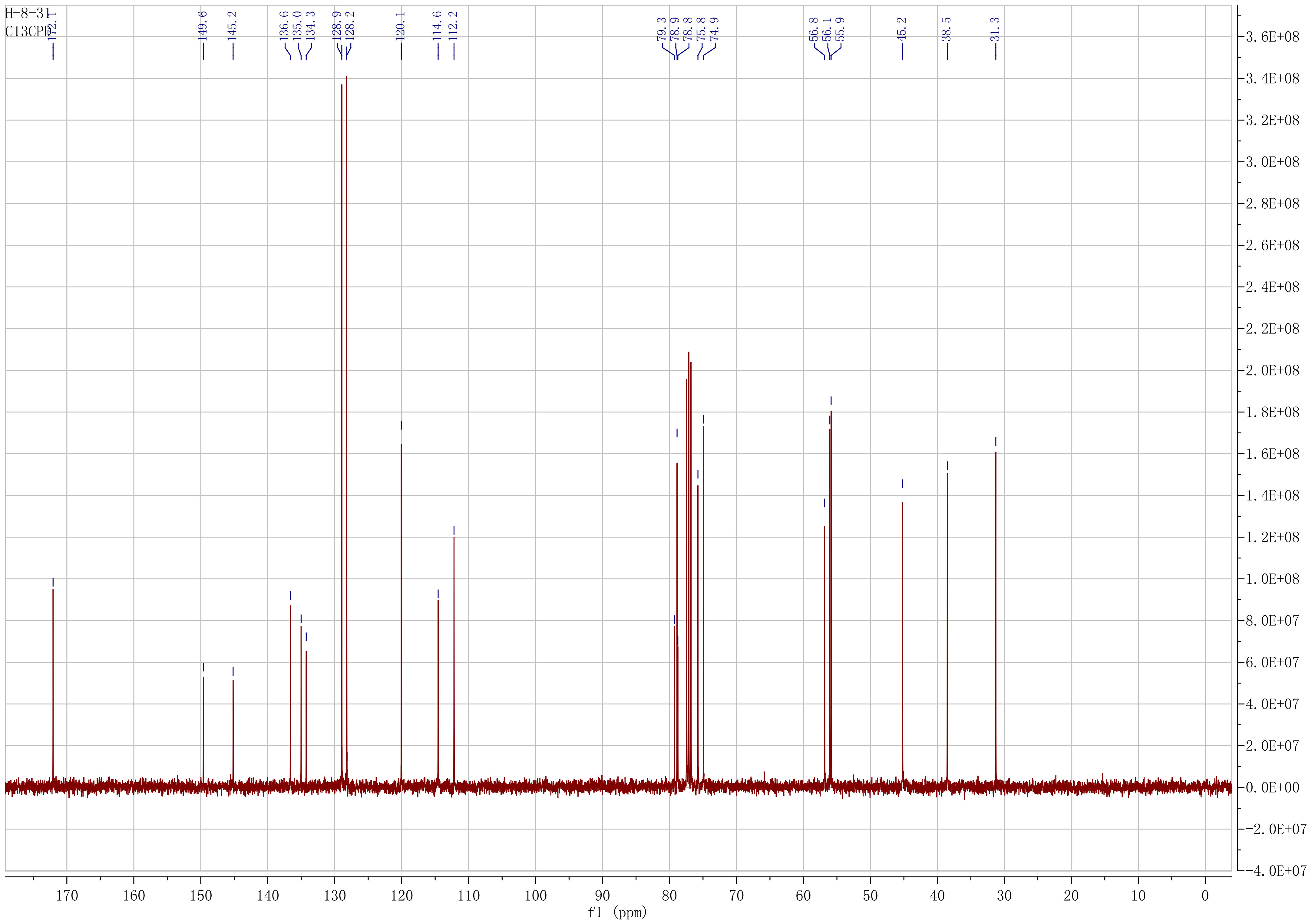
H-8-31
C13CPD
172.1
149.6
145.2
136.6
135.0
134.3
128.9
128.2
120.1
114.6
112.2
79.3
78.9
78.8
75.8
74.9
56.8
56.1
55.9
45.2
38.5
31.3
f1 (ppm)

| | | | | | | | |
|---|---|---|---|---|---|---|---|
| **Sample Name** | lc/ms | **Position** | Vial 3 | **Instrument Name** | Instrument 1 | **User Name** | |
| **Inj Vol** | -1 | **InjPosition** | | **SampleType** | Sample | **IRM Calibration Status** | Some Ions Missed |
| **Data Filename** | H-8-26.d | **ACQ Method** | | **Comment** | | **Acquired Time** | 4/9/2009 8:51:20 AM |

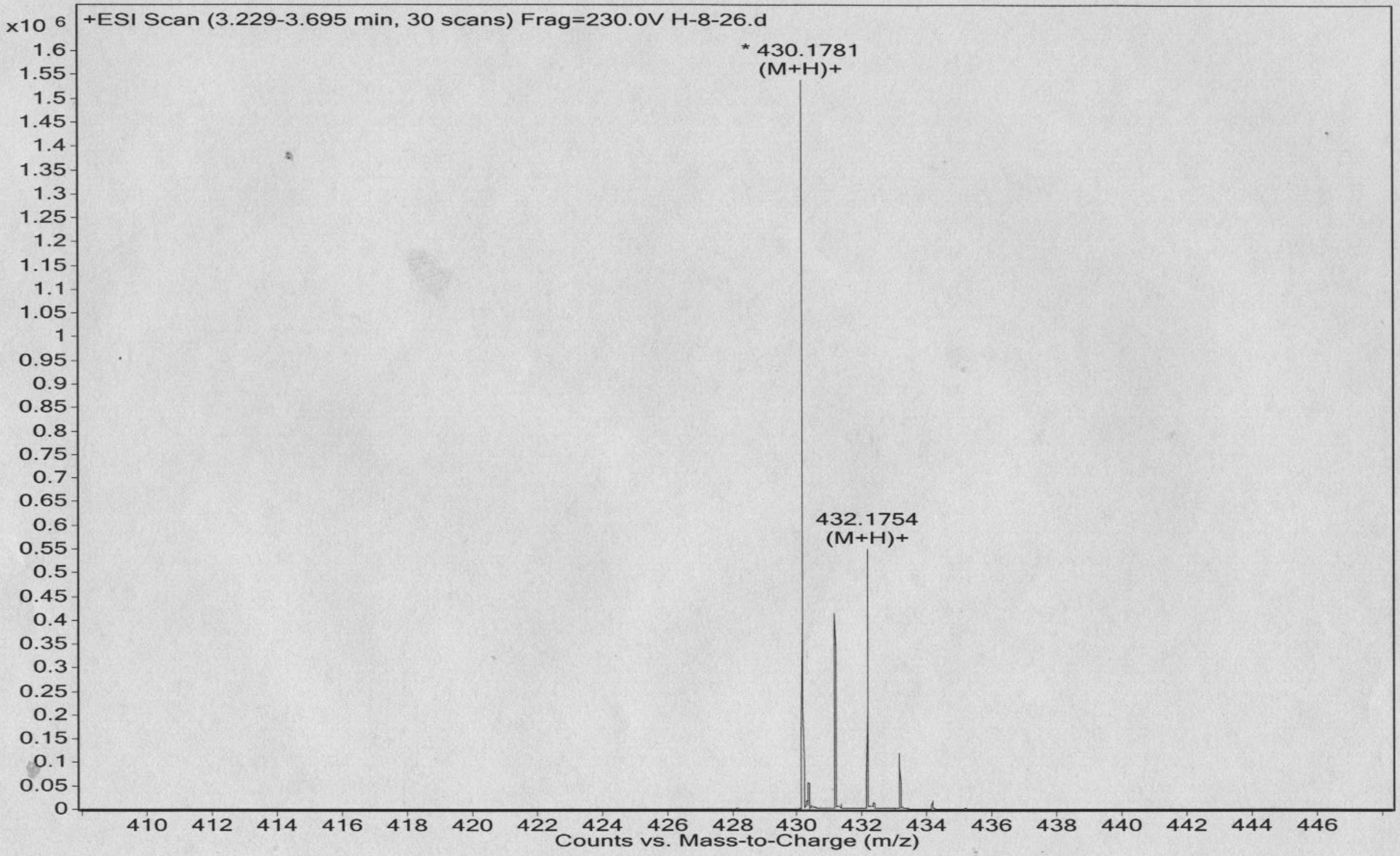

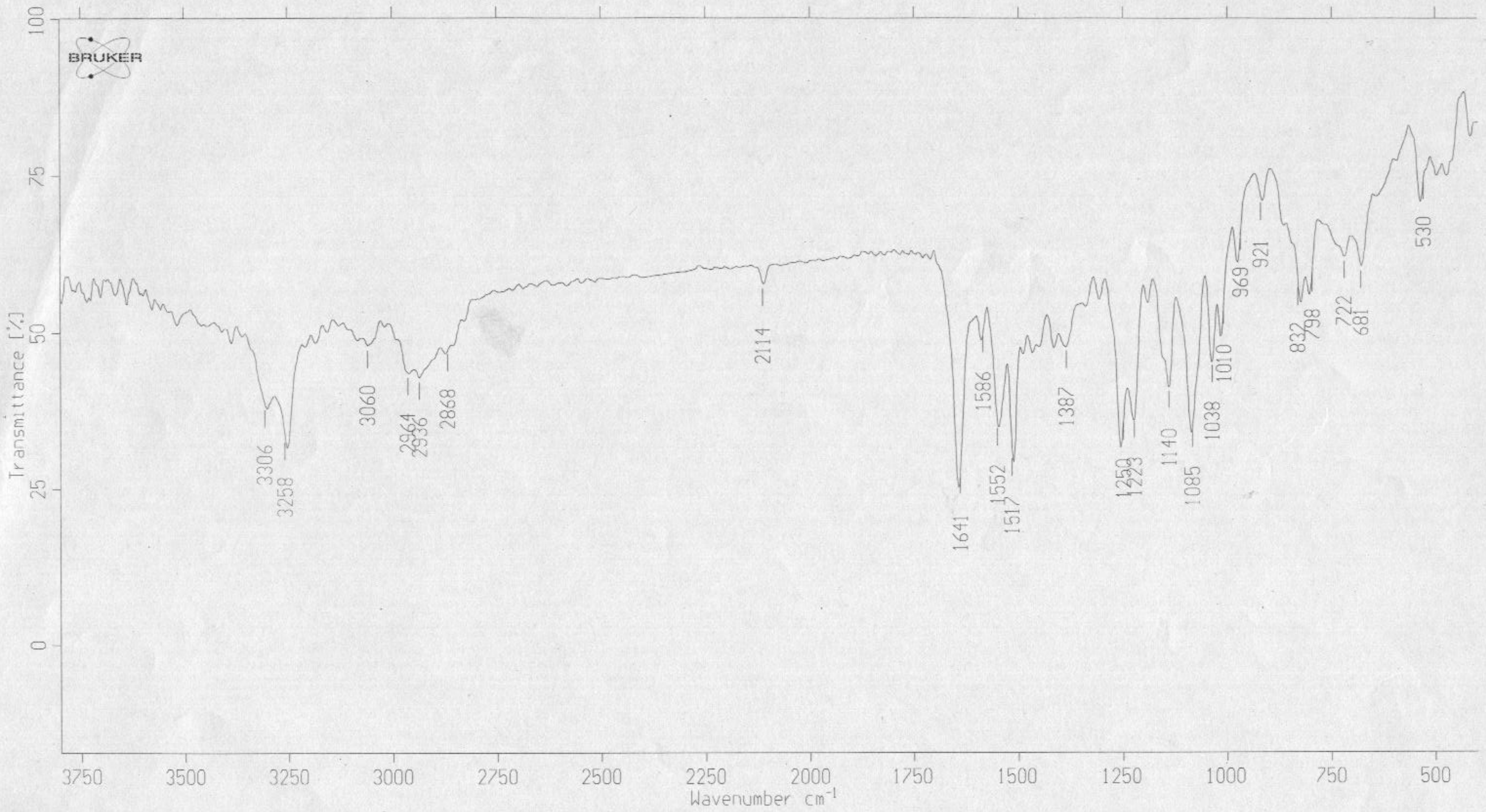


sample : H8-26

technique :

customer :

comments :

freq. range : 399.1926 - 3999.6401

resolution : 4.0

zerofilling : 2

Instrument : EQUINOX55

acqu. m : Double Sided Forward-Backward

sample scans : 30

measured : 20/4/2009

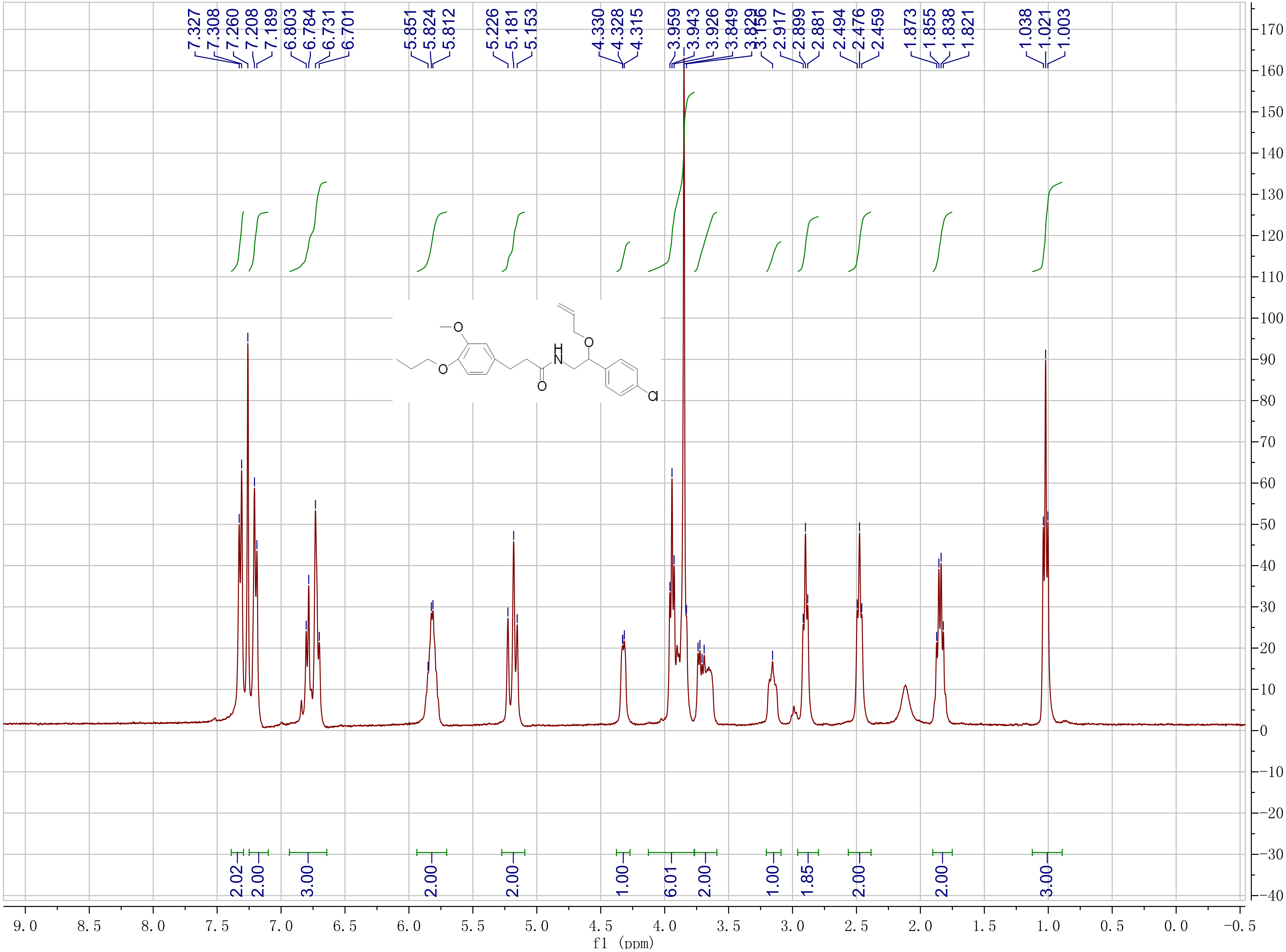

7.327
7.308
7.260
7.208
7.189
6.803
6.784
6.731
6.701
5.851
5.824
5.812
5.226
5.181
5.153
4.330
4.328
4.315
3.959
3.943
3.926
3.849
2.917
2.899
2.881
2.494
2.476
2.459
1.873
1.855
1.838
1.821
1.038
1.021
1.003
2.02
2.00
3.00
2.00
2.00
1.00
6.01
2.00
1.00
1.85
2.00
2.00
3.00
O
H
N
O
O
O
Cl
f1 (ppm)

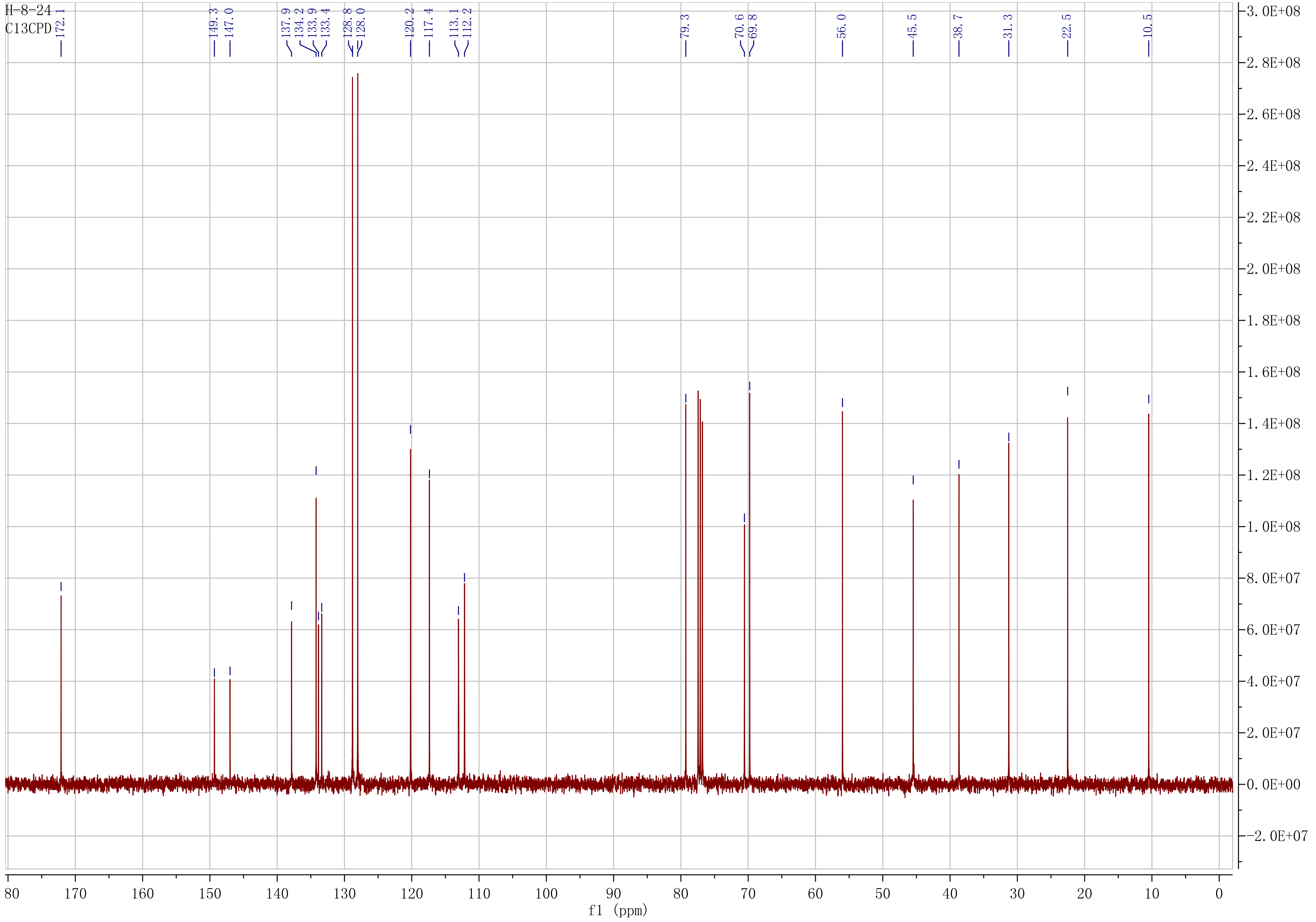
H-8-24
C13CPD
172.1
149.3
147.0
137.9
134.2
133.9
133.4
128.8
128.0
120.2
117.4
113.1
112.2
79.3
70.6
69.8
56.0
45.5
38.7
31.3
22.5
10.5
f1 (ppm)

| | | | | | | | |
|---|---|---|---|---|---|---|---|
| **Sample Name** | LC-HRMS | **Position** | P1-A4 | **Instrument Name** | Instrument 1 | **User Name** | |
| **Inj Vol** | -1 | **InjPosition** | | **SampleType** | Sample | **IRM Calibration Status** | Some Ions Missed |
| **Data Filename** | H-8-24.d | **ACQ Method** | chen-ms.m | **Comment** | | **Acquired Time** | 4/9/2009 2:56:08 PM |

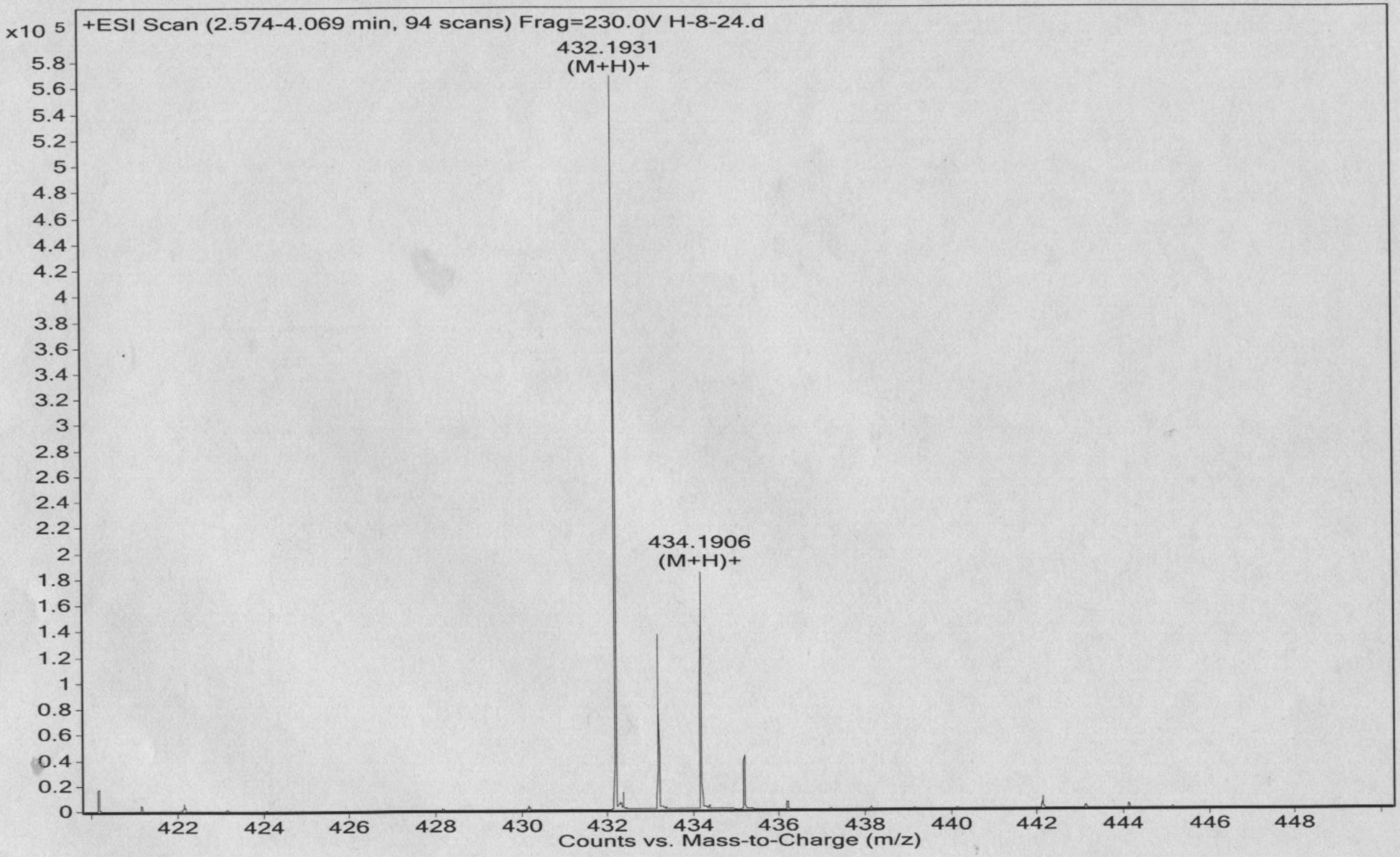

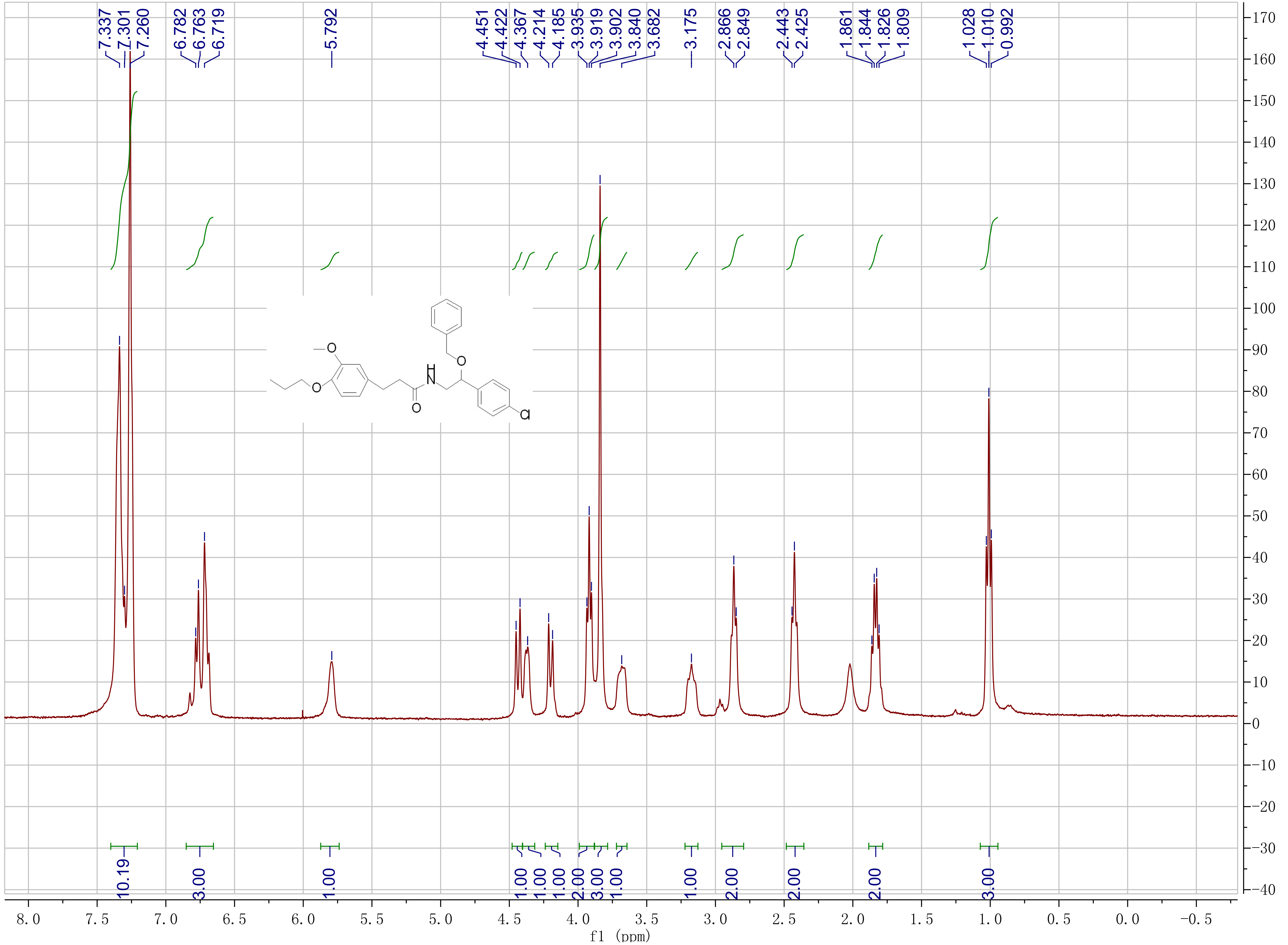

7.337
7.301
7.260
6.782
6.763
6.719
5.792
4.451
4.422
4.367
4.214
4.185
3.935
3.919
3.902
3.840
3.682
3.175
2.866
2.849
2.443
2.425
1.861
1.844
1.826
1.809
1.028
1.010
0.992
O
H
N
O
O
O
Cl
10.19
3.00
1.00
1.00
1.00
1.00
2.00
3.00
1.00
1.00
2.00
2.00
2.00
3.00
8.0
7.5
7.0
6.5
6.0
5.5
5.0
4.5
4.0
3.5
3.0
2.5
2.0
1.5
1.0
0.5
0.0
-0.5
f1 (ppm)
170
160
150
140
130
120
110
100
90
80
70
60
50
40
30
20
10
0
-10
-20
-30
-40

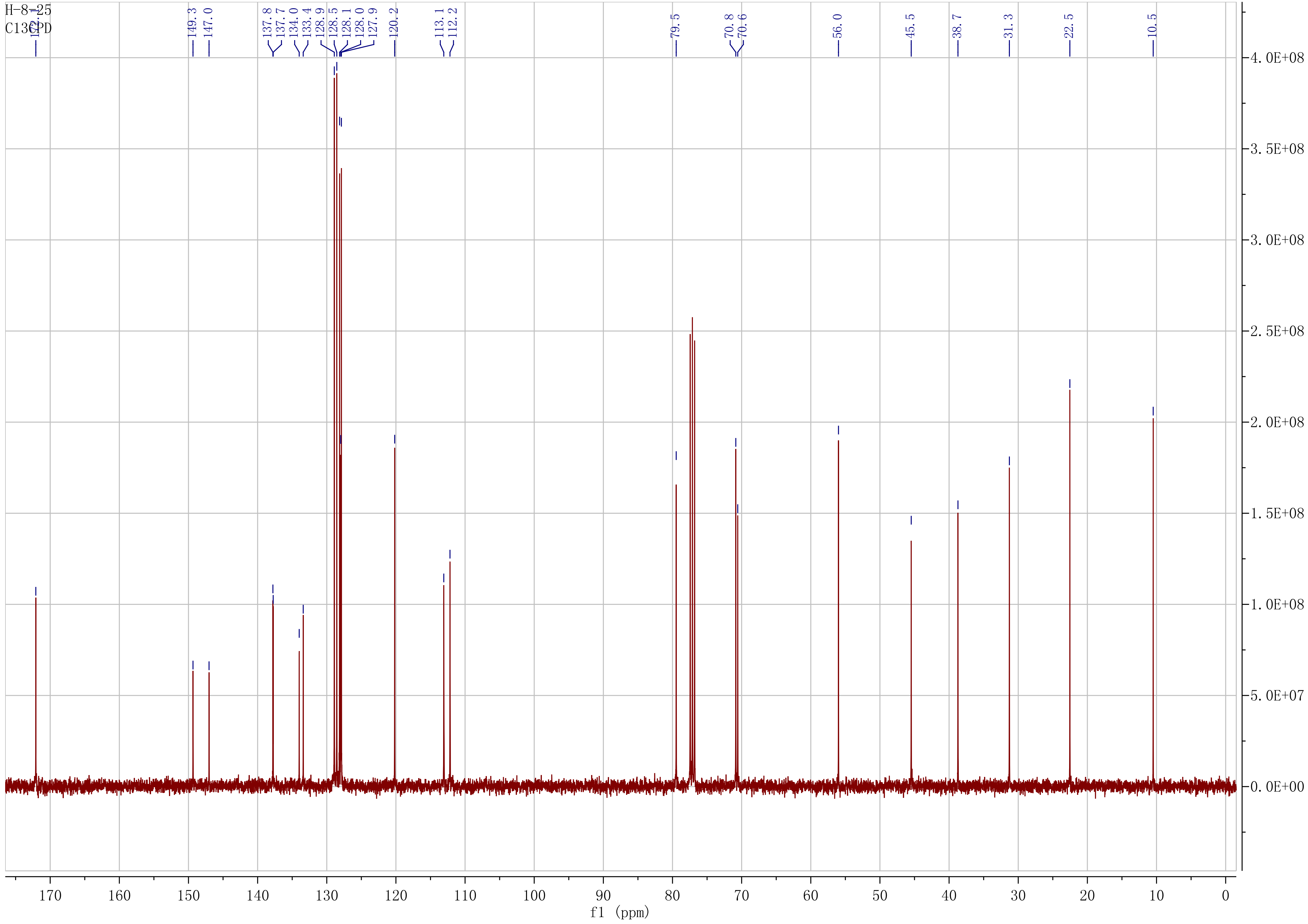

H-8-25
C13CPD
172.1
149.3
147.0
137.8
137.7
134.0
133.4
128.9
128.5
128.1
128.0
127.9
120.2
113.1
112.2
79.5
70.8
70.6
56.0
45.5
38.7
31.3
22.5
10.5
4.0E+08
3.5E+08
3.0E+08
2.5E+08
2.0E+08
1.5E+08
1.0E+08
5.0E+07
0.0E+00
170
160
150
140
130
120
110
100
90
80
70
60
50
40
30
20
10
0
f1 (ppm)

| | | | | | | | |
|---|---|---|---|---|---|---|---|
| **Sample Name** | lc/ms | **Position** | Vial 3 | **Instrument Name** | Instrument 1 | **User Name** | |
| **Inj Vol** | -1 | **InjPosition** | | **SampleType** | Sample | **IRM Calibration Status** | Some Ions Missed |
| **Data Filename** | H-8-25.d | **ACQ Method** | | **Comment** | | **Acquired Time** | 4/9/2009 8:43:54 AM |

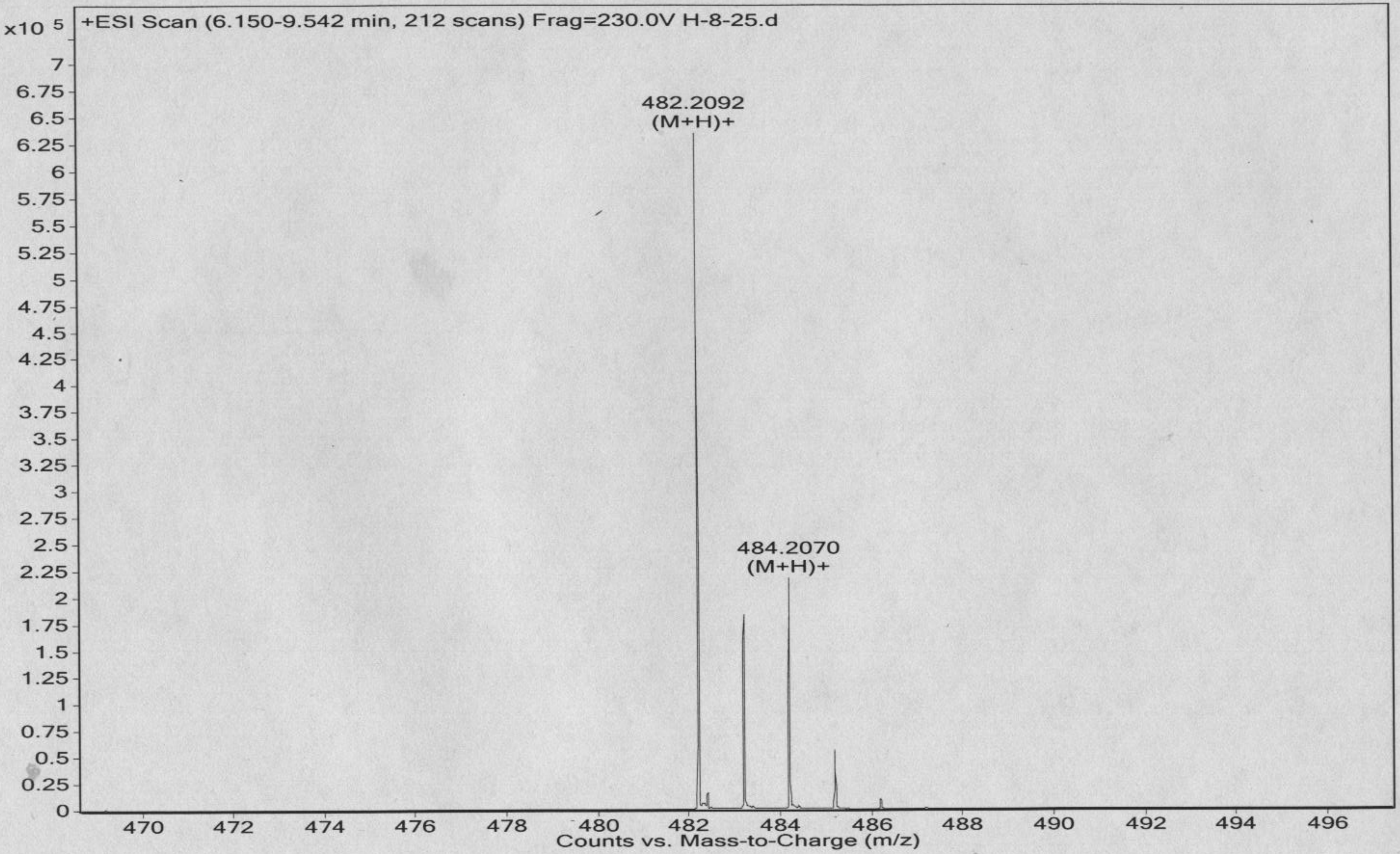

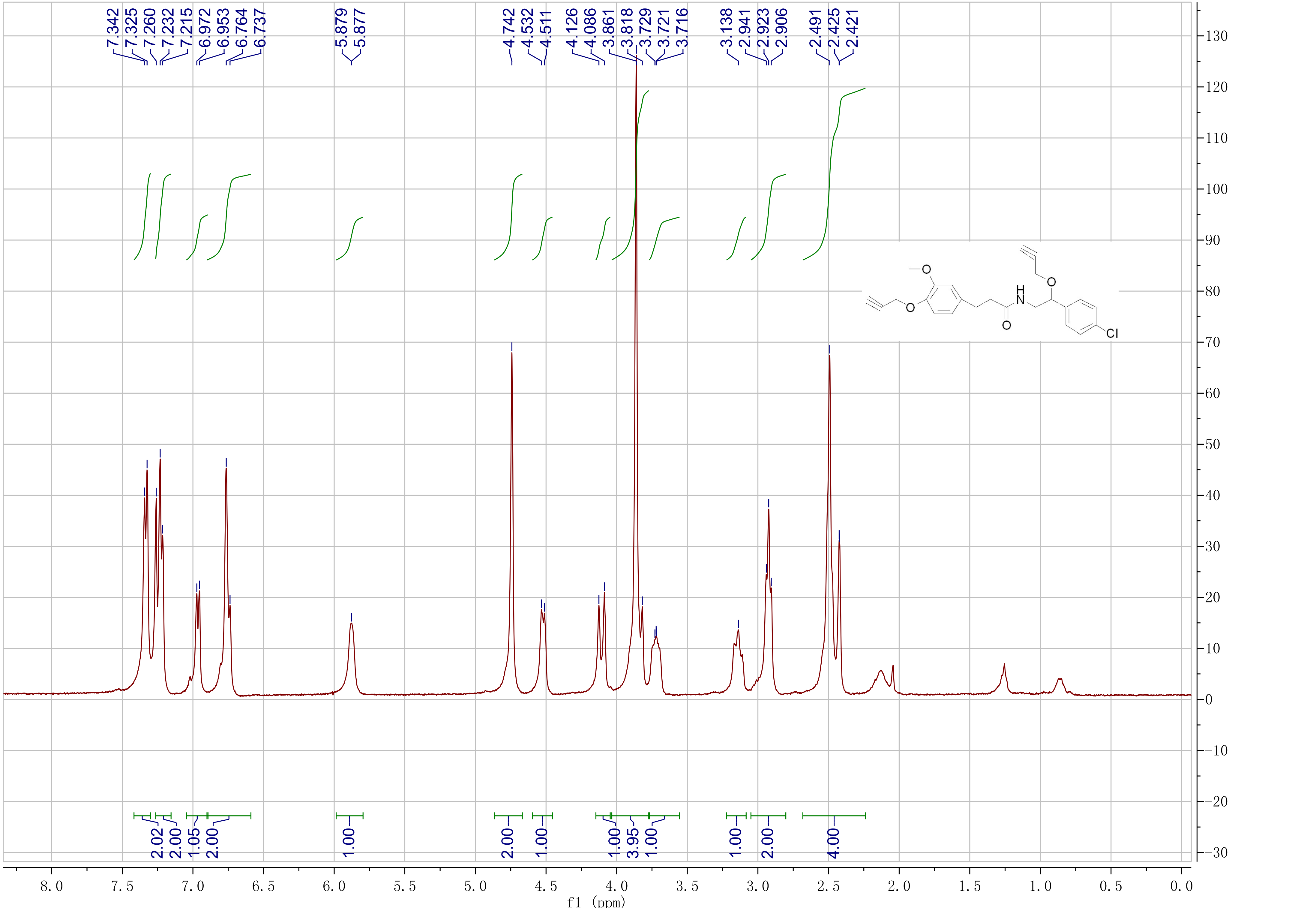
7.342
7.325
7.260
7.232
7.215
6.972
6.953
6.764
6.737
5.879
5.877
4.742
4.532
4.511
4.126
4.086
3.861
3.818
3.729
3.721
3.716
3.138
2.941
2.923
2.906
2.491
2.425
2.421
2.02
2.00
1.05
2.00
1.00
2.00
1.00
1.00
3.95
1.00
1.00
2.00
4.00
O
O
O
H
N
O
Cl
8.0
7.5
7.0
6.5
6.0
5.5
5.0
4.5
4.0
3.5
3.0
2.5
2.0
1.5
1.0
0.5
0.0
f1 (ppm)
130
120
110
100
90
80
70
60
50
40
30
20
10
0
-10
-20
-30

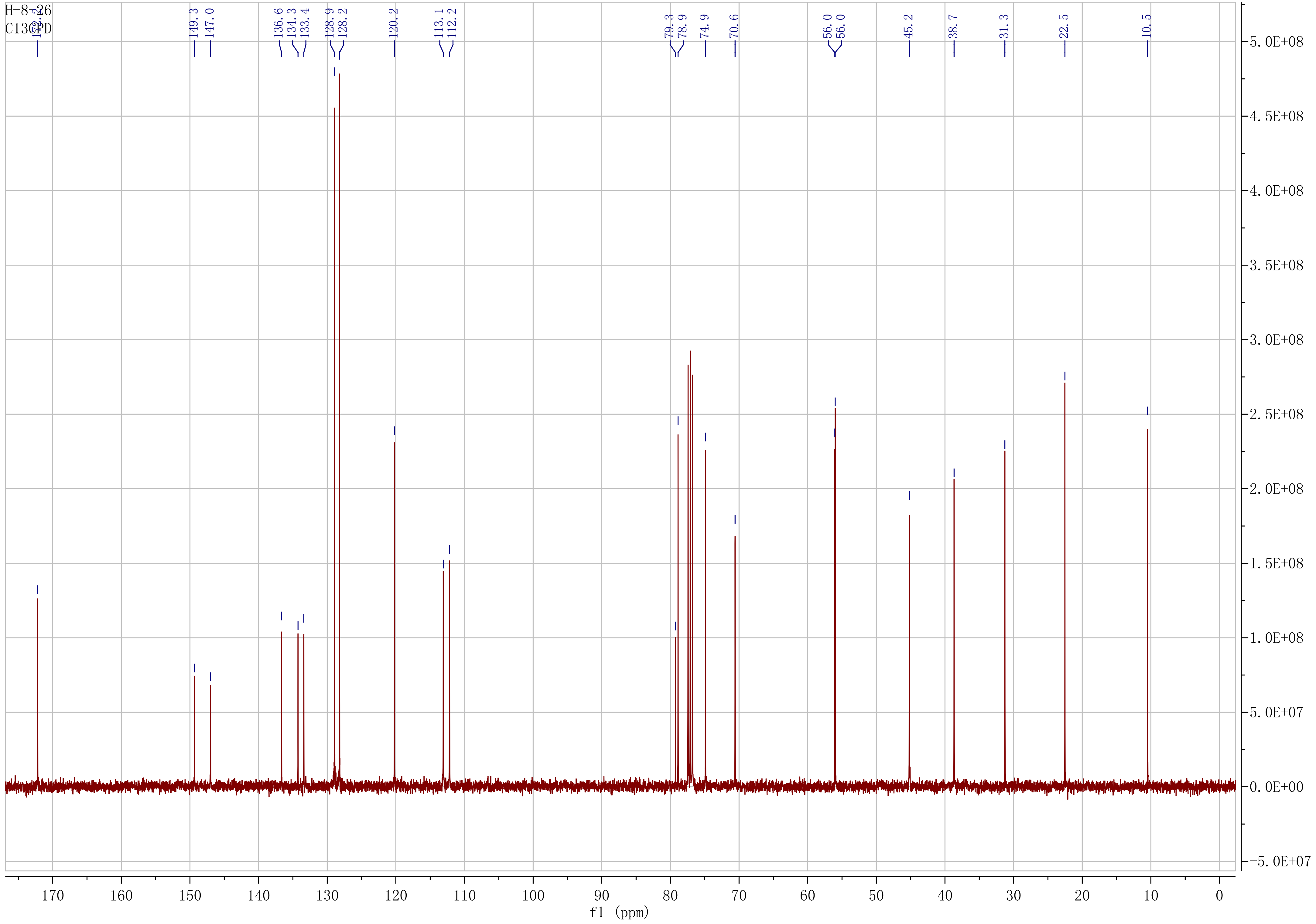
H-8-26
C13CPD
172.2
149.3
147.0
136.6
134.3
133.4
128.9
128.2
120.2
113.1
112.2
79.3
78.9
74.9
70.6
56.0
56.0
45.2
38.7
31.3
22.5
10.5
5.0E+08
4.5E+08
4.0E+08
3.5E+08
3.0E+08
2.5E+08
2.0E+08
1.5E+08
1.0E+08
5.0E+07
0.0E+00
−5.0E+07
170
160
150
140
130
120
110
100
90
80
70
60
50
40
30
20
10
0
f1 (ppm)

| | | | | | | | |
|---|---|---|---|---|---|---|---|
| **Sample Name** | lc/ms | **Position** | P1-A4 | **Instrument Name** | Instrument 1 | **User Name** | |
| **Inj Vol** | -1 | **InjPosition** | | **SampleType** | Sample | **IRM Calibration Status** | Some Ions Missed |
| **Data Filename** | H-8-31.d | **ACQ Method** | | **Comment** | | **Acquired Time** | 4/9/2009 9:34:31 AM |

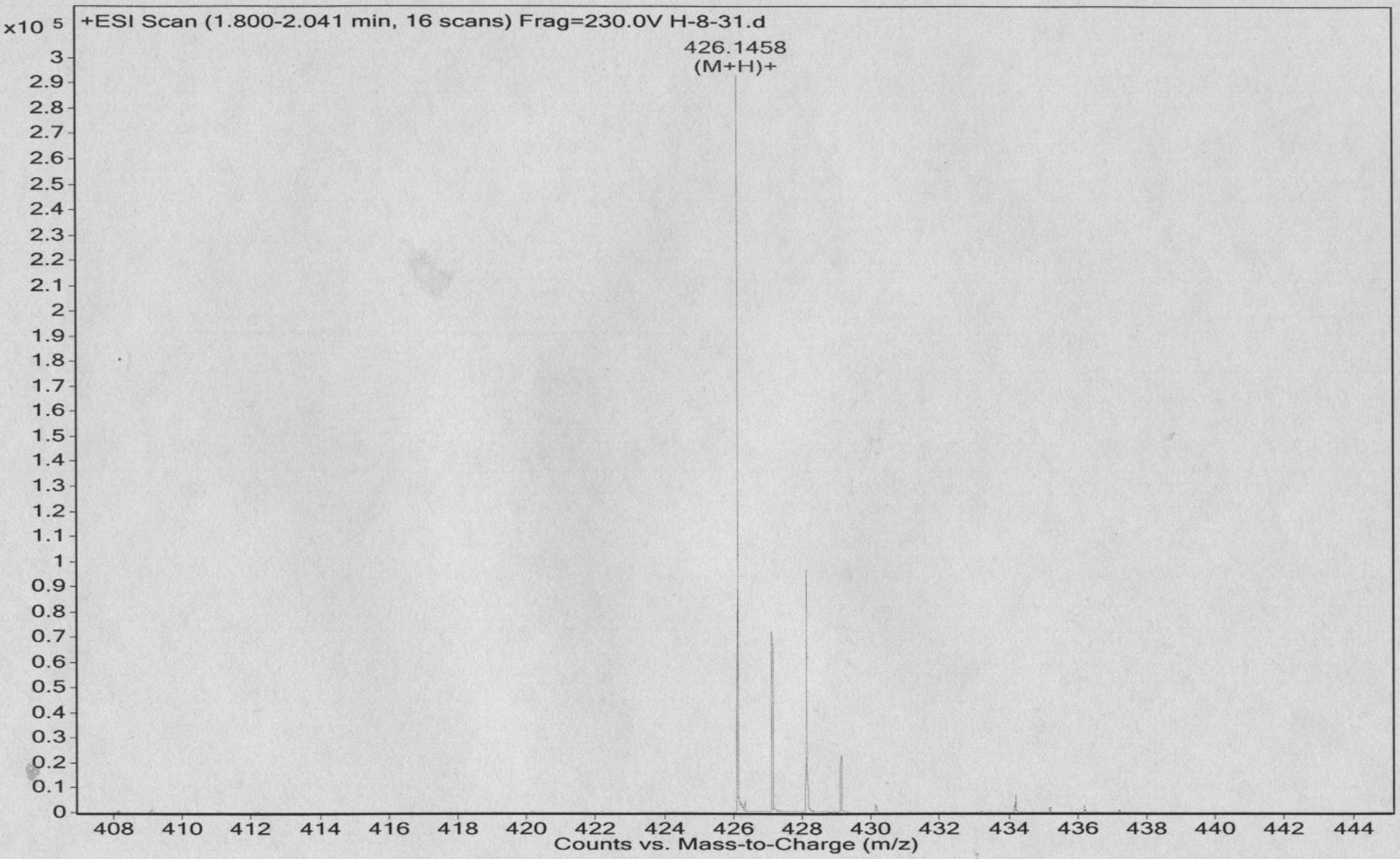

Supplement: Additional file 1 — Copies of 1 H NMR, 13 C NMR and HRMS. [file 1752-153X-7-33-S1.pdf]
